# A complete *Leishmania donovani* reference genome identifies novel genetic variations associated with virulence

**DOI:** 10.1038/s41598-018-34812-x

**Published:** 2018-11-08

**Authors:** Patrick Lypaczewski, Johanna Hoshizaki, Wen-Wei Zhang, Laura-Isobel McCall, John Torcivia-Rodriguez, Vahan Simonyan, Amanpreet Kaur, Ken Dewar, Greg Matlashewski

**Affiliations:** 1 Department of Microbiology and Immunology, McGill University, 3775 University Street, H3A 2B4 Montreal Quebec, Canada; 2 Present Address: Department of Chemistry and Biochemistry, University of Oklahoma, Norman, OK USA; 3 Center for Biologics Evaluation and Research, Food and Drug Administration, Maryland, USA; 4 Department of Human Genetics, McGill University, Montreal, Quebec, Canada; 5 McGill University and Genome Quebec Innovation Center, McGill University, Montreal, Quebec, Canada

## Abstract

*Leishmania donovani* is responsible for visceral leishmaniasis, a neglected and lethal parasitic disease with limited treatment options and no vaccine. The study of *L*. *donovani* has been hindered by the lack of a high-quality reference genome and this can impact experimental outcomes including the identification of virulence genes, drug targets and vaccine development. We therefore generated a complete genome assembly by deep sequencing using a combination of second generation (Illumina) and third generation (PacBio) sequencing technologies. Compared to the current *L*. *donovani* assembly, the genome assembly reported within resulted in the closure over 2,000 gaps, the extension of several chromosomes up to telomeric repeats and the re-annotation of close to 15% of protein coding genes and the annotation of hundreds of non-coding RNA genes. It was possible to correctly assemble the highly repetitive A2 and Amastin virulence gene clusters. A comparative sequence analysis using the improved reference genome confirmed 70 published and identified 15 novel genomic differences between closely related visceral and atypical cutaneous disease-causing *L*. *donovani* strains providing a more complete map of genes associated with virulence and visceral organ tropism. Bioinformatic tools including protein variation effect analyzer and basic local alignment search tool were used to prioritize a list of potential virulence genes based on mutation severity, gene conservation and function. This complete genome assembly and novel information on virulence factors will support the identification of new drug targets and the development of a vaccine for *L*. *donovani*.

## Introduction

Visceral Leishmaniasis (VL) is the second most lethal parasitic disease following malaria and is prevalent throughout underdeveloped and tropical regions of the world. There are some 300,000 new cases each year^1^ and *Leishmania donovani*, transmitted by the infected sand fly, is the major causative agent of VL in the Indian and African continents. Although *L*. *donovani* is extensively studied, its genome remains poorly annotated because it is heavily fragmented and a complete assembly is crucial to understanding this parasite’s biology, metabolic pathways, tissue tropism and disease pathology.

The pathology of leishmaniasis is predominantly parasite species-specific, such as for example *L*. *major* that causes cutaneous leishmaniasis (CL) whereas *L*. *donovani* typically causes lethal visceral leishmaniasis (VL). Previous studies have compared genomes of *L*. *major* and *L*. *donovani* parasites to study virulence and disease tropism and have identified a number of species specific genes including A2 present in *L*. *donovani* that is a pseudogene in *L*. *major*^2,3^. More recently, as cases of atypical CL caused by *L*. *donovani* have emerged, studies have compared cutaneous and visceral disease-causing strains of *L*. *donovani*, as these strains provide a unique opportunity to study the genetic determinants of disease pathogenesis using more recently diverged strains^4^.

Second-generation sequencing technologies including Illumina, have made the sequencing of large genomes feasible through the mapping of short sequence reads of 50 to 250 nucleotides (nt) to a reference genome^5^. While human and many other well studied higher vertebrates have better assembled reference genomes^6^, the kinetoplastids suffer in this regard because most *Leishmania* species either lack sequencing information altogether or have incomplete reference genomes with sometimes thousands of sequence gaps^7^. The current *L*. *donovani* reference genome (ASM22713v2 from strain BPK282A^8^) was generated using second generation technologies and contains over 2,000 gaps and therefore there are many incomplete or inaccurate protein coding sequences. The first complete *Leishmania* genome generated is that of *L*. *major* by a consortium of laboratories employing large insert clone tiling paths to sequence each chromosome individually^9,10^. This genome was later improved by the reassembly of complex collapsed loci that were incorrect in the original reference genome^11^.

Since then however, advances in sequencing technologies have drastically reduced the cost of sequencing and eased genome assembly tasks by increasing the length of the generated sequences. Long read sequencing or “third-generation” sequencing refers to more recent technologies including Oxford NanoPore^12^ and Pacific Biosciences (PacBio)^13^ that can result in reads ranging up to 50 kb or 100 kb that are capable of generating more complete genomic assemblies, provided the read lengths traverse across repetitive elements. One such highly repetitive cluster is the A2 gene family from *L*. *donovani* considered to be an important virulence factor and is necessary for survival in visceral organs^14,15^ and protection against host response stress^16,17^. Due to its repetitive nature, the A2 gene cluster is misassembled in all *Leishmania* genomes generated using second generation sequencing, and only resolved in a recent resequencing effort targeted to *L*. *infantum* exploiting the long-read capabilities of PacBio sequencing which resulted in a complete genome assembly^18^. The current *L*. *donovani* genome however was obtained from second generation sequencing and consequently, no precise DNA or complete protein sequences are available for any A2 protein in *L*. *donovani*, hindering the comparison of A2 genes in visceral disease-causing strains or using mass spectrometry to identify A2 proteins which relies on accurate genome sequences for protein identification.

In this study, we have combined second and third generation sequencing to generate a complete assembly of the *L*. *donovani* genome from the strain responsible for cutaneous leishmaniasis (CL) in Sri Lanka^4,19^. This new assembly enabled the generation of an improved genome annotation and an unbiased analysis of chromosome synteny comparing *L*. *donovani* and *L*. *major* genes and strand switch transcription units. We have used this complete assembly to re-interrogate the genetic makeup of the visceral and cutaneous disease-causing *L*. *donovani* strains resulting in the identification of novel SNPs and indels generating a more complete and accurate chromosome map of the genetic differences between these phenotypically distinct *L*. *donovani* strains^4,20^. This study further enabled re-annotation of much of the genome highlighting the importance of a complete reference assembly to support future functional genomic and proteomic studies involving the *L*. *donovani* pathogen.

## Results

### A complete *L*. *donovani* genome assembly

The currently available assembly for *L*. *donovani* (ASM22713v2 from strain BPK282A^8^) contains over 2,000 gaps due to the presence of low complexity regions and the highly repetitive nature of the *Leishmania* genome^21^. This incomplete assembly makes it difficult to compare *L*. *donovani* genomes from strains with different phenotypic properties. DNA was therefore isolated from the attenuated cutaneous disease-causing strain of *L*. *donovani* from Sri Lanka^4^ and was subjected to deep sequencing using second and third generation sequencing. We reasoned that a complete assembly of the genome from this attenuated *L*. *donovani* strain will identify a more complete complement of genetic changes associated with loss of virulence of this strain. A total of 9 PacBio sequencing runs were performed generating 712,443 reads representing an estimated 107-fold coverage of the estimated 35 Mb genome. Importantly, there were 51,484 reads longer than 12 kb, representing a 20-fold coverage in very long reads. The long-read sequencing data was assembled using various assemblers as described in methods and merged using the longest chromosomes produced by each assembler followed by refinement using the high-quality short-read Illumina-generated data and iterative edge extension to close the remaining gaps.

**Figure 1:**
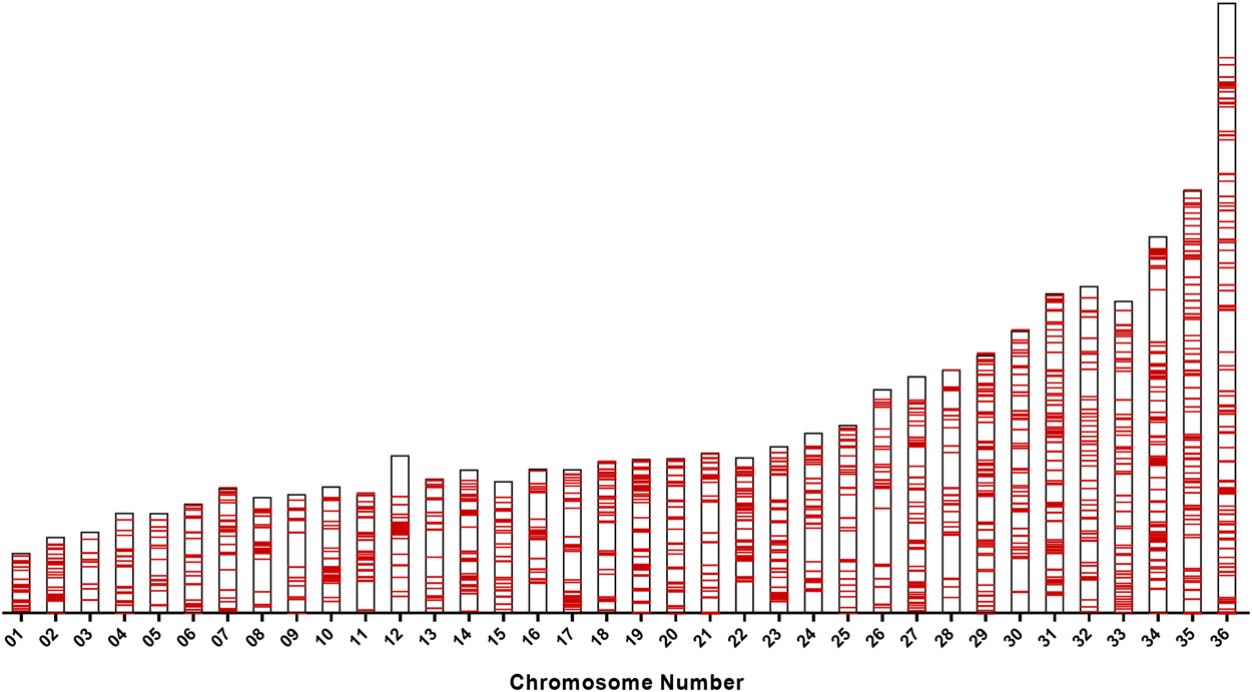
Location of the gaps along 36 chromosomes that have been closed in this new assembly. Chromosomal locations of gaps are indicated in red. No gaps remain in the current assembly.

The previous *L*. *donovani* reference assembly (ASM22713v2 from strain BPK282A) had over 2000 gaps spread across the 36 chromosomes. Figure 1 depicts the location on each chromosome of the gaps that have been closed in the new assembly reported here. The new assembly now contains contiguous DNA sequences in all 36 chromosomes and a corresponding 22-fold increase in N50 indicating that a larger proportion of the data has been assembled into large contigs as 50% of the genome is contained in contigs >= N50, resulting in an N50 of over 1Mbp (Table 1). Further, using this completed assembly, we have generated annotations for more potential protein coding regions than previously annotated (8,633 compared to 7,969 proteins) and identified more transfer-RNA and ribosomal RNA genes as well as all 6 small nuclear RNA genes, all spliced leader RNA genes and close to a thousand small nucleolar RNA genes. An additional 13 genes were marked as pseudogenes due to multiple internal stop codons and/or frameshifts. (Supplementary Table S1). Alignment of the second-generation Illumina reads to the PacBio generated assembly was used to cross-validate and correct the assembly at the nucleotide level. Graphs of the coverage from the alignment of Illumina and PacBio data across the 36 chromosomes are available in Supplementary Fig. S1. Taken together, we consider this new assembly to be contiguous and complete.

**Table 1:** Quality assessment metrics of the previous and current assemblies.

	Contigs	N50 (bp)	Protein coding	tRNA	rRNA	snRNA	SLRNA	snoRNA	Genes mapped
Old Assembly	2,154	45,436	7,969	64	11	4	—	31	8,081
New Assembly	36	1,067,468	8,633	90	51	6	68	910	9,758

Old assembly refers to ASM22713v2 from strain BPK282A, new assembly refers to the assembly presented in this work. Contigs denotes the number of genomic fragments uninterrupted by stretches of unknown bases (Ns) or chromosome ends. N50 is used as a measure of contiguity, 50% of the genome is contained in contigs of size N50 and above. Annotated genes were broken down into protein coding, transfer-RNA (tRNA), ribosomal RNA (rRNA), small nuclear RNA (snRNA), spliced leader RNA (SLRNA) and small nucleolar RNA (snoRNA) genes. The number of genes mapped indicates the number of annotated genes along the genome.

### Assembly of the A2 virulence gene cluster and synteny comparison between *L*. *major* and *L*. *donovani*

**Figure 2:**
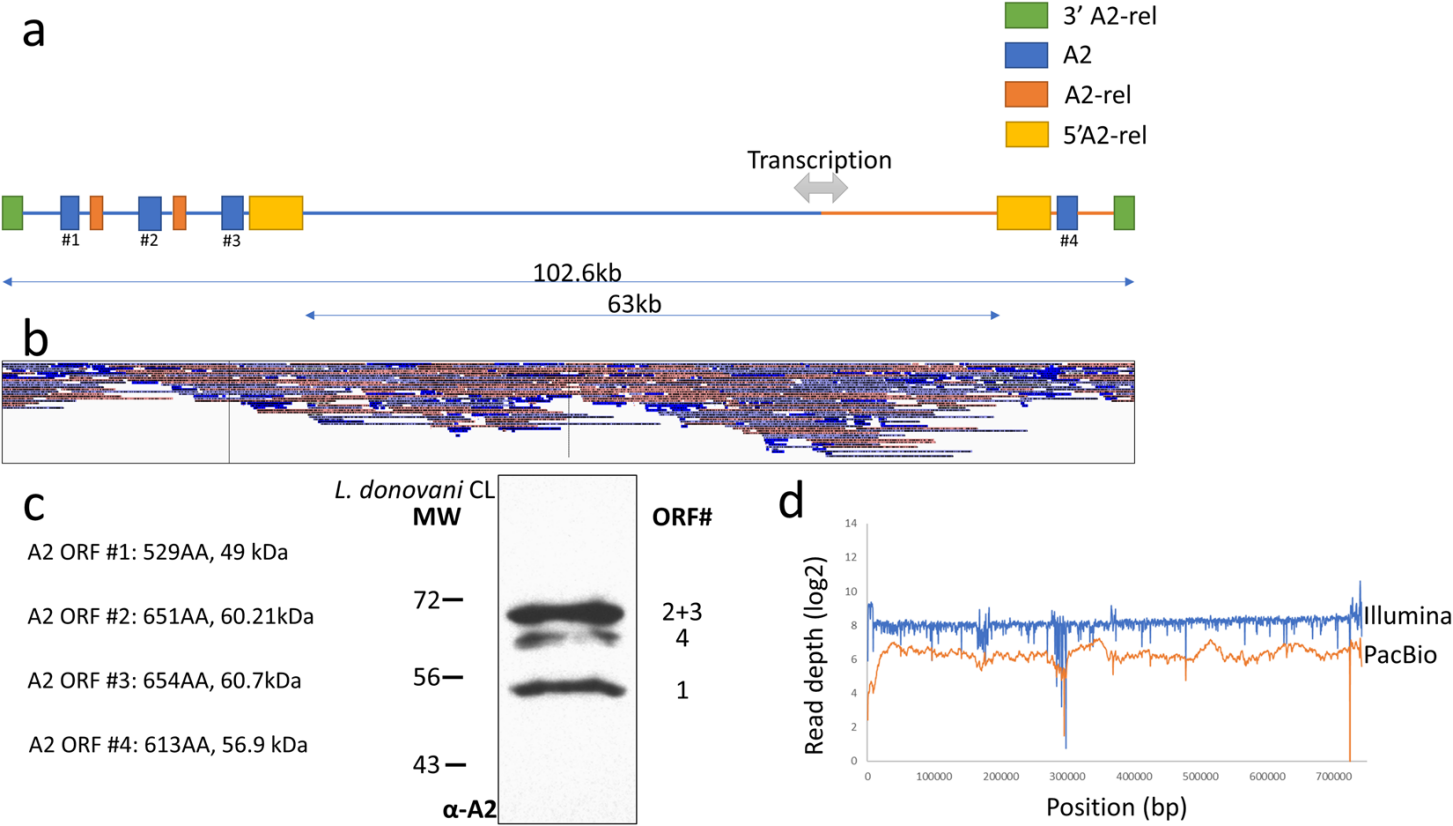
Organization of the 4 copies of the A2 gene on chromosome 22 in the attenuated cutaneous *L*. *donovani* strain. (**a**) Locations of the 4 A2 genes are shown in blue and numbered 1–4. Interspaced A2-rel genes are labeled in orange, 3′ A2-rel genes are labeled in green and 5′ A2-rel genes are labeled in yellow. A2-rel genes have no homology with A2 genes^15^. Transcription direction is shown according to strandedness: blue represents reverse strand direction of transcription, red represents forward strand transcription. The genes located in the 63 kb region between opposing A2 clusters are not depicted for clarity. (**b**) Alignment of the longest (~11 kb+) PacBio reads to the A2 clusters. Reads in the 5′ to 3′ direction labeled in red; reads in the 3′ to 5′ direction labeled in blue. (**c**) Western blot analysis of A2 proteins in the attenuated cutaneous *L*. *donovani* strain. The sizes of the A2 proteins are consistent with the ORFs and number of A2 genes identified in this assembly. (**d**) Coverage graph of chromosome 22 using Illumina (blue) and PacBio (orange) reads.

A2 is a major virulence factor required for *L*. *donovani* survival in visceral organs^22^. The A2 gene family cluster on chromosome 22 has recently been assembled for *L*. *infantum*^18^, however has not been for *L*. *donovani*. We therefore investigated whether the structure of this region could be determined with this revised assembly. It was advantageous that the attenuated cutaneous *L*. *donovani* strain used in this assembly has fewer copies of the A2 genes than other virulent strains of *L*. *donovani*^4^. As shown in Fig. 2a, the new assembly could read-through the entire cluster of highly repetitive A2 and flanking sequences and could position the A2 genes and interspersed flanking 3′ A2-rel and 5′ A2-rel genes. The A2 genes are contained in two opposite facing clusters on either side of a strand-switch locus consisting of one cluster of 3 copies and one cluster with a single A2 gene. The long sequence reads generated by the PacBio sequencing were crucial in generating the assembly of the A2 genes where reads of 11 kb and longer are shown spanning the repetitive cluster (Fig. 2b).

To generate supporting evidence for this A2 gene assembly, Western blot analysis of the A2 proteins from this strain was performed to compare the number and sizes of the A2 proteins with the predicted molecular weights from this assembly (ORFs; Supplementary Fig. S2). As shown in Fig. 2c, the apparent molecular weights from Western blotting correspond to the sizes predicted from the sequenced ORFs. The 3 bands on the Western blot are consistent with the molecular weights of the 4 gene products as the A2 gene copies 2 and 3 encode proteins of a similar size (Supplementary Fig. S2). This represents the first complete structure and sequence for A2 genes in *L*. *donovani*, a prototype virulence factor. The difficulty in assembling this complex region is demonstrated in Fig. 2d, where a deviation from the average read coverage can be seen around the 300,000 bp position, in and around the A2 cluster, due to difficulties in the aligner assigning a unique position to similar reads across a repetitive region.

**Figure 3:**
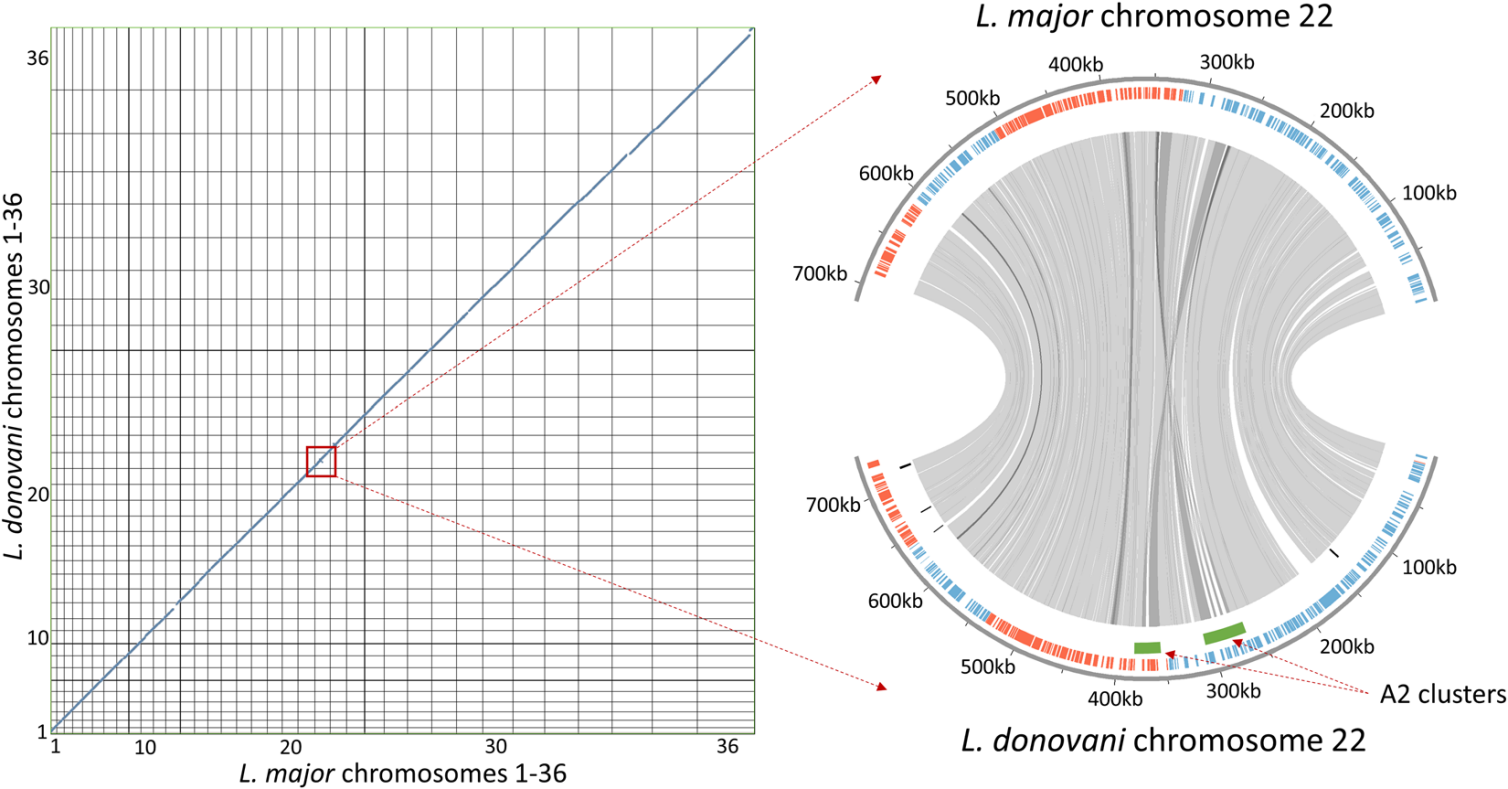
*L*. *donovani* maintains high levels of synteny with *L*. *major* including chromosome 22 where the A2 genes are located. Left: Dot plot of the coding DNA sequences of *L*. *major* compared to those of *L*. *donovani* generated from our assembly across the entire genome. Right: Synteny comparison of chromosome 22. The outer most circle represents the chromosomal location. The second circle is labelled with genes on the forward strand (blue) and genes on the reverse strand (red). The third circle represents genes that are only present in one of the two compared species. The inner association lines join syntenic genes between the two species.

Directly comparing synteny at the chromosomal level was not possible with the previous *L*. *donovani* assembly due to the heavy fragmentation of the genome. With the new *L*. *donovani* assembly, it was possible to accurately compare chromosome synteny between *L*. *donovani* and *L*. *major*. As shown in Fig. 3, the genome of *L*. *donovani*, exhibited a very high level of synteny with the *L*. *major*. Chromosome 22 was highlighted here because this is the location of the A2 genes that have become pseudogenes in *L*. *major* and have therefore diverged between these old-world species^15^. The level of synteny demonstrated here for chromosome 22 was maintained on all other chromosomes (Supplementary Fig. S3). These results indicate that evolution between cutaneous and visceral pathologies by different *Leishmania* species resulted largely from SNPs, pseudogenes and copy number variation and not from large changes such as chromosome rearrangements or complete gene deletions/insertions.

### Identification of new genes and improvements in annotations

**Figure 4:**
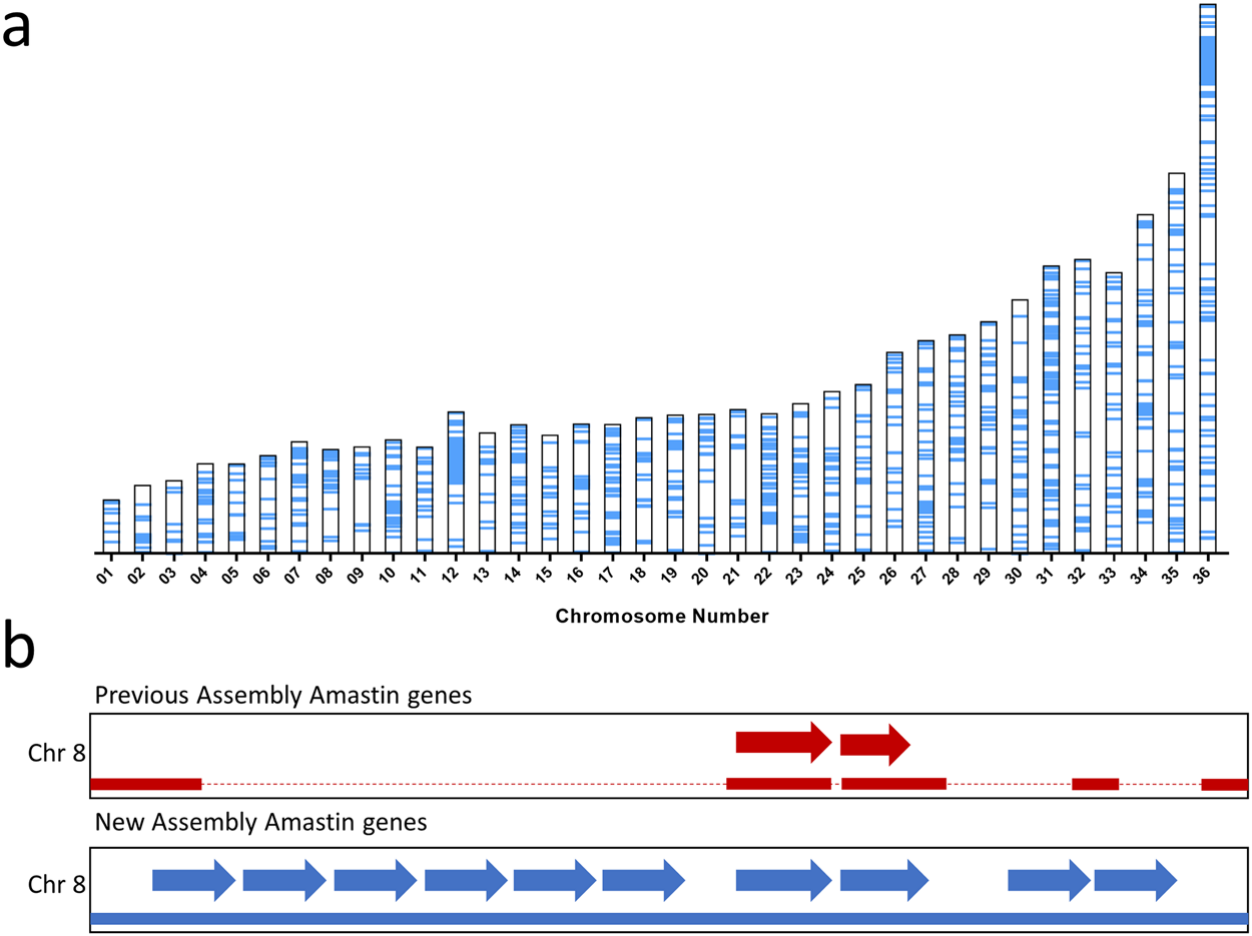
The new *L*. *donovani* genome assembly results in a significant change in gene annotations. (**a**) New or improved gene annotations are highlighted in Blue along the 36 chromosomes. Compared to the previous *L*. *donovani* reference assembly (ASM22713v2 from strain BPK282A1), there were 1,087 protein coding genes unannotated or differently annotated in the current assembly. Unannotated or differently annotated genes were obtained by removing all annotations generated from our assembly that shared 95% or greater similarity to those previously available^8^. (**b**) Expansion of the amastin gene cluster on chromosome 8. Top track contains the previously two known coding sequences aligned to the previous *L*. *donovani* reference assembly (ASM22713v2 from strain BPK282A1). Gaps in the previous assembly depicted as dotted lines. Bottom track contains 10 amastin genes identified in the updated assembly. One previously identified Amastin gene has been aligned, 1 has been expanded and 8 have been annotated *de novo*.

As this assembly was larger in terms of total number of bases covered and more contiguous due to the removal of sequence gaps, the impact this had on gene annotations was investigated. The genome from the new assembly was annotated using the Companion pipeline^23^ and the new and previous annotations (GenBank: GCF_000227135.1) were then aligned together and overlapping annotations were removed. Remarkably, close to 15% of the *L*. *donovani* protein coding genes had new or edited annotations as shown in Fig. 4a. Part of this increase in number of annotations resulted from the expansions of multi-copy gene families beyond the copy numbers in the previous annotation. An example is shown in Fig. 4b where there are 10 amastin genes identified in this new assembly compared to the previously identified 2. These results support the use of this assembly as the reference for bioinformatic analysis as it provides a more complete and accurate annotation of the *L*. *donovani* genome ORFs.

### Comparison of virulent and attenuated *L*. *donovani* parasites

**Figure 5:**
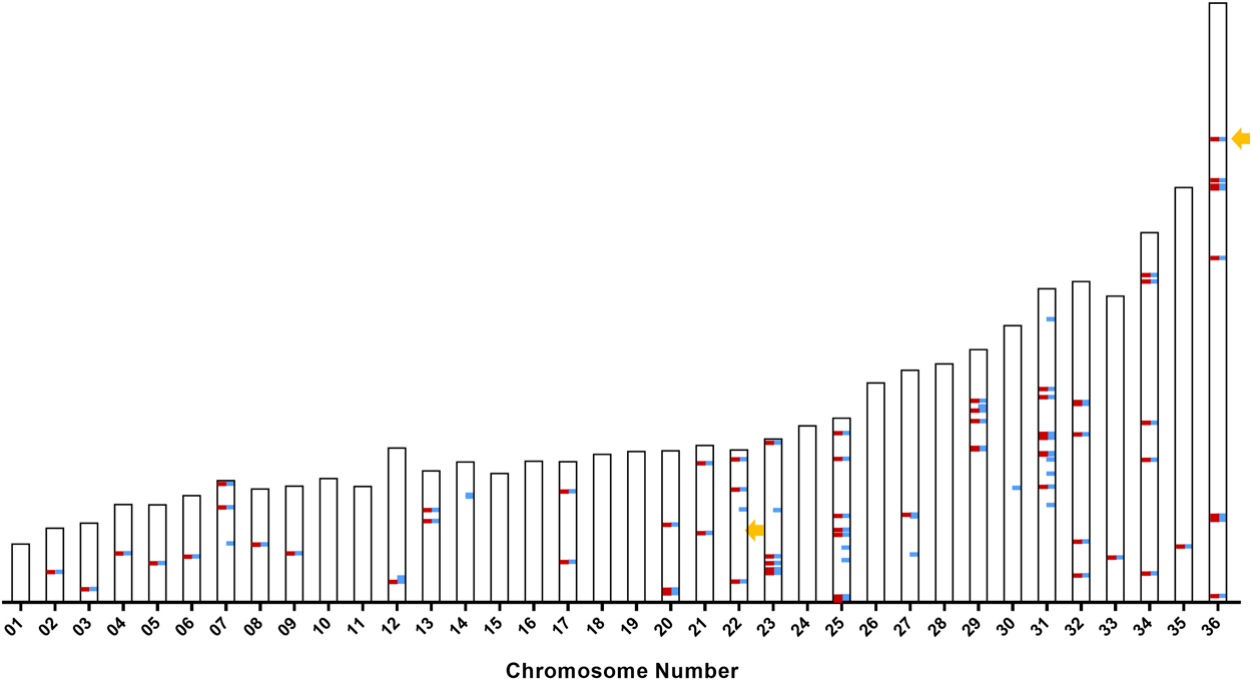
Verification of previously identified SNPs and location of new SNPs that differ between the virulent VL and attenuated CL strains of *L*. *donovani*. Chromosomal location of previously identified homozygous non-synonymous SNPs between the cutaneous and visceral disease derived *L*. *donovani* strains (Red)^4^ compared to the novel SNPs identified only in this study (Blue) (synonymous and heterozygous codon changes identified are not labeled). Note that all the previously identified SNPs were also identified, or confirmed, in this study. 70 SNPs were previously identified across 66 genes. The same 70 SNPs were identified in this study, with an additional 15 novel SNPs not previously seen specific to the cutaneous strain. Genomic locations of SNPs identified in the previous study were translated to new genomic coordinates based on the new assembly for consistency. Arrows in yellow highlight the position of the previously identified RagC SNP on chromosome 36 and the A2 copy number difference on chromosome 22.

As indicated above, there are 2 distinct strains of *L*. *donovani* in Sri Lanka where one is responsible for visceral leishmaniasis (VL) and the other for cutaneous leishmaniasis (CL)^4^. Subsequently, the CL strain was experimentally passaged through the visceral organs of BALB/c mice to select for a gain-of-function strain with increased virulence (IV strain) for survival in visceral organs where it was revealed through proteomic analysis that the resulting IV strain had an increase in stress response and antioxidant proteins^24^. Illumina whole genome sequencing and comparative genomic analysis of the VL, CL and IV strains was performed to identify SNPs associated with a change in virulence for survival in the visceral organs. As shown in Fig. 5, all 70 of the previously identified homozygous SNP differences between the VL and CL strains^4^ were confirmed in this new assembly and an additional 15 novel SNPs within protein coding genes were found using this complete assembly. In addition, there were 12 mutations associated with the IV strain with gain-of-function for increased survival in visceral organs not labeled in Fig. 5; four were heterozygous but with frequencies changing towards the VL genotype (IV → VL), four were heterozygous but present only in the IV strain and four were homozygous deletions in the IV strain. The newly identified differences between the VL and CL strains and the ones contributed from the IV strain are summarized in Table 2.

**Table 2:** Summary of novel mutations identified in this study.

Chr	Gene	Mutation	PROVEAN	Protein Name
7	*LdBPK_070700* LdCL_070011900	Ala282Val	−0.743	vacuolar-type Ca2 ± ATPase, putative
12	*LdBPK_120275* LdCL_120008300	Glu1157Asp	−0.258	Myotubularin-related protein, putative
14	LdCL_140017600	Ser2919fs	N/A	kinesin k39
14	*LdBPK_141190* LdCL_140017700	Glu1034Asp	−1.06	kinesin K39
22	*LdBPK_220840* LdCL_220015800	Pro219F/S	N/A	hypothetical protein
23	LdCL_230017500	INS:446Glu^a^	**−12.453**	sucrose hydrolase-like protein
25	*LdBPK_250620* LdCL_250011400	Ala969Glu	0.736	Raptor N-terminal CASPase like domain containing protein
25	*LdBPK_250790* LdCL_250013200	INS :110 Ala, Asn, Ser, Ala, Ala, Ala, Ala	N/A	hypothetical protein
27	*LdBPK_270830* LdCL_270014900	Ala1493Thr	−0.25	ATP-binding cassette protein subfamily A
29	LdCL_290028400	Thr208Ala	0.4	VIT family putative
30^1^	*LdBPK_301640* LdCL_300021700	Gln334STOP^a^	N/A	hypothetical protein
31	*LdBPK_311390* LdCL_310020800	STOP1486Leu,Ser,His	0	hypothetical protein
31	*LdBPK_311470* LdCL_310021600	Thr498Ala^a^	−0.15	hypothetical protein
31	*LdBPK_311470* LdCL_310021600	His497Arg^a^	0.942	hypothetical protein
31	*LdBPK_311470* LdCL_310021600	Gly380Asp^a^	−0.383	hypothetical protein
**IV** → **VL mutations**				
23	*LdBPK_230830* LdCL_230014900	Asp712Glu	−1.625	hypothetical protein, unknown function
31	*LdBPK_312870* LdCL_310037100	Met189Thr	**−4**	hypothetical protein, unknown function
31	*LdBPK_313290* LdCL_310041200	Val187Phe	−0.634	Hypothetical protein
34	*LdBPK_342210* LdCL_340029800	Thr116DEL	−1.098	hypothetical protein
**IV-Only mutations**				
14	*LdBPK_140470* LdCL_140010000	Gln89Lys	−0.044	cystathionine beta-lyase-like protein
31	*LdBPK_312810* LdCL_310036400	Cys173Phe	**−9.5**	regulator of chromosome condensation (RCC1) repeat, putative
32	*LdBPK_312770* LdCL_310035800	Gly667Ser	−1.292	hypothetical protein
32	*LdBPK_324000* LdCL_320046000	Val250Ile	0	hypothetical protein, unknown function
36	*LdBPK_361580* LdCL_360021300	Gene deletion	N/A	Serine/Threonine Kinase, putative
36	*LdBPK_361590* LdCL_360021400	Gene deletion	N/A	Serine/Threonine Kinase, putative
36	*LdBPK_361600* LdCL_360021500	Gene deletion	N/A	Engulfment and cell motility domain 2, putative
36	*LdBPK_361610* LdCL_360021600	Gene deletion	N/A	Predicted tripartite motif protein

All mutations are annotated using VL as the wild type amino acids and CL as the mutated amino acids. Genes with annotations in the previous assembly list the previous gene ID in italic, genes annotated only in this assembly list only one gene ID. The top segment lists fifteen attenuated cutaneous strain specific mutations identified in this study. Mutations marked with^a^ appear at 50% but also co-occur with gene duplication event and are therefore possibly homozygous on one copy. ‘INS’ denotes amino acid insertions, ‘F/S’ denotes frameshifts, ‘DEL’ denotes amino acid deletions. The middle segment lists four mutations where the gain-of-function IV strain’s genotype changed towards that of the visceral genotype. The bottom segment lists eight mutations present only in the gain-of-function IV strain and likely represents adaptations specific to the murine host. Calculated PROVEAN scores are shown in the fourth column, scores below the −2.5 threshold for deleterious mutations are highlighted in bold^25^.

**Figure 6:**
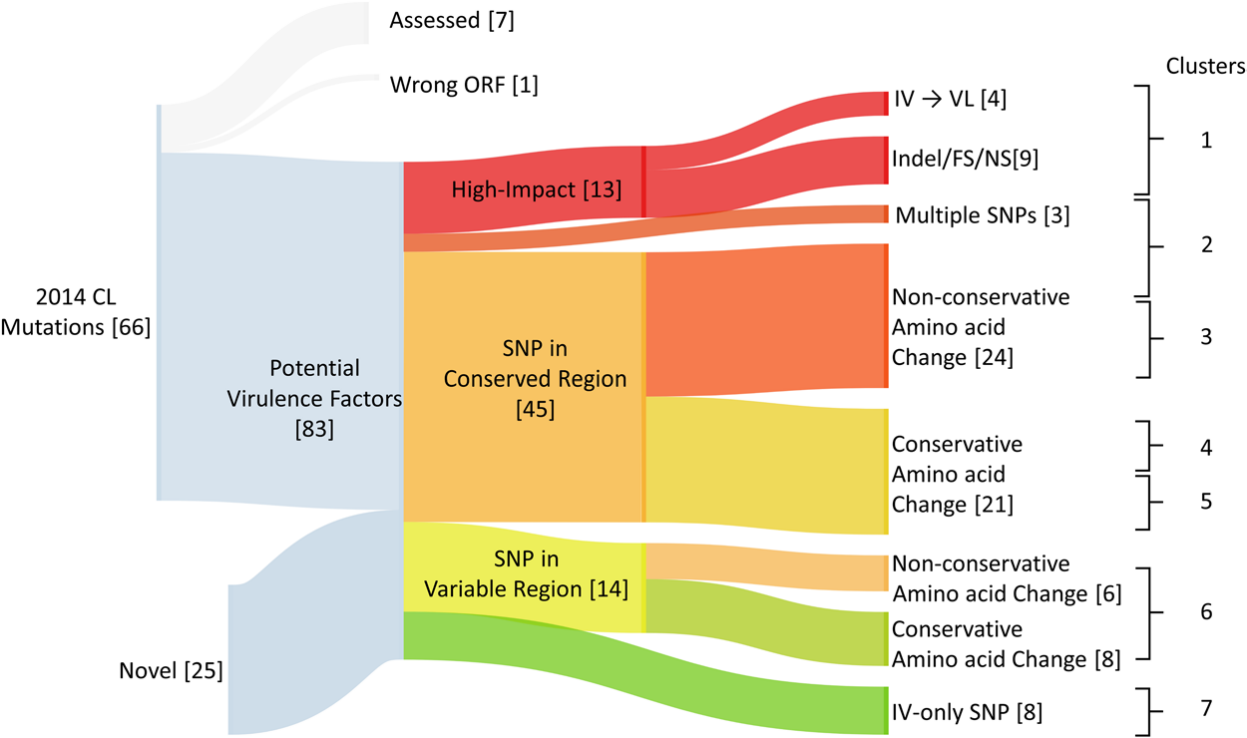
Summary of all genes with non-synonymous mutations between the cutaneous, visceral, and gain-of-function strains of *L*. *donovani*. All non-synonymous SNPs and Indels were classified as common to our previous study (2014 CL^4^) or identified in this study (Novel), as well as by their effect on amino acid changes from top to bottom, colored red to green in descending order of likelihood to affect the phenotype of the parasite. 66 genes were common to the previous data set. Of those genes, 7 were previously investigated^4^ and 1 was rejected due to an open reading frame misannotation. 25 genes were only listed in this study (Novel). Diagram created using SankeyMATIC (http://sankeymatic.com).

Combining data from the previous and current analysis, all the genes with genetic differences were organized into priority clusters based on the likelihood to affect protein function and phenotype (Fig. 6). A detailed list of the genes and cluster assignments is shown in Table 3. From the 66 genes containing 70 SNPs previously identified, 7 were previously experimentally assessed using gene replacement with a wildtype copy for virulence in visceral organs^4^ and one gene was identified as a misannotation and was therefore removed from the list. In decreasing order of priority, 13 genes in the highest impact cluster (red) were characterized as potentially having a major effect on protein function due to large amino acid changes or co-occurrence of mutations in both the VL and IV strains. SNPs in common between the IV and VL strains (4 IV → VL in the red cluster) indicate a selection associated with survival in visceral organs during the experimental passaging of the CL strain in mouse visceral organ^24^. Nine genes either with multiple co-occurring SNPs or non-conservative amino acid changes in conserved domains with a high score as assessed by PROVEAN software were placed in the second highest priority cluster. As detailed in methods, PROVEAN is a bioinformatic tool that classifies the significance of specific genetic mutations with respect to protein function^25^. Eighteen genes with non-conservative amino acid changes occurring in conserved domains but scored as unlikely to have a large effect on protein function by PROVEAN were placed in Cluster 3. Twenty-one genes with conservative amino acid changes in conserved domains were divided between Cluster 4 and Cluster 5 based on PROVEAN scores and 14 genes with mutations in domains with higher variability were placed in Cluster 6. Four mutations seen solely in the IV strain but not in the VL or CL strains are likely to be the result of random mutation or adaptation specific to the murine host were placed in Cluster 7.

**Table 3:** Summary of all genes containing mutations in the cutaneous isolates and classification into clusters.

*Cluster Number*	Cluster Mutation Type	New annotation	Equivalents (when available)
Cluster 1 (13)	Nonsense, Frameshift, Insertions, Deletions, IV to VL	LdCL_300021700	LdBPK_301640
		LdCL_310020800	LdBPK_311390
		LdCL_250013200	LdBPK_250790
		LdCL_310020100	LdBPK_311320
		LdCL_310022200	LdBPK_311510
		LdCL_080011700	LdBPK_080670
		LdCL_340029800	LdBPK_342210
		LdCL_230014900	LdBPK_230830
		LdCL_310037100	LdBPK_312870
		LdCL_220015800	—
		LdCL_140017600	—
		LdCL_230017500	—
		LdCL_310041200	LdBPK_313290
Cluster 2 (9)	Multiple SNPs in the same gene, Non-conservative amino acid change in conserved region with good PROVEAN score	LdCL_270015000	LdBPK_270840
		LdCL_290026900	LdBPK_292100
		LdCL_310021600	LdBPK_311470
		LdCL_310028800	LdBPK_312080
		LdCL_340046300	LdBPK_343690
		LdCL_290022800	LdBPK_291720
		LdCL_310024300	LdBPK_311710
		LdCL_360006000	LdBPK_360120
		LdCL_360062000	LdBPK_365480
Cluster 3 (18)	Non-conservative amino acid change in conserved region with poor PROVEAN score	LdCL_070018300	LdBPK_071330
		LdCL_320013800	LdBPK_320820
		LdCL_250016900	LdBPK_251150
		LdCL_220022000	LdBPK_221470
		LdCL_250015300	LdBPK_251000
		LdCL_040011100	LdBPK_040560
		LdCL_360016300	LdBPK_361120
		LdCL_200014300	LdBPK_200960
		LdCL_090011700	LdBPK_090660
		LdCL_130016200	LdBPK_131090
		LdCL_340009000	LdBPK_340390
		LdCL_130017800	LdBPK_131230
		LdCL_230009900	LdBPK_230440
		LdCL_220018100	LdBPK_221070
		LdCL_340044900	LdBPK_343550
		LdCL_290028400	—
		LdCL_250011400	LdBPK_250620
		LdCL_270014900	LdBPK_270830
Cluster 4 (8)	Conservative amino acid change in conserved region with good PROVEAN score	LdCL_350013100	LdBPK_350830
		LdCL_360052700	LdBPK_364550
		LdCL_230026600	LdBPK_231940
		LdCL_230009400	LdBPK_230400
		LdCL_320031100	LdBPK_322560
		LdCL_020008200	LdBPK_020280
		LdCL_310027700	LdBPK_311990
		LdCL_320031200	LdBPK_322570
Cluster 5 (13)	Conservative amino acid change in conserved region with poor PROVEAN score	LdCL_330011900	LdBPK_330640
		LdCL_170010200	LdBPK_170470
		LdCL_070011900	LdBPK_070700
		LdCL_210025000	LdBPK_211930
		LdCL_290022900	LdBPK_291730
		LdCL_200006300	LdBPK_200140
		LdCL_290029000	LdBPK_292290
		LdCL_030007500	LdBPK_030250
		LdCL_250006200	LdBPK_250110
		LdCL_360015800	LdBPK_361070
		LdCL_310023400	LdBPK_311630
		LdCL_360062700	LdBPK_365540
		LdCL_140017700	LdBPK_141190
Cluster 6 (14)	Non-conservative amino acid change in less conserved region, Conservative amino acid change in less conserved region	LdCL_340022100	LdBPK_341580
		LdCL_060011600	LdBPK_060650
		LdCL_210015400	LdBPK_211040
		LdCL_050010900	LdBPK_050580
		LdCL_230011600	LdBPK_230610
		LdCL_250024100	LdBPK_251840
		LdCL_070015100	LdBPK_071060
		LdCL_250005300	LdBPK_250040
		LdCL_310022100	LdBPK_311500
		LdCL_200006800	LdBPK_200200
		LdCL_250014400	LdBPK_250910
		LdCL_230010400	LdBPK_230500
		LdCL_120008300	LdBPK_120275
		LdCL_290028100	LdBPK_292210
Cluster 7 (4)	IV-only mutations	LdCL_140010000	LdBPK_140470
		LdCL_310035800	LdBPK_312770
		LdCL_310036400	LdBPK_312810
		LdCL_320046000	LdBPK_324000
		LdCL_360021300	LdBPK_361580
		LdCL_360021400	LdBPK_361590
		LdCL_360021500	LdBPK_361600
		LdCL_360021600	LdBPK_361610

Entries were not repeated in multiple lists.

Identified mutations were further classified into priority clusters for effect on protein function and future analysis for genes associated with survival in visceral organs. Mutations were prioritized by likelihood of contributing to visceral tissue tropism by severity of the coding change, accumulation of secondary mutations and conservation. Gene loci listed from the current assembly as well as previous ID numbers when available.

A 25 kb region on chromosome 36 containing 4 genes was found to be missing in the IV strain but present in the VL and CL strains. This deletion did not occur in a location previously identified on this chromosome where a fission can occur as seen in *L*. *alderi*^26^. Upon experimental verification, this deleted region was present in a subpopulation of the parental CL strain (Supplementary Fig. S4). The enrichment of this deletion in the IV strain could therefore be a consequence of selection in the mouse and likely to be unrelated to human visceral disease because this region is present in wild type or VL strains of *L*. *donovani* as well as *L*. *major* and therefore classified in cluster 7.

The classification of genetic differences in the CL, VL and IV genomes summarized in Fig. 6 and Table 3 represents a prioritization of genes to be empirically investigated for controlling the different phenotypes of these virulent and attenuated strains.

## Discussion

It has been possible to generate a complete genome assembly for *L*. *donovani* through combining second and third generation sequencing technologies, similarly to a recent resequencing of the *L*. *infantum* genome resulting in a complete assembly, highlighting the usefulness of PacBio sequencing in regards to *Leishmania* genomes^18^. This resulted in a more accurate annotation of the genome increasing the number of potential protein coding genes and identifying novel mutations/polymorphisms associated with virulence. It was remarkable that the present assembly resulted in annotation changes in close to 15% of the genome representing 1087 protein coding genes. Although 13 degenerate pseudogenes are identified in Supplementary Table S1 more do exist since our annotations derived from functional genes in *L*. *major* and therefore genes functional in other species were not identified. Through this updated genome annotation, additional SNPs have been identified including in genes potentially involved in visceral disease and several non-coding genes have been annotated allowing future *L*. *donovani* research beyond protein coding genes. It has also been possible to assemble known virulence factor gene families in *L*. *donovani* including the A2 and Amastin gene families. This version of the *L*. *donovani* genome assembly will significantly improve genomic, functional genomic and proteomic research outcomes and support the identification of drug targets and the development of vaccines. This assembly further provides a larger repertoire of target DNA sequences to identify diagnostic and prognostic disease progression markers. Given the recent interest in generating genetically modified live attenuated parasites as vaccine candidates^27^, a complete genome assembly will permit the verification that genetic modifications target intended genes with no off target mutations.

Supported by a *de novo* assembly, this study provides the first direct evidence for synteny between chromosomes in *L*. *donovani* and *L*. *major*, two old world parasite species with different pathologies and reservoirs. Previously, due to the large number of gaps in the *L*. *donovani* genome, the segments were aligned to a reference assembly assumed to be syntenic and only gene synteny was possible. In contrast, the contiguous assembly presented within used an entirely reference-free and by extension, bias-free generation process. This assembly can be used in future sequencing efforts aimed at comparing genes and synteny of genomes of other *Leishmania* species with *L*. *donovani*. The strong gene level synteny further highlights the major phenotypic effects of SNPs and indel mutations when comparing genomes from *L*. *donovani* strains causing visceral and cutaneous pathologies. As no major chromosomal rearrangements or deletions are apparent between phenotypically different *Leishmania* species as previously reported^2,4^, and including this study, suggesting that virulence and tropism can be acquired or lost through relatively small coding changes at the amino acid level such as SNPs, indels and frameshifts without the need for chromosomal scale events.

This study reports the complete A2 gene continuous sequence and an assembly of an entire A2 cluster including the A2-rel flanking genes in *L*. *donovani*. While the organization of this A2 gene cluster was previously theorized based on available sequences and Southern blot analysis, no sequencing technology could accurately read-through an entire cluster^4,28^ prior to third-generation sequencers. Interestingly, a similar organization of A2 and A2-rel flanking genes was obtained during the resequencing and assembly of the *L*. *infantum* genome^18^, further supporting the genomic arrangement of this important virulence cluster. The present assembly contains entire A2 ORFs that are consistent with the corresponding protein sizes determined by Western blot analysis and provides novel insight into this elusive virulence factor through identifying 2 amino acid insertions between the 10 amino acid repeats at geometric intervals as well as a defined C-terminal sequence (Supplementary Fig. S2). The deviations from the 10-amino acid repeat sequence could contribute to the proper folding and function of the A2 protein.

In an attempt to identify additional genes associated with survival in visceral organs, the attenuated cutaneous *L*. *donovani* strain was experimentally passaged continuously through the visceral organs of BALB/c mice over an 8 month period to generate a gain-of-function strain with increased survival in the liver and spleen and was termed the IV strain^24^. Sequence analysis of the IV strain in this study did not identify homozygous SNP differences with the parental cutaneous strain but did identify four heterozygous SNPs with the same sequence in the virulent *L*. *donovani* strains, classified as high impact in Fig. 6. The corresponding SNP-containing genes with unknown function are of high priority for future studies. Nevertheless, although the gain of function IV strain had significantly increased survival in visceral organs^24^, it was surprising that this strain did not have more genetic differences associated with the increased virulence. It is possible that the selection process for survival in the visceral organs of mice is different from that in humans.

The Illumina sequence analysis of the cutaneous (CL) and visceral (VL) disease associated strains using the complete assembly identified 15 novel homozygous SNPs beyond the previously identified 70 SNPs^4^(Fig. 5). One of these new SNPs was in the Raptor gene that is part of the highly conserved Target Of Rapamycin (TOR) signaling pathway^29^. There are three TOR gene homologs in the *Leishmania* genome^30^ revealing this pathway is conserved in kinetoplastids. Interestingly, the RagC GTPase which is a binding partner of Raptor in the TOR pathway is also mutated in the attenuated cutaneous *L*. *donovani* strain and restoration of the wildtype RagC GTPase increased virulence in visceral organs^4^. Considering that there are two mutated genes (RagC and Raptor) in the TOR pathway in the attenuated cutaneous *L*. *donovani* strain strongly highlights this pathway as playing a role in determining disease tropism and virulence.

As both HIVE and VarScan were used to identify SNPs and indels, we are confident that the expanded list of 83 variable genes shown in Fig. 6 contains most if not all the genes associated with visceral disease, with the exception of UTR mutations that may influence protein expression levels. Since this number of genes is relatively small, we are currently investigating all genes in clusters 1–4 with respect to their involvement in visceral organ virulence using CRISPR-Cas9 gene editing recently developed for use in *Leishmania*^31,32^. It is noteworthy that the correct selection of gRNA sequences for CRISPR-Cas9 gene editing requires a complete genome and accurate annotations for precise gene editing with no off-target mutations that is now possible with the complete assembly reported here.

## Methods

### Whole genome sequencing

#### DNA extraction

Leishmania DNA for both Illumina and PacBio sequencing was derived from the attenuated cutaneous strain of *L*. *donovani* from Sri Lanka^4^ that was passaged through mice to increase survival in visceral organs^24^. DNA was extracted following the previously described phenol-chloroform methods for isolation of Trypanosomatid genomic material^33^.

#### Illumina

Sequencing library preparation (Kapa HTP) and 250 nt paired-end sequencing (Illumina MiSeq) was performed using manufacturers’ protocols.

#### PacBio sequencing

A total of 9 sequencing cells were prepared. 7 cells were prepared using the DNA Template kit v2.0 (3–10 kb) with DNA/Polymerase Binding Kit P4 and 2 using the DNA Template Prep Kit 3.0 with DNA/Polymerase Binding Kit P5. The cells were sequenced on a PacBio II RS instrument with BaseCaller v1 protocol.

### Genome assembly

#### HGAP assembler

Raw reads from the 9 sequencing cells were loaded into the SMRT Analysis portal (Pacific Biosciences) in HD5 format. The Hierarchical Genome Assembly Process (HGAP) version 2 with Quiver polishing was chosen as version 3 is stated to improve speed at the detriment of assembly quality. Expected genome size was set to 36Mbp, minimum read length for pre-assembly was set to 500 bp and minimum read length for full assembly was set to 100 bp. Minimum Polymerase Read quality was set to 0.80, and the remainder of options remained at default settings.

#### Celera assembler

The PacBio corrected Reads (PBcR) module of the celera-assembler version 8.3 was used to assemble the long reads data^34^. The subreads were first extracted from the PacBio H5 files to FASTQ using bash5tools.py. The Bogart unitigger was used by specifying the “unitigger = bogart” option in the spec file. The consensus caller module was PBDAGCON. Due to the sequences originating from a non-clonal sample and the use of the DNA/Polymerase Binding kit P4 in some PacBio sequencing cells which produces lower quality data than P5 kits, error rate limits were relaxed for various variables, listed in the full spec file available in supplementary information (Supplementary Methods S1).

#### Canu assembler

The Canu v1.0 assembler is a modified version of the Celera Assembler designed to handle high noise data such as NanoPore and PacBio sequencing data. Canu has both the ability to assemble raw PacBio data by performing error correction using consensus sequence or assemble data in a hybrid mode where PacBio reads are pre-error corrected using short read Illumina data. In Raw mode, the trimmed PacBio reads were given to the assembler using default settings except for the expected genome size option which was set to 35Mbp using the option “genomeSize = 35 m”. In hybrid mode, the Illumina reads were first error corrected by internal consensus using Pollux^35^, the paired end reads were then merged together to form longer sequences with a high confidence core region using FLASh^36^, and used to correct the PacBio reads using Proovread^37^. The error-corrected PacBio data was then used by Canu to generate a draft assembly.

#### Pilon

The Pilon error correcting software was used to fix small errors present in the PacBio based assemblies using high depth and high accuracy Illumina data^38^. The entire MiSeq dataset in FASTQ format from the corresponding sample was aligned to the draft assembly using the Burrows-Wheeler Aligner (bwa) to generate SAM alignment files. Samtools was then used to convert and sort the files to a binary usable format as described in the Samtools section. This alignment was then passed to the Java Pilon executable for correction of small indels, SNPs, gap filling and assembly of unmapped reads using the command “java -Jar pilon.jar–genome [new-assembly.fasta] –frags [alignment.bam] –fix all,novel”.

#### GMCloser

GMcloser was used to merge the assemblies generated using the different assemblers^39^. Short read Illumina data was aligned to the contigs resulting from the different contigs from different assemblers with identical reads mapped to them were assumed to be part of the same chromosome. When a contig from one assembly encompassed a gap present in another assembly, the gap was filled with the missing information to generate a merged assembly with the least number of gaps. All the alignment and merging steps are handled internally to GMcloser using the command “gmcloser -t [assembly1.fasta] -q [assembly2.fasta] -r miseq_R1.fastq miseq_r2.fastq -et”.

#### IGV

The Broad Institute Integrative Genome Viewer^40,41^ was used to perform quality control on assemblies and manually inspect fragments in order to close gaps. The Pilon tools was used with the “-fix novel” option which assembled short contigs from unmapped data. The fragments were then placed on the appropriate likely chromosomes based on gene annotations and submitted to another round of gap filling using Pilon and GMCloser to find reads supporting this placement or were removed if no reads supported the join.

### Annotations

#### Companion

The Companion webtool (https://companion.sanger.ac.uk/) was used to annotate genes on the assembly contigs and refine the assembly^23^. The closest available reference organism was chosen (*L*. *major)* with the following options: contiguate pseudochromosomes, align reference proteins to target sequence, perform pseudogene detection, use RATT Species transfer type, and the *L*. *donovani* taxon ID. Additional *L*. *donovani* and *L*. *major* genes not automatically transferred were manually verified and appended if necessary. An additional 3 genes were manually added from a search of all ribosomal protein transcripts in trypanosomes. The snRNAs U1 through U6, ribosomal RNAs and the spliced leader RNA were manually annotated as necessary from the sequences available for *L*. *major* on TriTrypDB^42^. Sequences for H/ACA and C/D box snoRNA were manually mapped using published *L*. *major* snoRNA research^43^.

#### Galaxy

The Galaxy webtool (https://usegalaxy.org/)^44^ was used to perform file conversions and data extraction such as moving a chromosome’s FASTA sequence from one assembly to another.

#### Identification of new genes

Genomic annotations from the Companion Pipeline were downloaded in General Feature Format (GFF) and gene annotations were extracted using the Galaxy tool “Extract features” set to look for the “CDS” keyword in column #3 of the GFF file. Known coding regions from the reference *L*. *donovani* strain BPK282A1, assembly ASM22713v2 were downloaded from GenBank and aligned to our improved assembly in BED format. Bedtools intersect intervals through Galaxy^45,46^ was used to identify annotations that were unique to our annotations or were not at least 95% covered previously using settings “-wa -f 0.95 -v -r”.

#### Synteny

The online SynMap2 software^47^ was used to generate the synteny dotplot across the entire genome using annotations from *L*. *major* and the annotations generated by Companion in this study. The chromosome to chromosome circular charts were generated by Companion as part of the annotation process.

### Comparison of visceral (VL), cutaneous (CL) and increased virulence (IV) *L*. *donovani* strains

#### BWA

The Burrows-Wheeler Aligner (BWA) was used to process the FASTQ Illumina sequencing files obtained from Genome Quebec. The maximal exact match algorithm was used in paired-end mode using the command “bwa mem” and providing the matched pair read files and reference sequence as arguments in order to generate a SAM format alignment file of the reads on the reference^48^.

#### Samtools

The samtools package was used for file manipulations and conversions^49^. The commands “samtools view -b” was used to convert the BWA generated SAM file to the binary alignment BAM format. The file was then sorted by alignment location for compatibility with downstream analysis software using “samtools sort -@ 30 -o [output.file]”. The alignment files where then prepared for analysis using the mpileup modules which tabulates the base distribution at every position using the command “samtools mpileup -B -f [reference assembly] [strain specific position sorted BAM file] > [output.file]”.

#### VarScan

The VarScan v2^50^ mutation caller was used to generate a list of mutations in Variant Call Format (VCF) using the mpileup file generated by samtools as described above using the command “java -jar VarScan.jar mpileup2snp –output-vcf 1 [mpileup.file] > [output.VCF]”. We also used VarScan to generate indel locations based on the same mpileup file using the command “java -jar VarScan.jar mpileup2indel –output-vcf 1 [mpileup.file] > [output.vcf]”.

#### SnpEff

To filter the VCF files generated by VarScan to a list of non-synonymous SNP, we used the SnpEff software^51^. The oriented and annotated assembly was downloaded from the Companion tool as described above along with the gene annotation file in GFF format containing the names, locations and amino acid sequences of identified genes. This GFF file was used to build a SnpEff database using the SnpEff.jar command “build” with argument “-gff” after installing the genome and GFF file in the appropriate locations according to the software instructions.

The SnpEff software was then used to annotate the 10^th^ column of the VCF file with mutation effect codes. All the mutations were then examined manually for accuracy using the Integrative Genomics Viewer (IGV) with all raw Illumina data loaded.

#### Classification

Non-synonymous mutations were clustered according to the mutation effect in order to prioritize further gene function studies. Each cluster was further broken down based on the mutation’s PROVEAN score^25^. The PROVEAN software was designed to predict the magnitude of a mutation’s impact on protein function. To generate PROVEAN scores, we retrieved homologous sequences from other *Leishmania* species and kinetoplasts and generated a multiple sequence alignment (MSA). The MSA was then passed to the PROVEAN software which scored each SNP based on the alignment. We used PROVEAN scores below a threshold of −2.5 as an indication a SNP is likely to affect protein function. Cluster assignments were as follows:

1. Mutations likely to have the largest impact on protein function were included; non-sense, frameshift and amino acid insertion/deletions as well as all SNPs in the gain-of-function IV strain that were the same in the virulent visceral strain (VL) allele, indicating a selection pressure on those genes for visceral organ survival.
2. Genes in with multiple SNPs and genes where non-conservative mutations occurred in highly conserved *Leishmania/Kinetoplastida* regions.
3. Due to the high number of genes in cluster 2, split off poor PROVEAN scoring genes.
4. Cluster 4 comprised genes with conservative amino acid changes but occurring in *Leishmania/Kinetoplastida* conserved regions.
5. Due to the high number of genes in cluster 4, split off poor PROVEAN scoring genes.
6. Conservative and non-conservative amino acid changes in less conserved regions.
7. Changes present only in the gain of function IV strain. This cluster was considered low probability as it likely contains either random mutations or adaptations specific to survival in the murine host.

### HIVE

HIVE^52^ was used to perform differential profiling of genomes from visceral (VL), gain of function increased virulence (IV), and (cutaneous) CL strains.

1. Reads from all the samples were aligned to the assembly of the genome using HIVE-hexagon^53^ parametrized for parasitic eukaryotic species and specifically adjusted to work with *Leishmania* analysis as demonstrated in previous studies^27^.
2. Coverage and variant calling analysis was performed using HIVE-heptagon^54^ to produce variant call frequencies and coverages for every genomic position.
3. HIVE differential profiler^52^ was used to analyze relative differences in SNP calls and variant coverages for multiple samples.

### A2 Immunoblotting

A2 Immunoblotting was performed as previously described^16^. Briefly, 1 × 10^7^ cutaneous CL strain promastigotes were collected at mid log-phase and resuspended in 1 mL fresh medium. The cells were then heat-shocked for 4 h at 40 °C to induce A2 protein expression, washed, lysed in SDS-PAGE loading buffer and loaded on a 10% (w/v) acrylamide gel. The proteins were transferred to nitrocellulose at 25 V overnight at 4 °C. The membrane was blocked for 1 h in 10% (w/v) skim milk powder dissolved in PBS with 0.1% (v/v) Tween-20. The membrane was then incubated for 1 h at RT with a 1:10,000 dilution of C9 Ascites fluid (anti-A2 Mab) in blocking solution followed by 6 × 5 min washes in PBS-T. Secondary HRP labeled anti-mouse IgG antibody (Thermo Fisher Scientific) was incubated at 1:10,000 in blocking buffer for 1 h at RT followed by 6 × 5 min washes in PBS-T. The membrane was incubated in ECL reagent (Zm Tech) for 1 min at RT before being exposed to x-ray film (Denville Scientific). Film images were captured using a Gel-Doc XR documentation system with Quantity One software (BioRad Laboratories).

### Accession codes

 GenBank BioProject PRJNA450813.

## Electronic supplementary material

Supplementary information

## Data Availability

All data used in this study have been deposited online with GenBank. Raw PacBio reads for the IV strain, Illumina MiSeq reads for the CL, VL and IV strains, the new genome assembly and the annotations generated in this study can be found under the PRJNA450813 BioProject accession number.

## References

[1] Alvar J (2012). Leishmaniasis worldwide and global estimates of its incidence. PLoS One.

[2] Peacock CS (2007). Comparative genomic analysis of three Leishmania species that cause diverse human disease. Nat. Genet..

[3] Zhang WW, Matlashewski G (2010). Screening Leishmania donovani-specific genes required for visceral infection. Mol. Microbiol..

[4] Zhang WW (2014). Genetic Analysis of Leishmania donovani Tropism Using a Naturally Attenuated Cutaneous Strain. PLoS Pathog..

[5] Goodwin S, McPherson JD, McCombie WR (2016). Coming of age: Ten years of next-generation sequencing technologies. Nat. Rev. Genet..

[6] Schneider VA (2017). Evaluation of GRCh38 and de novo haploid genome assemblies demonstrates the enduring quality of the reference assembly. Genome Res..

[7] Grisard EC (2014). Trypanosoma cruzi Clone Dm28c Draft Genome Sequence. Genome Announc..

[8] Downing T (2011). Whole genome sequencing of multiple Leishmania donovani clinical isolates provides insights into population structure and mechanisms of drug resistance. Genome Res..

[9] Ivens AC (2005). The genome of the kinetoplastid parasite, Leishmania major. Science (80-.)..

[10] Rogers MB (2011). Chromosome and gene copy number variation allow major structural change between species and strains of Leishmania. Genome Res..

[11] Alonso, G., Rastrojo, A., López-Pérez, S., Requena, J. M. & Aguado, B. Resequencing and assembly of seven complex loci to improve the Leishmania major (Friedlin strain) reference genome. *Parasites and Vectors* **9** (2016). 10.1186/s13071-016-1329-4 PMC4746890 26857920

[12] Mikheyev AS, Tin MMY (2014). A first look at the Oxford Nanopore MinION sequencer. Mol. Ecol. Resour..

[13] Rhoads A, Au KF (2015). PacBio Sequencing and Its Applications. Genomics, Proteomics Bioinforma..

[14] Zhang W-W, Matlashewski G (1997). Loss of virulence in Leishmania donovani deficient in an amastigote-specific protein, A2. Proc. Natl. Acad. Sci..

[15] Zhang WW (2003). Comparison of the A2 gene locus in Leishmania donovani and Leishmania major and its control over cutaneous infection. J. Biol. Chem..

[16] McCall LI, Matlashewski G (2010). Localization and induction of the A2 virulence factor in Leishmania: Evidence that A2 is a stress response protein. Mol. Microbiol..

[17] McCall LI, Matlashewski G (2012). Involvement of the Leishmania donovani virulence factor A2 in protection against heat and oxidative stress. Exp. Parasitol..

[18] González-De La Fuente, S. *et al*. Resequencing of the Leishmania infantum (strain JPCM5) genome and de novo assembly into 36 contigs. *Sci*. *Rep*. **7**, (2017). 10.1038/s41598-017-18374-y PMC5741766 29273719

[19] Karunaweera ND, Pratlong F, Siriwardane HVYD, Ihalamulla RL, Dedet JP (2003). Sri Lankan cutaneous leishmaniasis is caused by Leishmania donovani zymodeme MON-37. Trans. R. Soc. Trop. Med. Hyg..

[20] Ranasinghe S (2012). *Leishmania donovani* zymodeme MON-37 isolated from an autochthonous visceral leishmaniasis patient in Sri Lanka. Pathog. Glob. Health.

[21] Singh N, Chikara S, Sundar S (2013). SOLiD^TM^ Sequencing of Genomes of Clinical Isolates of Leishmania donovani from India Confirm Leptomonas Co-Infection and Raise Some Key Questions. PLoS One.

[22] McCall Laura-Isobel, Zhang Wen-Wei, Matlashewski Greg (2013). Determinants for the Development of Visceral Leishmaniasis Disease. PLoS Pathogens.

[23] Steinbiss S (2016). Companion: a web server for annotation and analysis of parasite genomes. Nucleic Acids Res..

[24] McCall LI (2015). Adaptation of leishmania donovani to cutaneous and visceral environments: *In vivo* selection and proteomic analysis. J. Proteome Res..

[25] Choi Y, Chan AP (2015). PROVEAN web server: A tool to predict the functional effect of amino acid substitutions and indels. Bioinformatics.

[26] Coughlan, S. *et al*. The genome of Leishmania adleri from a mammalian host highlights chromosome fission in Sauroleishmania. *Sci*. *Rep*. **7** (2017). 10.1038/srep43747 PMC5335649 28256610

[27] Gannavaram, S. *et al*. Whole genome sequencing of live attenuated Leishmania donovani parasites reveals novel biomarkers of attenuation and enables product characterization. *Sci*. *Rep*. **7** (2017). 10.1038/s41598-017-05088-4 PMC5498541 28680050

[28] Zhang WW, Matlashewski G (2001). Characterization of the A2-A2rel gene cluster in Leishmania donovani: Involvement of A2 in visceralization during infection. Mol. Microbiol..

[29] Sancak Y (2008). The rag GTPases bind raptor and mediate amino acid signaling to mTORC1. Science (80-.)..

[30] Madeira da Silva L, Beverley SM (2010). Expansion of the target of rapamycin (TOR) kinase family and function in Leishmania shows that TOR3 is required for acidocalcisome biogenesis and animal infectivity. Proc. Natl. Acad. Sci..

[31] Zhang WW, Matlashewski G (2015). CRISPR-Cas9-mediated genome editing in Leishmania donovani. MBio.

[32] Zhang W-W, Lypaczewski P, Matlashewski G (2017). Optimized CRISPR-Cas9 Genome Editing for Leishmania and Its Use To Target a Multigene Family, Induce Chromosomal Translocation, and Study DNA Break Repair Mechanisms. mSphere.

[33] Medina-Acosta E, Cross GAM (1993). Rapid isolation of DNA from trypanosomatid protozoa using a simple ‘mini-prep’ procedure. Mol. Biochem. Parasitol..

[34] Berlin K (2015). Assembling large genomes with single-molecule sequencing and locality-sensitive hashing. Nat. Biotechnol..

[35] Marinier, E., Brown, D. G. & McConkey, B. J. Pollux: Platform independent error correction of single and mixed genomes. *BMC Bioinformatics* **16**, (2015). 10.1186/s12859-014-0435-6 PMC4307147 25592313

[36] Magoč T, Salzberg SL (2011). FLASH: Fast length adjustment of short reads to improve genome assemblies. Bioinformatics.

[37] Hackl T, Hedrich R, Schultz J, Förster F (2014). Proovread: Large-scale high-accuracy PacBio correction through iterative short read consensus. Bioinformatics.

[38] Walker Bruce J., Abeel Thomas, Shea Terrance, Priest Margaret, Abouelliel Amr, Sakthikumar Sharadha, Cuomo Christina A., Zeng Qiandong, Wortman Jennifer, Young Sarah K., Earl Ashlee M. (2014). Pilon: An Integrated Tool for Comprehensive Microbial Variant Detection and Genome Assembly Improvement. PLoS ONE.

[39] Kosugi S, Hirakawa H, Tabata S (2015). GMcloser: Closing gaps in assemblies accurately with a likelihood-based selection of contig or long-read alignments. Bioinformatics.

[40] Robinson JT (2011). Integrative genomics viewer. Nature Biotechnology.

[41] Thorvaldsdóttir H, Robinson JT, Mesirov JP (2013). Integrative Genomics Viewer (IGV): High-performance genomics data visualization and exploration. Brief. Bioinform..

[42] Aslett M, Aurrecoechea C, Berriman M, Al. E (2010). TriTrypDB: a functional genomic resource for the Trypanosomatidae. Nucleic Acids Res..

[43] Eliaz D (2015). Genome-wide analysis of small nucleolar RNAs of leishmania major reveals a rich repertoire of RNAs involved in modification and processing of rRNA. RNA Biol..

[44] Afgan E (2016). The Galaxy platform for accessible, reproducible and collaborative biomedical analyses: 2016 update. Nucleic Acids Res..

[45] Quinlan AR, Hall IM (2010). BEDTools: A flexible suite of utilities for comparing genomic features. Bioinformatics.

[46] Gruening, B. A. Galaxy wrapper (2014).

[47] Haug-Baltzell A, Stephens SA, Davey S, Scheidegger CE, Lyons E (2017). SynMap2 and SynMap3D: Web-based whole-genome synteny browsers. Bioinformatics.

[48] Li, H. Aligning sequence reads, clone sequences and assembly contigs with BWA-MEM. **arXiv:1303** (2013).

[49] Li H (2009). The Sequence Alignment/Map format and SAMtools. Bioinformatics.

[50] Koboldt DC (2012). VarScan 2: Somatic mutation and copy number alteration discovery in cancer by exome sequencing. Genome Res..

[51] Cingolani P (2012). A program for annotating and predicting the effects of single nucleotide polymorphisms, SnpEff. Fly (Austin)..

[52] Simonyan V, Mazumder R (2014). High-performance integrated virtual environment (hive) tools and applications for big data analysis. Genes (Basel)..

[53] Santana-Quintero Luis, Dingerdissen Hayley, Thierry-Mieg Jean, Mazumder Raja, Simonyan Vahan (2014). HIVE-Hexagon: High-Performance, Parallelized Sequence Alignment for Next-Generation Sequencing Data Analysis. PLoS ONE.

[54] Simonyan V (2017). HIVE-heptagon: A sensible variant-calling algorithm with post-alignment quality controls. Genomics.

