# Supplementary material for: A complete Leishmania donovani reference genome identifies novel genetic variations associated with virulence
Source: Sci Rep. 2018 Nov 8;8:16549. doi: 10.1038/s41598-018-34812-x (PMC6224596; doi:10.1038/s41598-018-34812-x)
Supplement: Supplementary file 1 — Supplementary information [file 41598_2018_34812_MOESM1_ESM.pdf]

A complete *Leishmania donovani* reference genome identifies novel genetic variations associated with virulence.

Patrick Lypaczewski[1], Johanna Hoshizaki[1], Wen-Wei Zhang[1], Laura-Isobel McCall[1,2], John Torcivia-Rodriguez[3], Vahan Simonyan[3], Amanpreet Kaur [4,5], Ken Dewar[4,5], Greg Matlashewski[1]*

Supplementary Table S1. List of *L. donovani* pseudogenes derived from *L. major*

Chromosome	Start	Stop	Strand	Gene ID	Internal Stop	Frameshift	L. Major origin
LdCL_06	518294	520041	+	LdCL_060018900		XXXX	LmjF.22.0010
LdCL_08	550785	551865	+	LdCL_080018700		XXXXX	LmjF.08.1265
LdCL_10	79199	79729	+	LdCL_100006900	XX	XX	LmjF.10.0185
LdCL_12	603220	604085	+	LdCL_120018300		XXXXXX	LmjF.12.0880
LdCL_16	316885	317791	-	LdCL_160014100	XXXX	XXXX	LmjF.16.0880
LdCL_26	580729	582519	+	LdCL_260021400	XXXX	XXX	LmjF.26.1590
LdCL_27	527810	529301	-	LdCL_270017800	XXXX	XXXXXX	LmjF.14.0180
LdCL_27	732393	732680	-	LdCL_270023400*	X		LmjF.27.1740
LdCL_31	1179486	1180414	-	LdCL_310031700	XXXXX	XXX	LmjF.31.2310
LdCL_31	1334630	1335706	-	LdCL_310035400	XXX	XX	LmjF.29.1570
LdCL_32	784327	785612	-	LdCL_320026000	XX	X	LmjF.32.1950
LdCL_33	702441	703568	+	LdCL_330023800	XXXXX	XXXXX	LmjF.32.2640
LdCL_35	1479577	1480153	-	LdCL_350044600	XX	XX	LmjF.35.3910

13 pseudogene annotations in *L. donovani* originated from 13 intact functional genes from *L. major* in the current assembly. The 13 pseudogenes were common across all 3 isolates sequences in this study. The locations of the pseudogenes are given in chromosomal position from start to stop. Strand indicates direction of transcription. Each stop codon and frameshift are marked with an X *L. major* origin denotes the equivalent functional gene ID in *L. major*.

*One gene contains only one stop codon; this gene however is also missing a start codon.

Supplementary Figure S1. Coverage graphs of the new *L. donovani* assembly

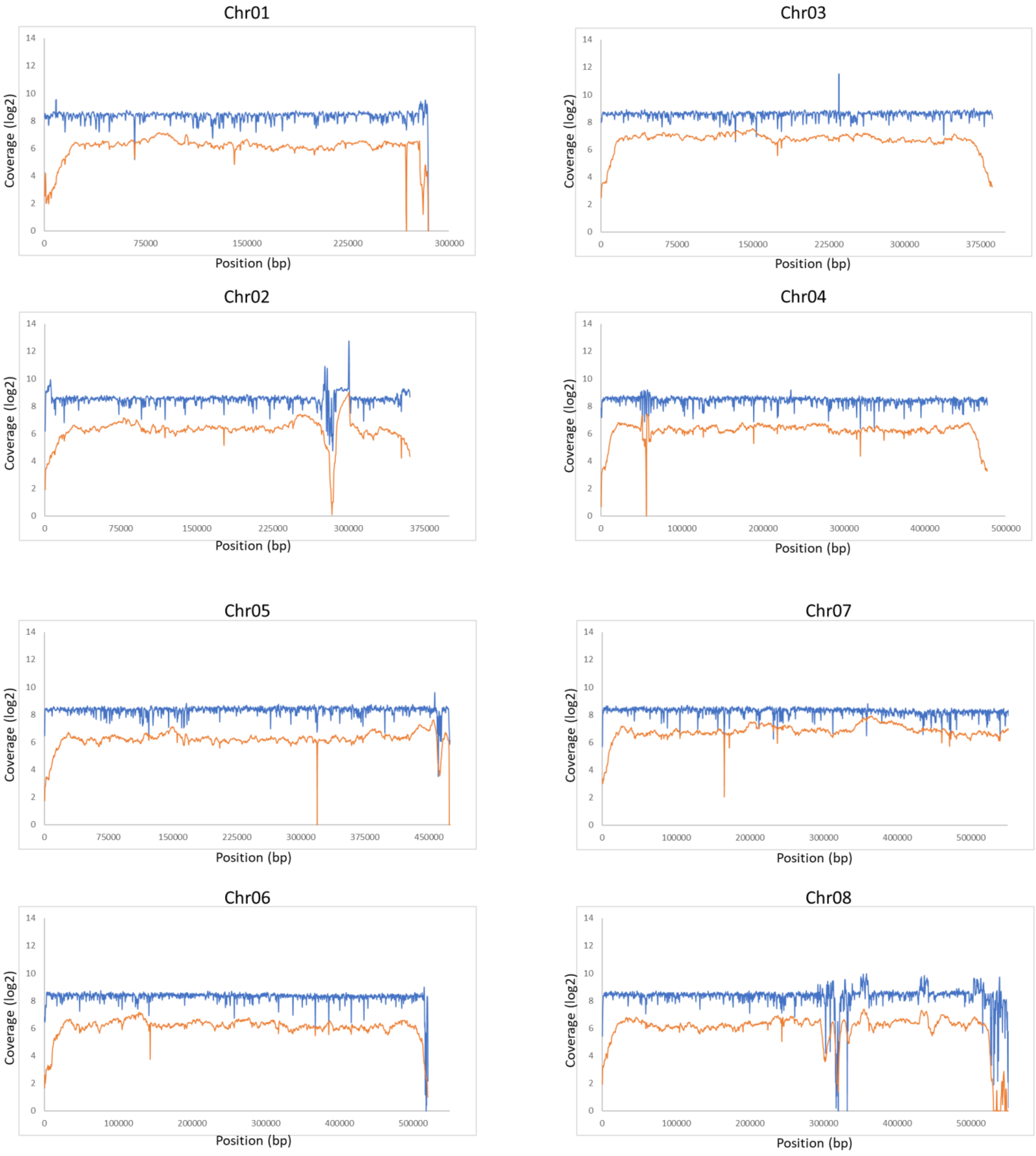

Chr09

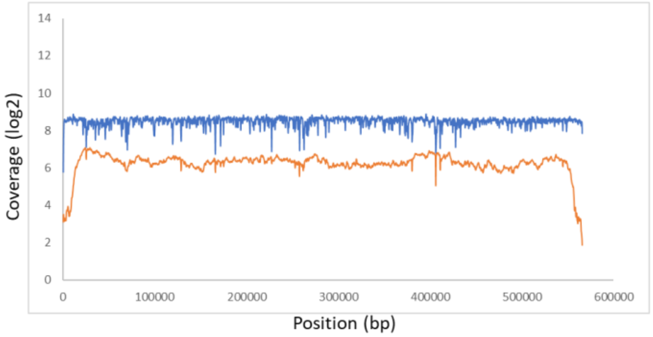

Chr11

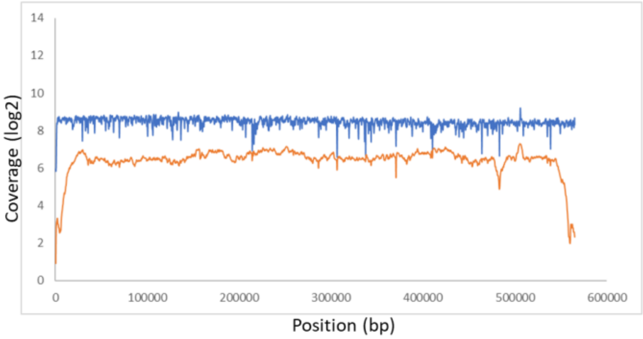

Chr10

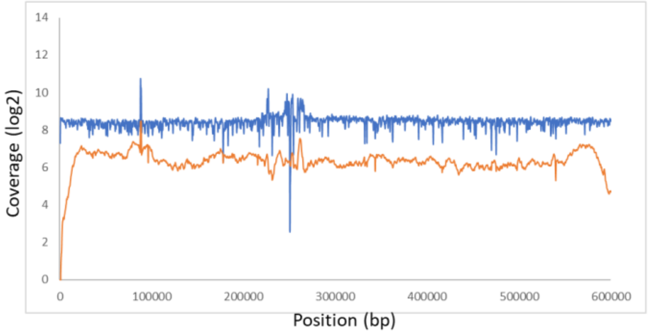

Chr12

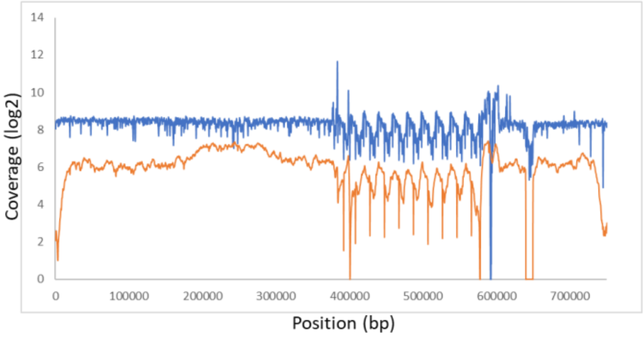

Chr13

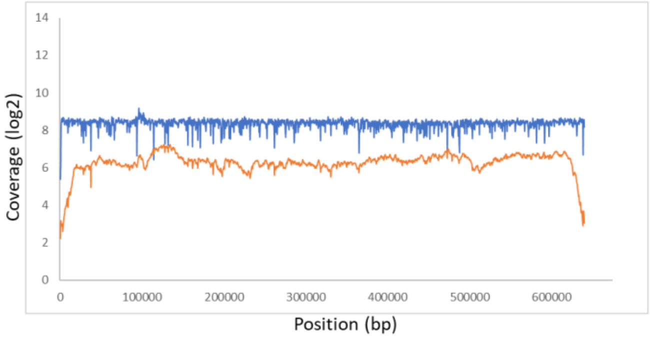

Chr15

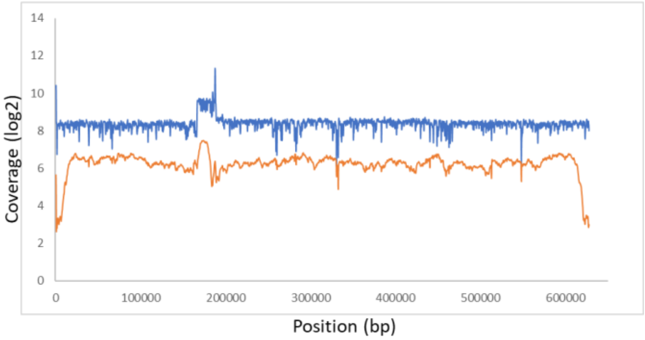

Chr14

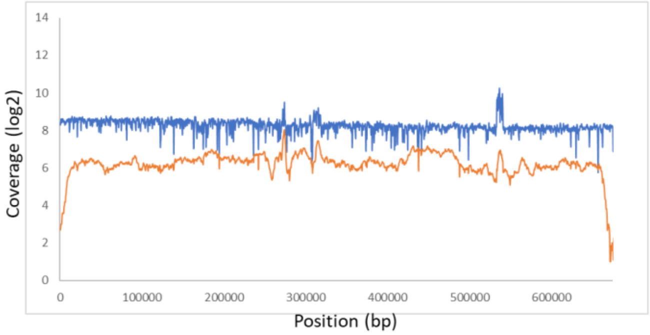

Chr16

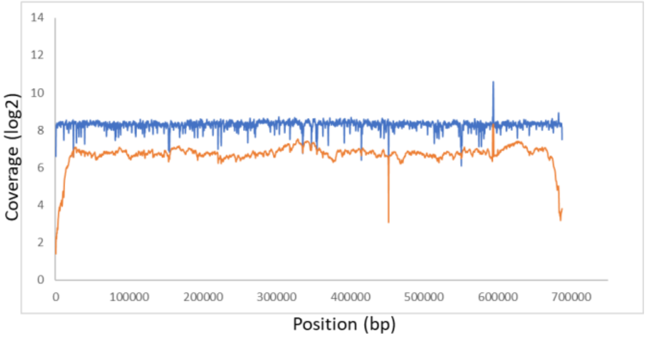

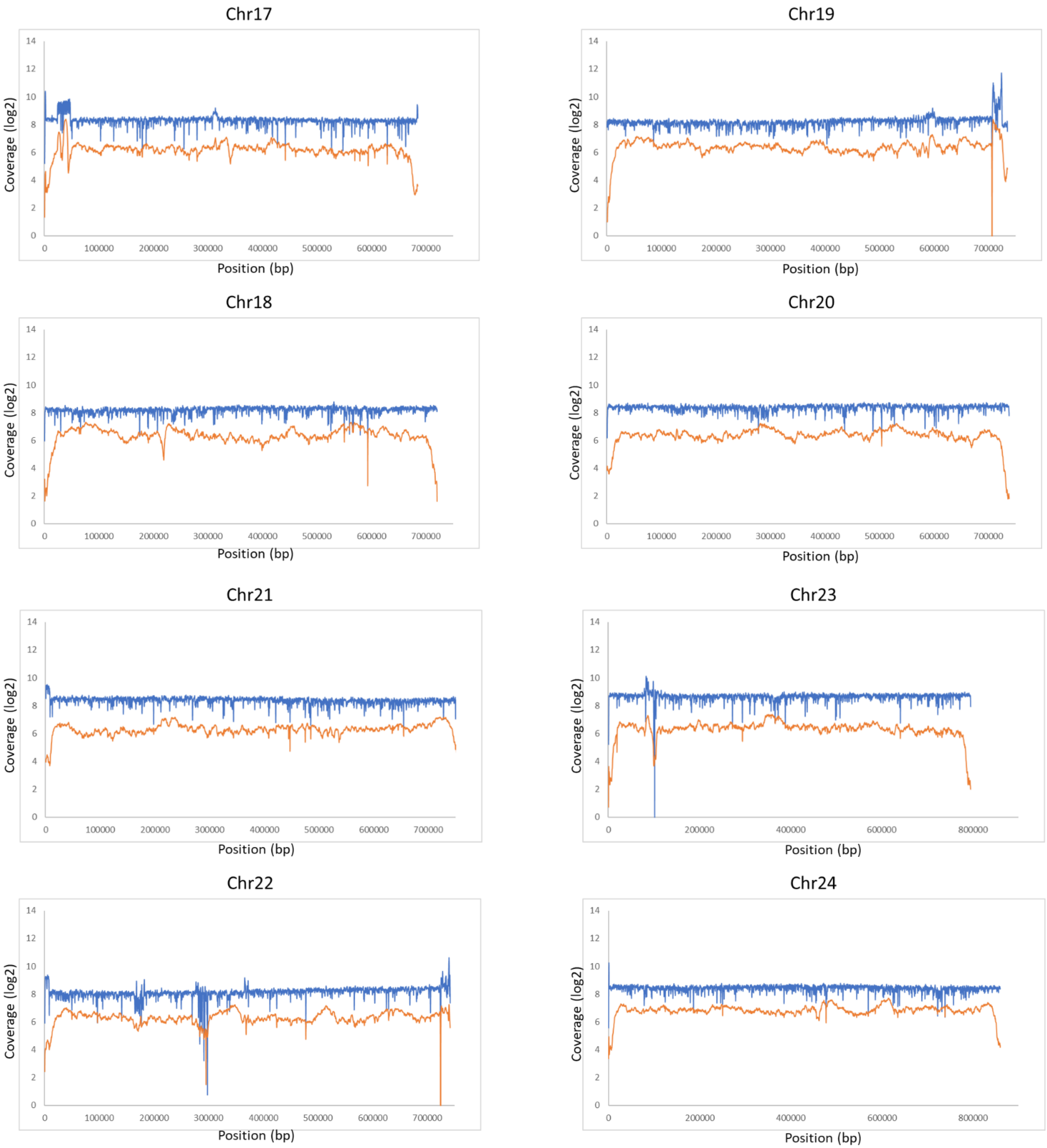
Chr17
Chr19
Chr18
Chr20
Chr21
Chr23
Chr22
Chr24
Coverage (log2)
Position (bp)
0
2
4
6
8
10
12
14
0
100000
200000
300000
400000
500000
600000
700000
800000

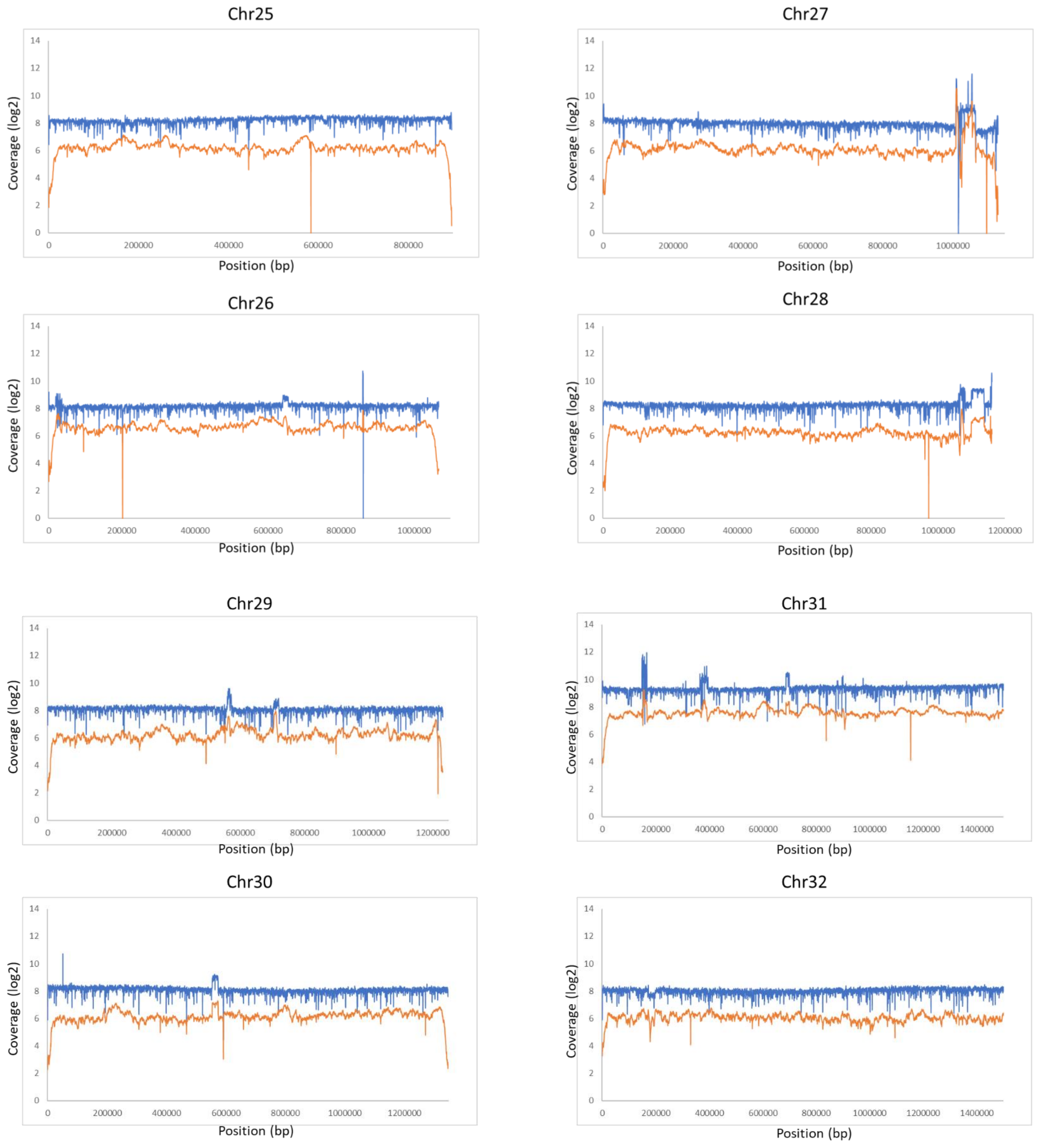

Chr25
Chr27
Chr26
Chr28
Chr29
Chr31
Chr30
Chr32
Coverage (log2)
Position (bp)
0
2
4
6
8
10
12
14
0
200000
400000
600000
800000
1000000
1200000
1400000

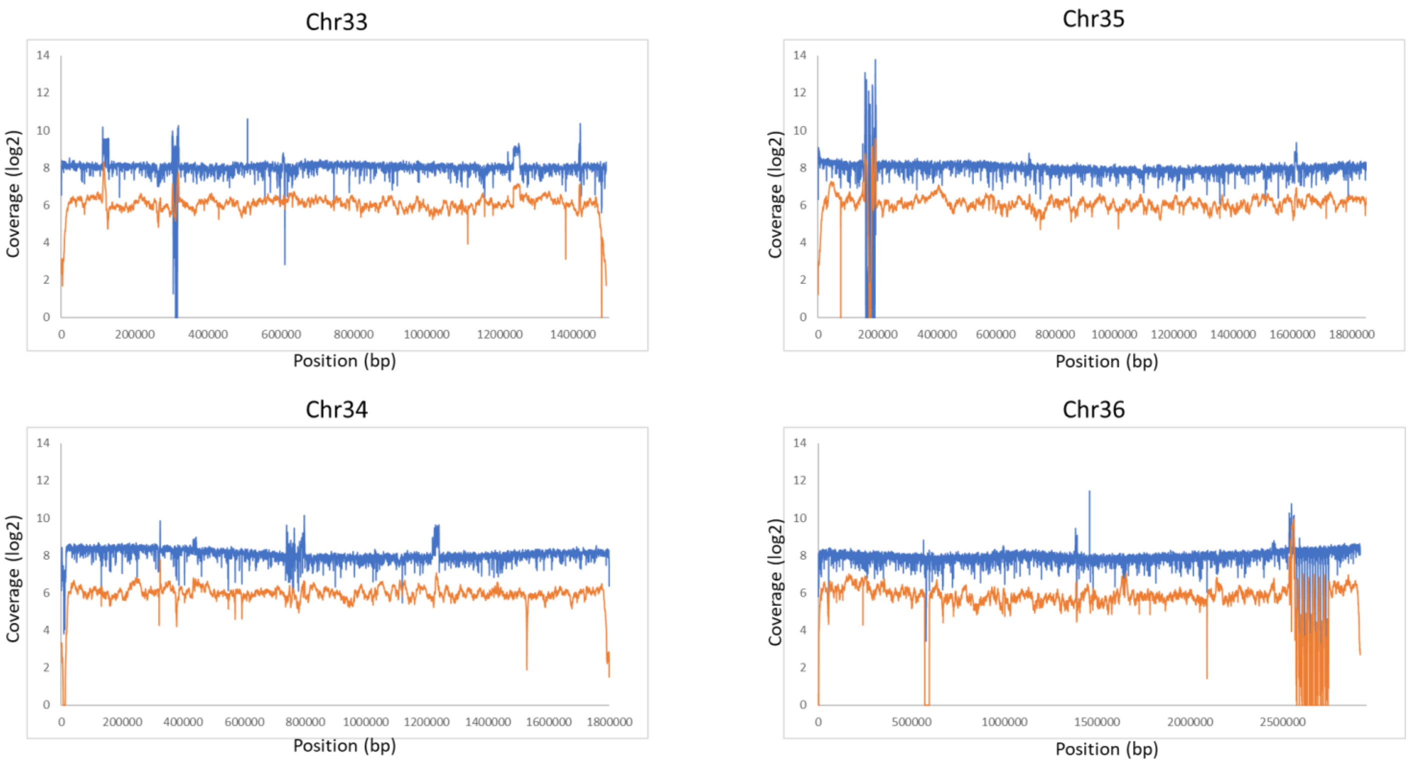

Read coverage of each chromosome in the new assembly. Graphs were generated from the moving average of the raw coverage at each position with a window size of 450bp, equivalent to the average Illumina read insert size. Illumina data plotted in blue, PacBio data plotted in orange.

Supplementary Figure S2. Complete amino acid sequences of A2 genes from the attenuated cutaneous *L. donovani* strain from Sri Lanka

#1

MKIRSVRPLVVLLVCVAAVLALSASAEPHKAA**VD**

VGPLS**VD**
VGPLSVGPQS VGPLS**VD**
VGPQAVGPLS VGPQAVGPLS
VGPQSVGPLS **VD**
VGPQAVGPLS VGPQAVGPLS
VGPQAVGPLS VGPQAVGPLS
VGPQAVGPLS VGPQAVGPLS
VGPQAVGPLS VGPQSVGPLS
VGPQSVGPLS VGPQSVGPLS
VGPQSVGPLS VGPQSVGPLS
VGPQAVGPLS VGPQSVGPLS
VGPQSVGPLS VGPQSVGPLS
VGPQSVGPLS **VD**
VGPQAVGPLS VGPQAVGPLS
VGPQAVGPLS VGPQAVGPLS
VGPQSVGPLS VGPQSVGPLS
VGPQAVGPLS VGPQAVGPLS
VGPQAVGPLS VGPQAVGPLS
VGPQSVGPLS VGPQSVGPLS
VGPQSVGPLS VGPQSVGPLS
VGPQAVGPLS VGPQSVGPLS
VGPQSVGPLS VGPQSVGPLS
VGPQSVGPLS VGPQSVGPLS
VGPQSVGPLS VGPQAVGPLS
VGPQSVGPLS VGPLSVGPQS
VGPLSVGPQS VGPLSVGPQS

VDVSPVS

#2

MKIRSVRPLVVLLVCVAAVLALSASAEPHKAA**VD**

VGPLS**VD**
VGPLSVGPQS VGPLS**VD**
VGPQAVGPLS VGPQAVGPLS
VGPQSVGPLS **VD**
VGPQAVGPLS VGPQAVGPLS
VGPQAVGPLS VGPQAVGPLS
VGPQAVGPLS VGPQAVGPLS
VGPQSVGPLS VGPQSVGPLS
VGPQSVGPLS VGPQAVGPLS
VGPQSVGPLS VGPQSVGPLS
VGPQSVGPLS VGPQSVGPLS
VD
VGPQAVGPLS VGPQAVGPLS
VGPQAVGPLS VGPQSVGPLS
VGPQSVGPLS VGPQAVGPLS
VGPQAVGPLS VGPQSVGPLS
VGPQSVGPLS VGPQSVGPLS
VGPQSVGPLS
VGPQSVGPLS VGPQAVGPLS
VGPQSVGPLS VGPQSVGPLS
VGPQSVGPLS VGPQSVGPLS
VD
VGPQAVGPLS VGPQAVGPLS
VGPQAVGPLS VGPQSVGPLS
VGPQSVGPLS VGPQAVGPLS
VGPQAVGPLS VGPQAVGPLS
VGPQAVGPLS VGPQSVGPLS
VGPQSVGPLS VGPQSVGPLS
VGPQSVGPLS VGPQAVGPLS
VGPQSVGPLS VGPQSVGPLS
VGPQSVGPLS VGPQAVGPLS
VGPQSVGPLS VGPQSVGPLS
VGPQAVGPLS VGPQSVGPLS
VGPLSVGPQS VGPLSVGPQS

VDVSPVS

#3

MKIRSVRPLVVLLVCVAAVLALSASAEPHKAA**VD**

VGPLS**VD**
VGPLSVGPQS VGPLS**VD**
VGPLSVGPQA VGPLS**VD**
VGPQAVGPLS VGPQSVGPLS
VGPQSVGPLS VGPLSVGPLS
VGPQSVGPLS VGPQAVGPLS
VD
VGPQAVGPLS VGPQSVGPLS
VGPQAVGPLS VGPQSVGPLS
VD
VGPQAVGPLS VGPQSVGPLS
VD
VGPQSVGPLS VGPQSVGPLS
VGPQSVGPLS **VD**
VGPQAVGPLS VGPQSVGPLS
VGPLSVGPQS VGPLSVGPQS
VGPLS**VD**
VGPQAVGPLS VGPQSVGPLS
VGPQAVGPLS VGPQAVGPLS
VGPQAVGPLS VGPLSVGPLS
VGPLSVGPQS VGPLSVGPQS
VGPLSVGPQS VGPLSVGPQS
VGPLSVGPQS VGPLSVGPQS
VGPLSVGPQS VGPLSVGPQS
VGPLSVGPQS VGPLSVGPQS
VGPLSVGPQS VGPLSVGPQS
VGPLSVGPQS VGPLSVGPQS
VGPLSVGPQA VGPLSVGPQS
VGPLSVGPQS VGPLSVGPQS
VGPLSVGPQS VGPLSVGPQS
VGPLSVGPQA VGPLSVGPQS
VGPLSVGPQA VGPLS**VD**
VGPQAVGPLS VGPQSVGPLS
VGPQSVGPLS VGPQAVGPLS
VGPQSVGPLS VGPLSVGPQS
VGPLSVGPQS

VDVSPVS

#4

MKIRSVRPLVVLLVCVAAVLALSASAEPHKAA**VD**

VGPLS**VD**
VGPLSVGPQS VGPLS**VD**
VGPLSVGPQA VGPLS**VD**
VGPQAVGPLS **VD**
VGPQAVGPLS VGPQSVGPLS
VGPQSVGPLS VGPLSVGPLS
VGPQSVGPLS VGPQSVGPLS
VD
VGPQAVGPLS VGPQSVGPIS
VGPQAVGPLS VGPQAVGPLS
VGPQSVGPLS VGPQSVGPLS
VGPQSVGPLS VGPQSVGPLS
VGPLSVGPLS VGPLSVGPQS
VGPLSVGPQS VGPLSVGPQS
VGPLSVGPQS VGPLSVGPQS
VGPLSVGPQS VGPLSVGPQS
VGPLSVGPQS VGPLSVGPQS
VGPLSVGPQS VGPLSVGPQS
VGPLSVGPQS VGPLSVGPQS
VGPLSVGPQS VGPLSVGPQS
VGPLSVGPQA VGPLSVGPQS
VGPLSVGPQS VGPLSVGPQS
VGPLSVGPQS VGPLSVGPQS
VGPLSVGPQA VGPLSVGPQS
VGPLSVGPQS VGPLSVGPQS
VGPLSVGPQS VGPLSVGPQA
VGPLSVGPQS VGPLSVGPQA
VGPLS**VD**
VGPQAVGPLS VGPQSVGPLS
VGPQSVGPLS VGPQAVGPLS
VGPQSVGPLS VGPLSVGPQS
VGPLSVGPQS

VDVSPVS

The DNA sequences obtained from the assembled A2 cluster were translated into their corresponding amino acids. All four A2 proteins share a common leader and terminal sequence. Deviations from the VGPLS VGPQS/A by the insertion of valine-aspartate are highlighted in bold.

Supplementary Figure S3. Synteny map of the *L. major* and *L. donovani* chromosomes.

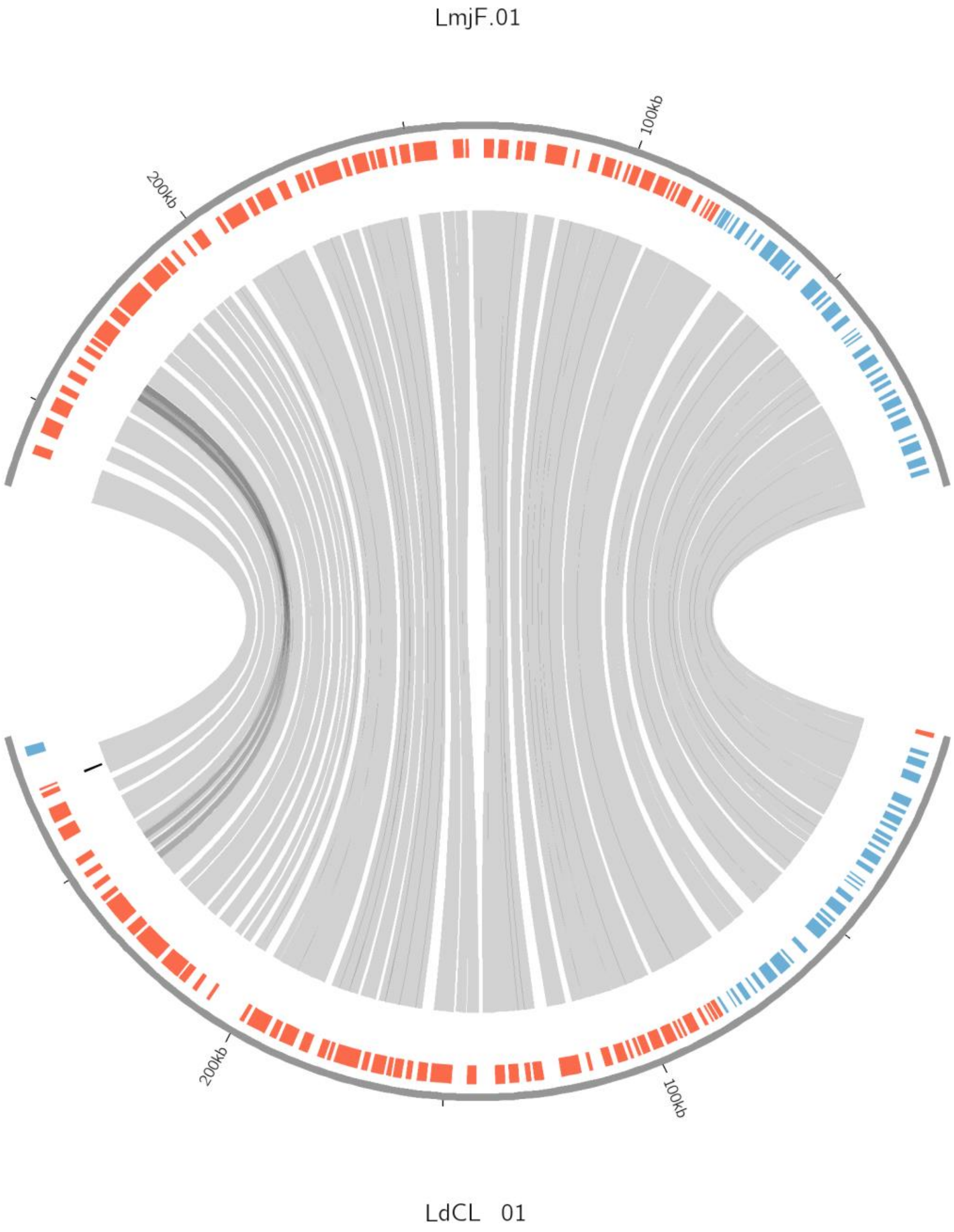

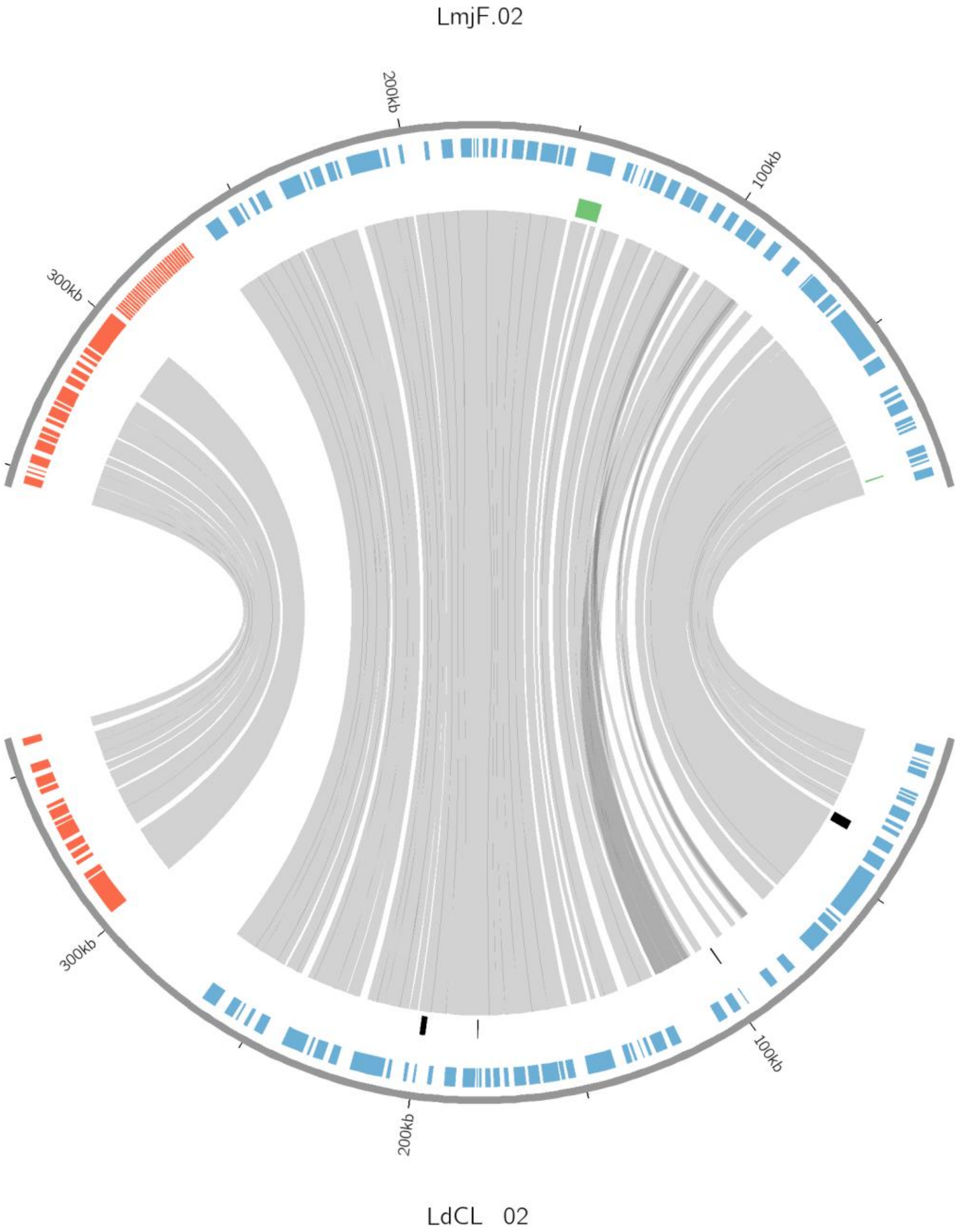
LmjF.02
200kb
100kb
300kb
300kb
100kb
200kb
LdCL 02

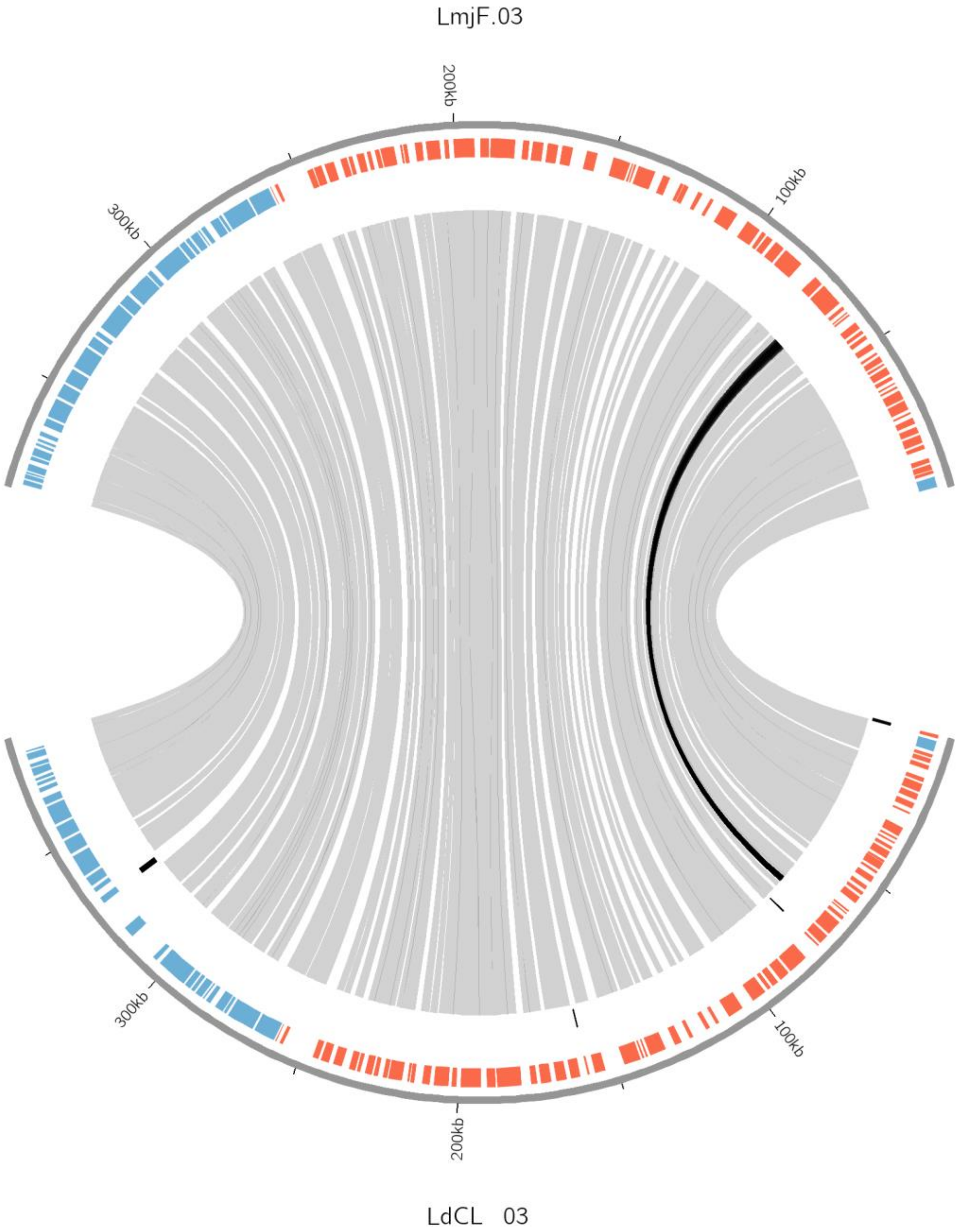
LmjF.03
200kb
100kb
300kb
300kb
100kb
200kb
LdCL 03

LmjF.04

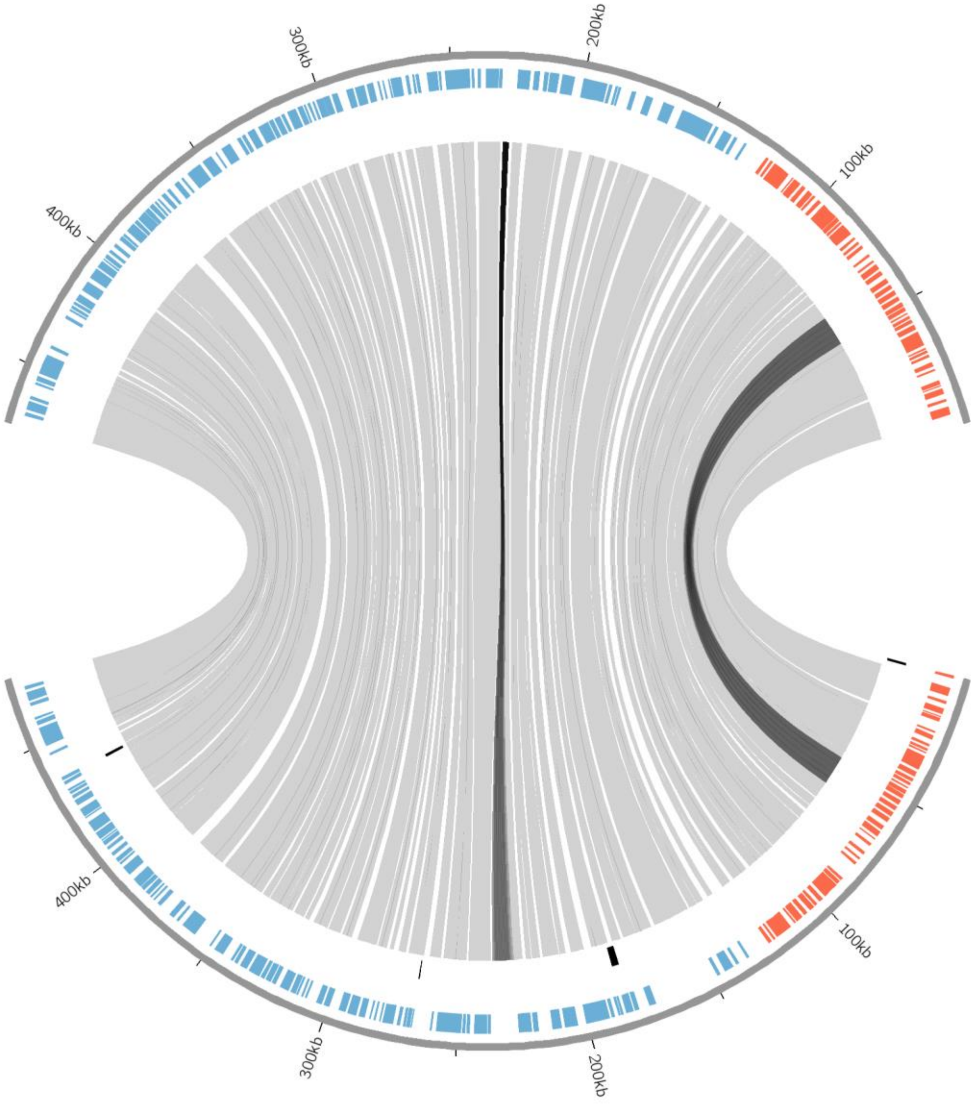

LdCL 04

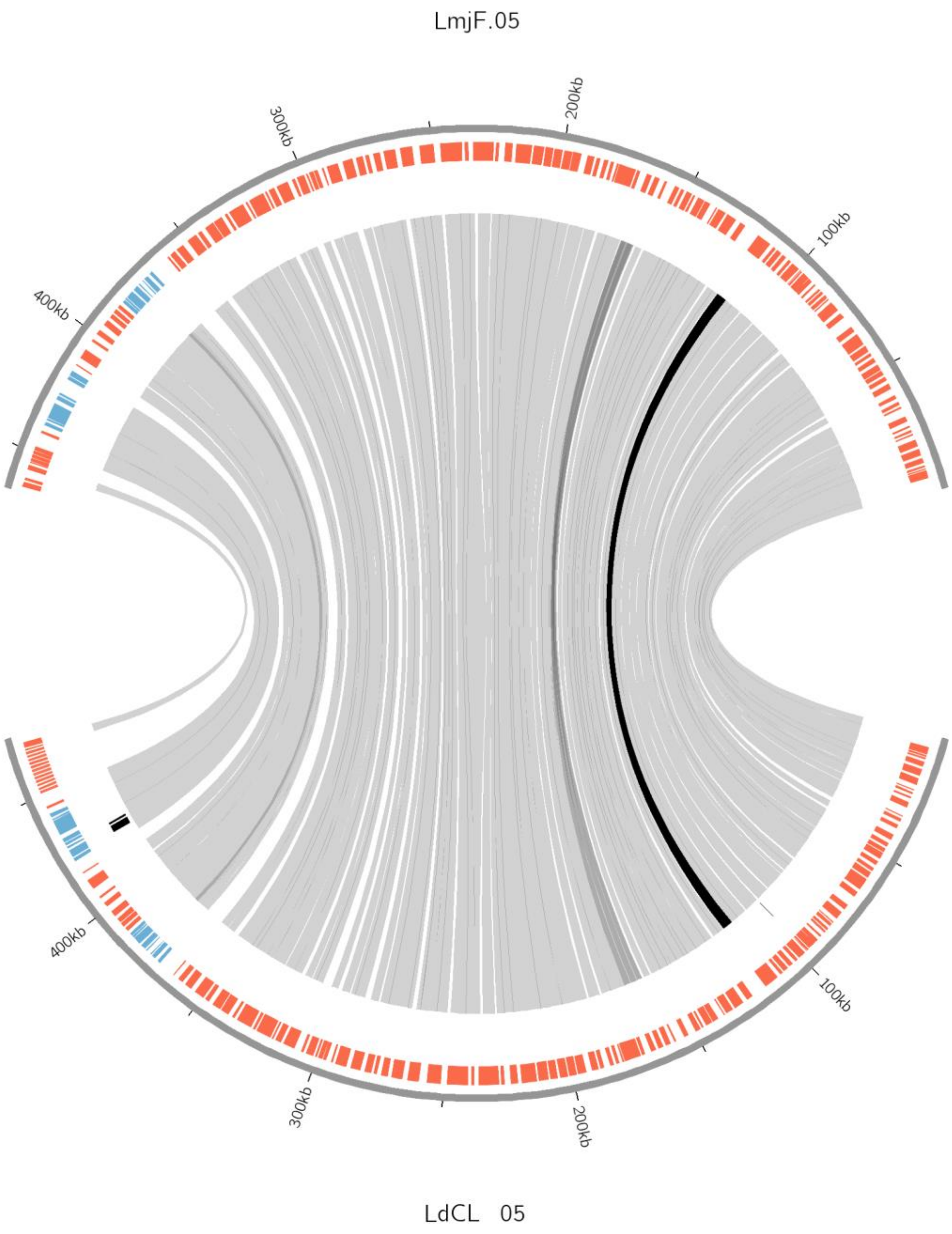
LmjF.05
100kb
200kb
300kb
400kb
100kb
200kb
300kb
400kb
LdCL 05

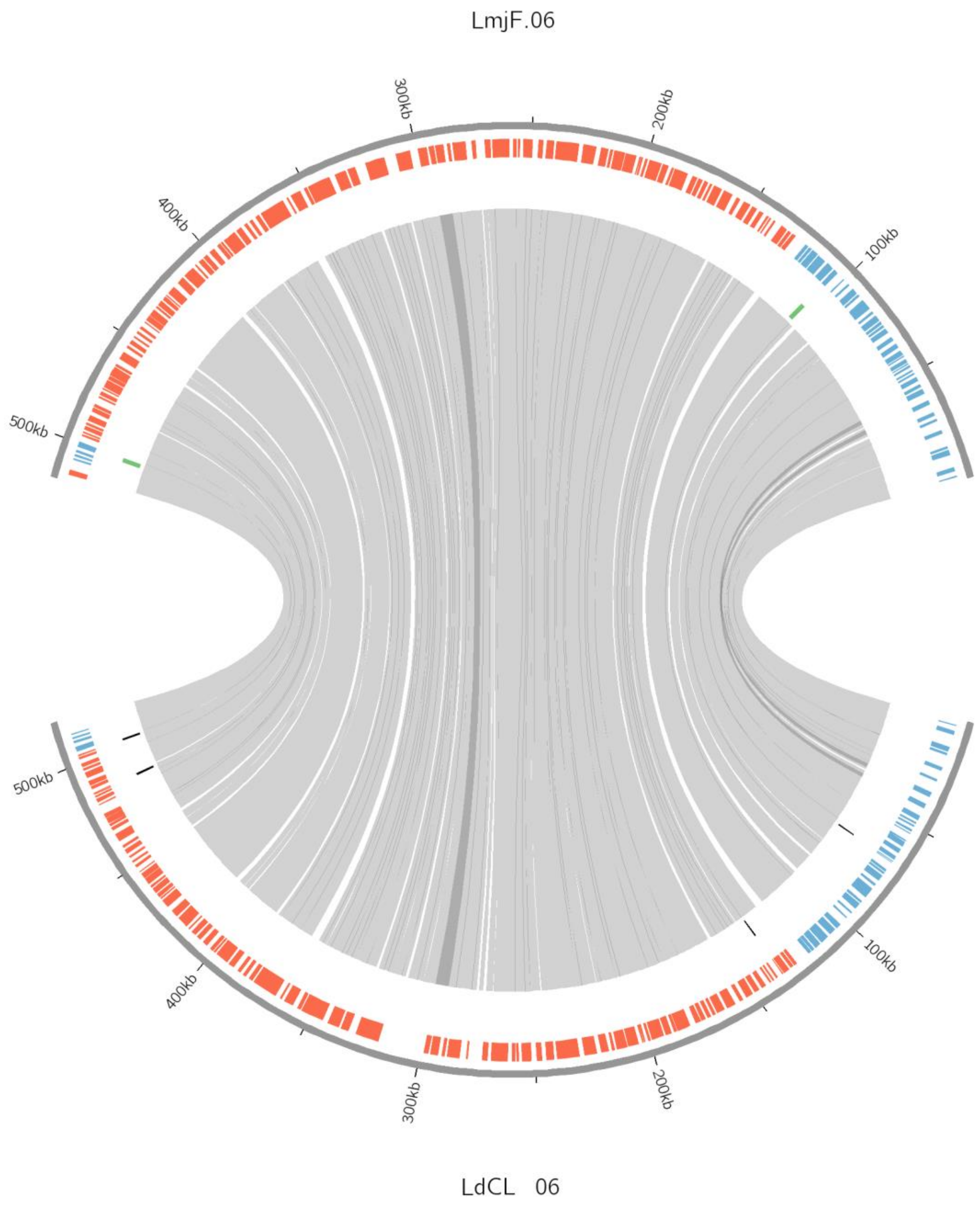
LmjF.06
300kb
200kb
400kb
100kb
500kb
500kb
100kb
400kb
300kb
200kb
LdCL 06

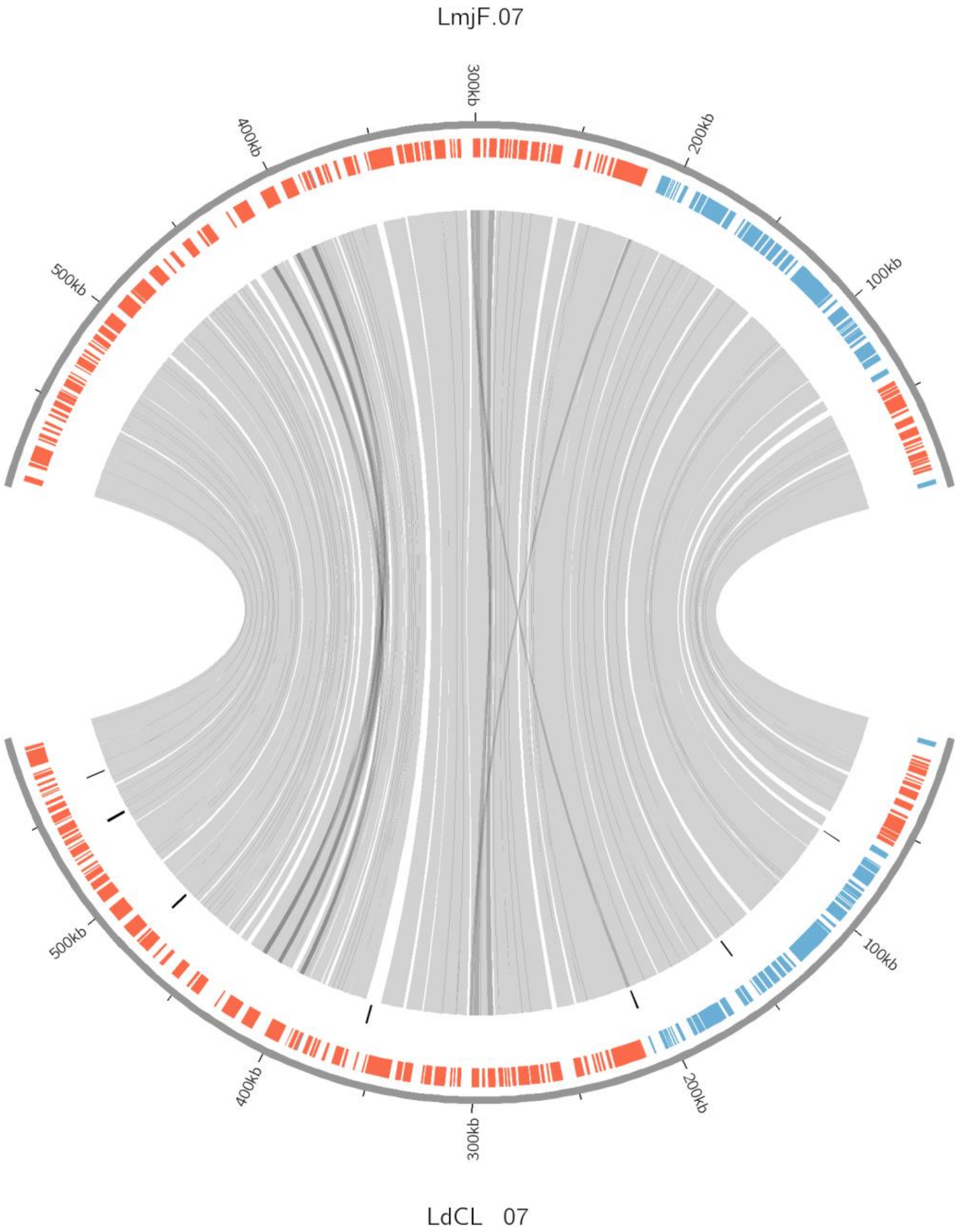
LmjF.07
300kb
200kb
100kb
400kb
500kb
500kb
100kb
400kb
200kb
300kb
LdCL 07

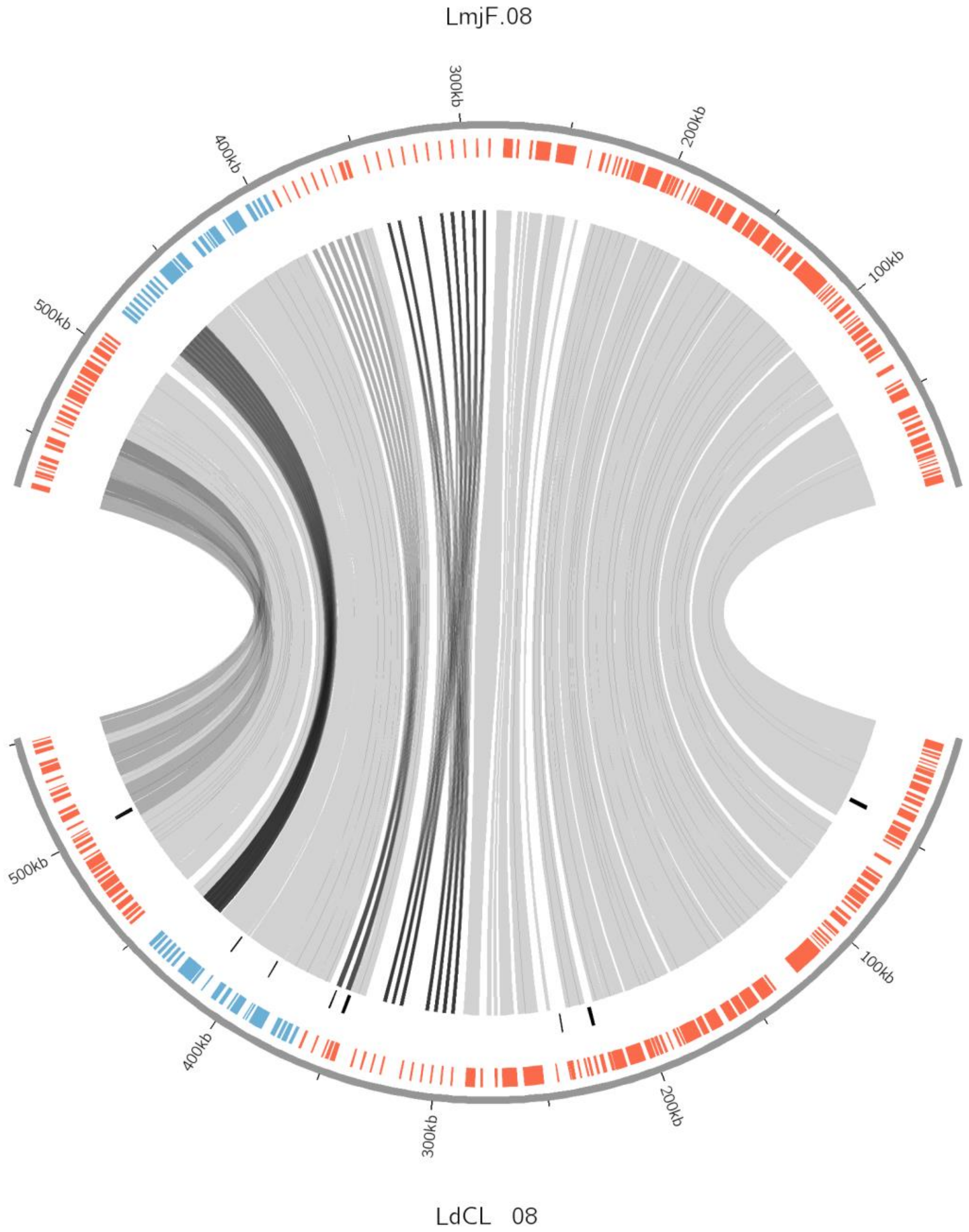
LmjF.08
300kb
200kb
100kb
400kb
500kb
500kb
100kb
400kb
300kb
200kb
LdCL 08

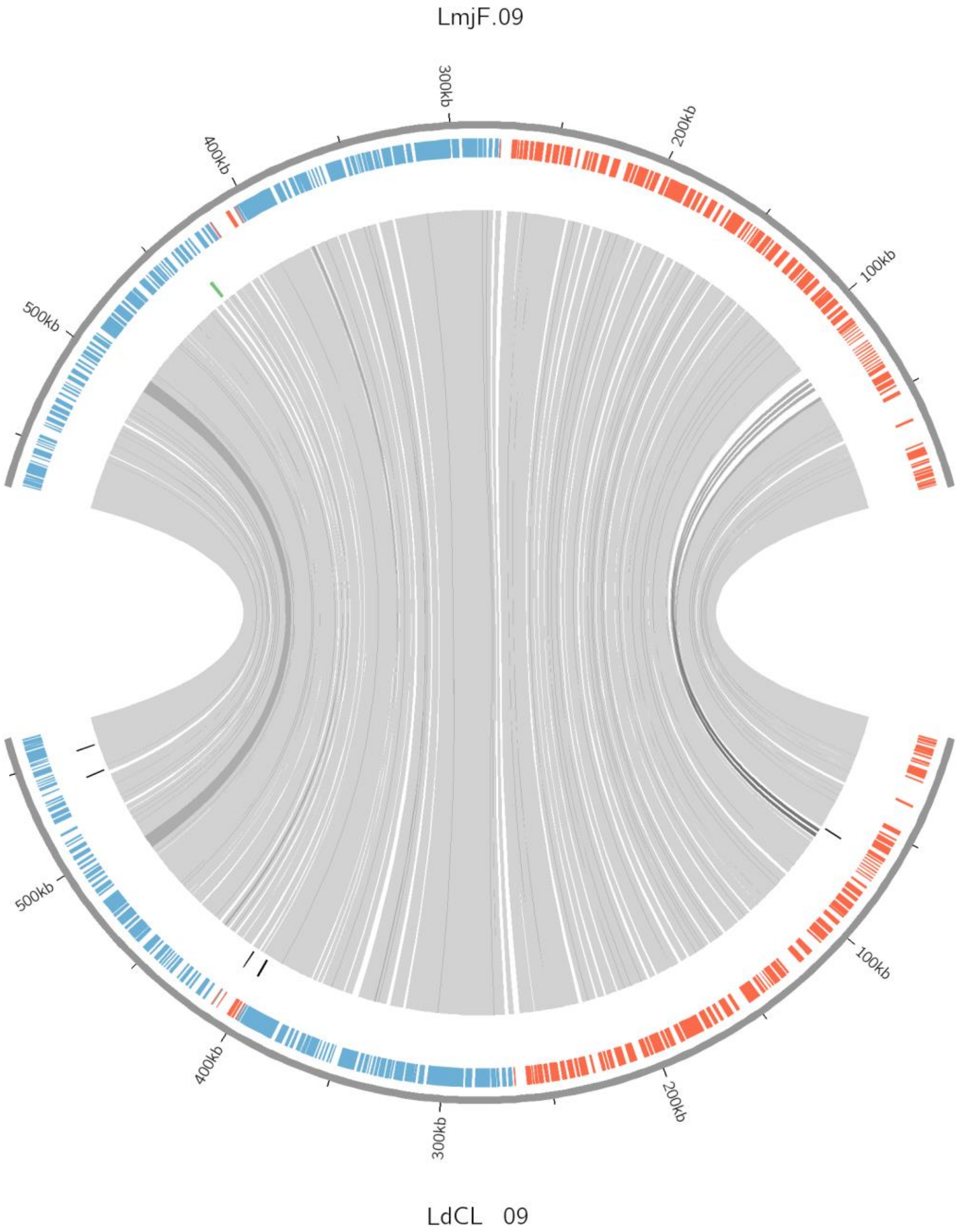

LmjF.09
300kb
200kb
400kb
100kb
500kb
500kb
100kb
400kb
200kb
300kb
LdCL 09

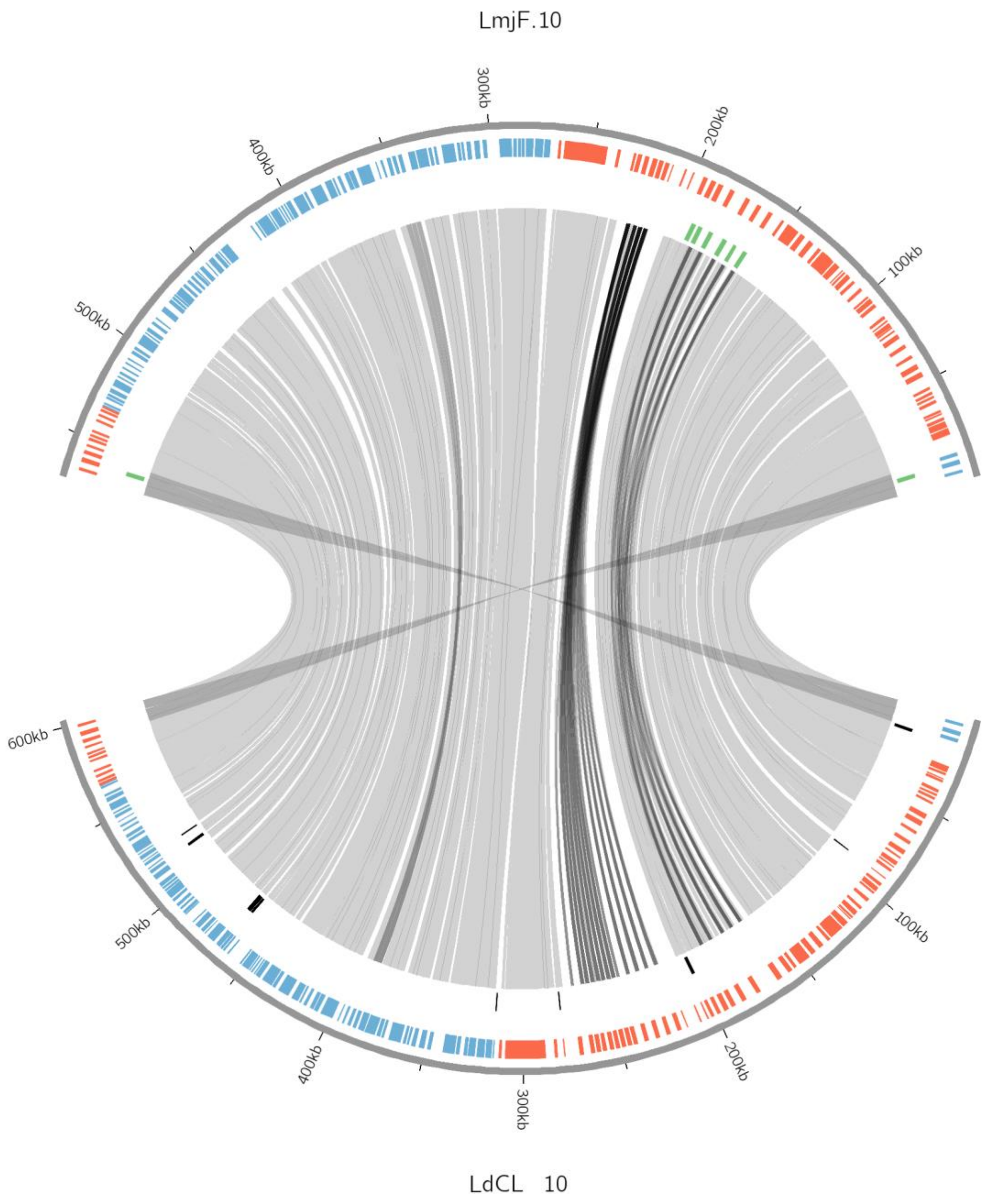
LmjF.10
300kb
200kb
400kb
100kb
500kb
600kb
500kb
100kb
400kb
200kb
300kb
LdCL 10

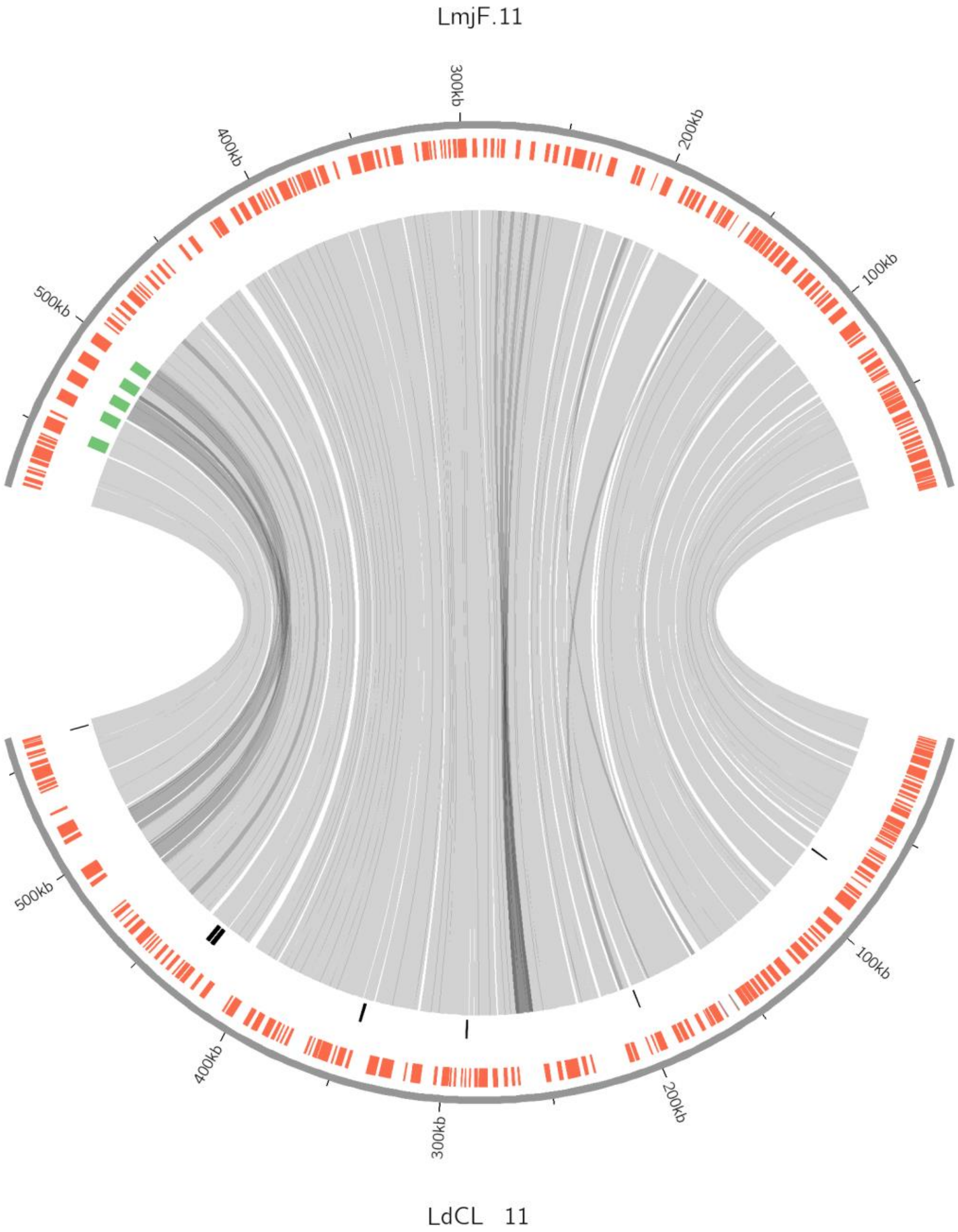

LmjF.11
300kb
200kb
400kb
100kb
500kb
500kb
100kb
400kb
200kb
300kb
LdCL 11

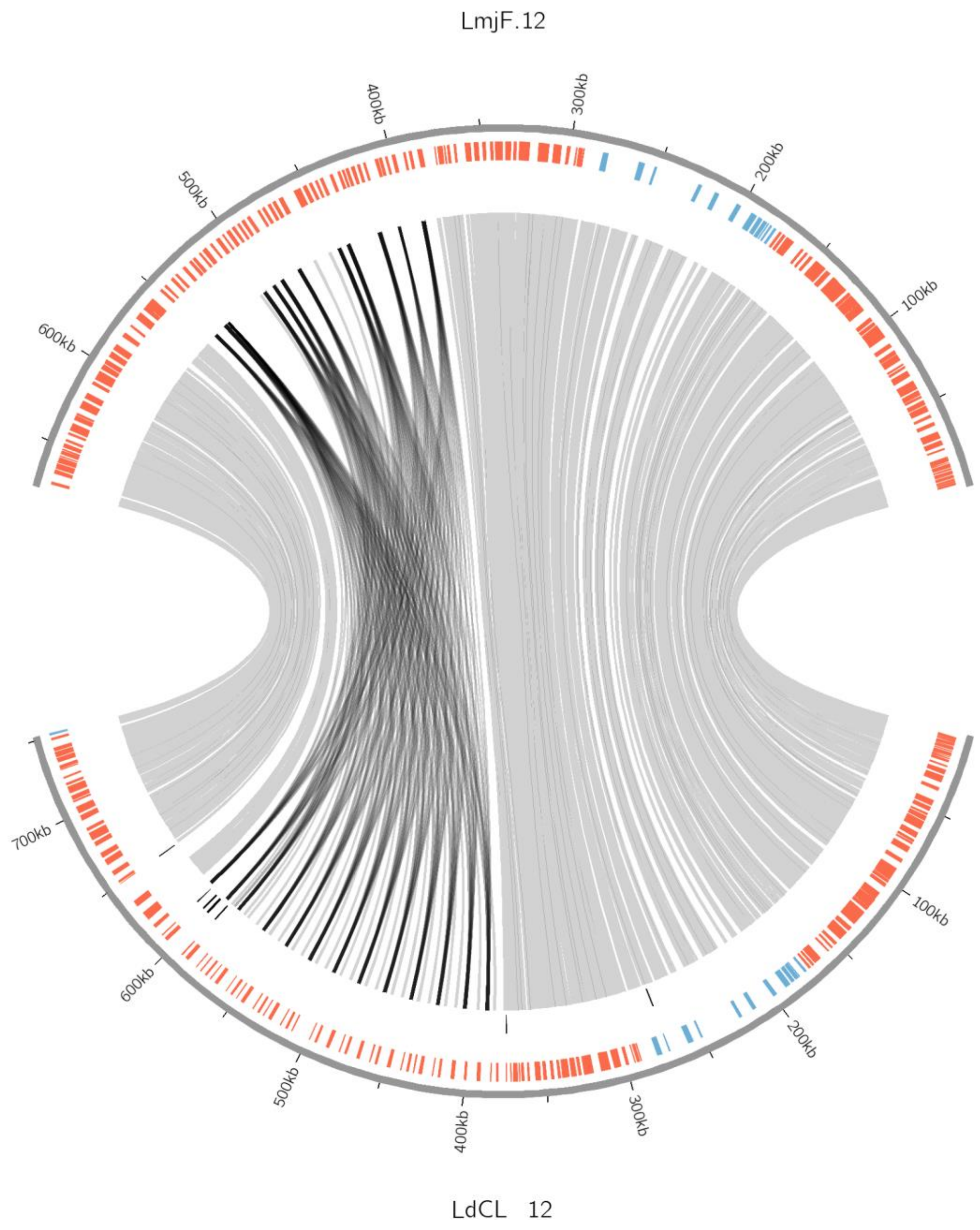
LmjF.12
100kb
200kb
300kb
400kb
500kb
600kb
700kb
100kb
200kb
300kb
400kb
500kb
600kb
LdCL 12

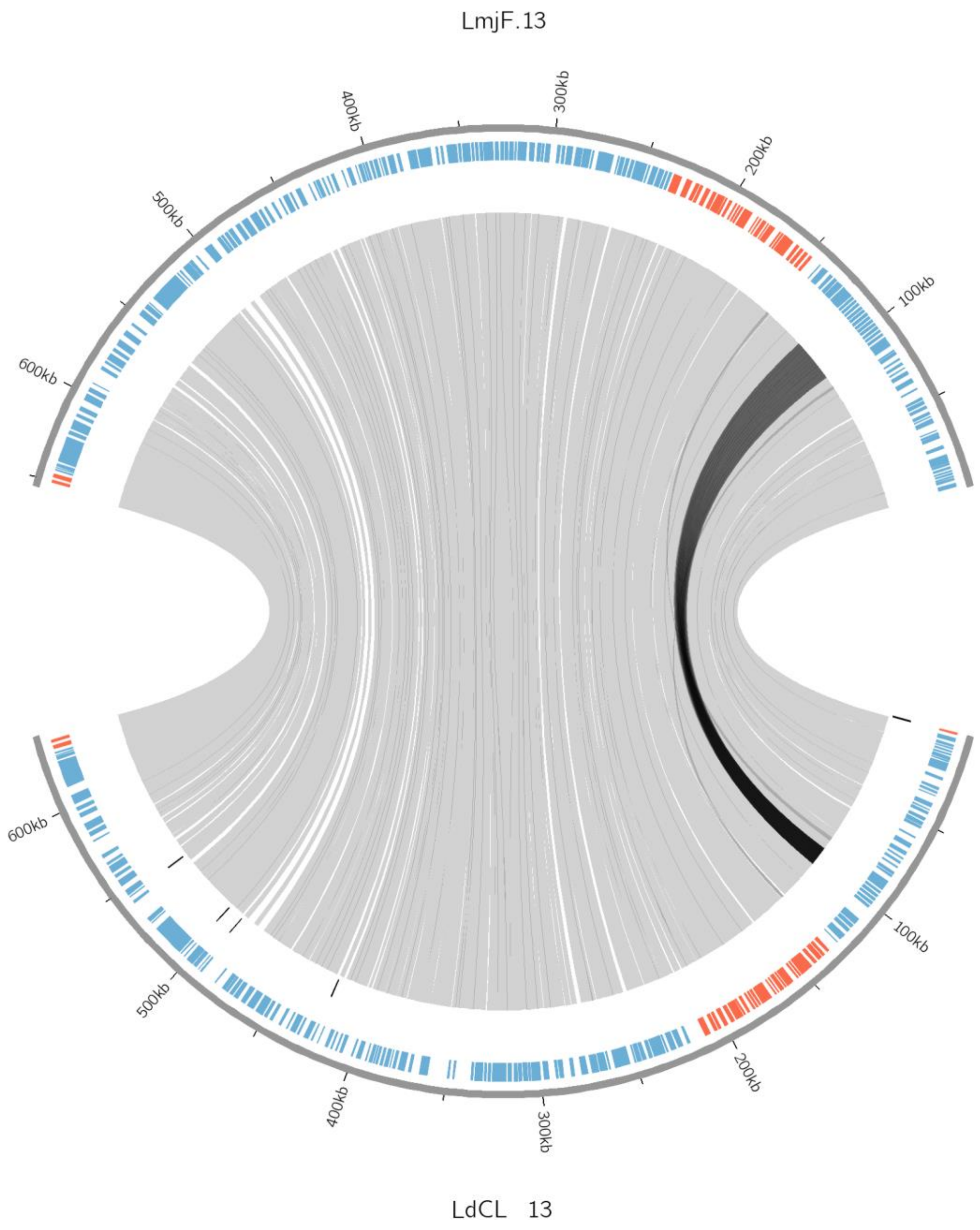
LmjF.13
100kb
200kb
300kb
400kb
500kb
600kb
LdCL 13
100kb
200kb
300kb
400kb
500kb
600kb

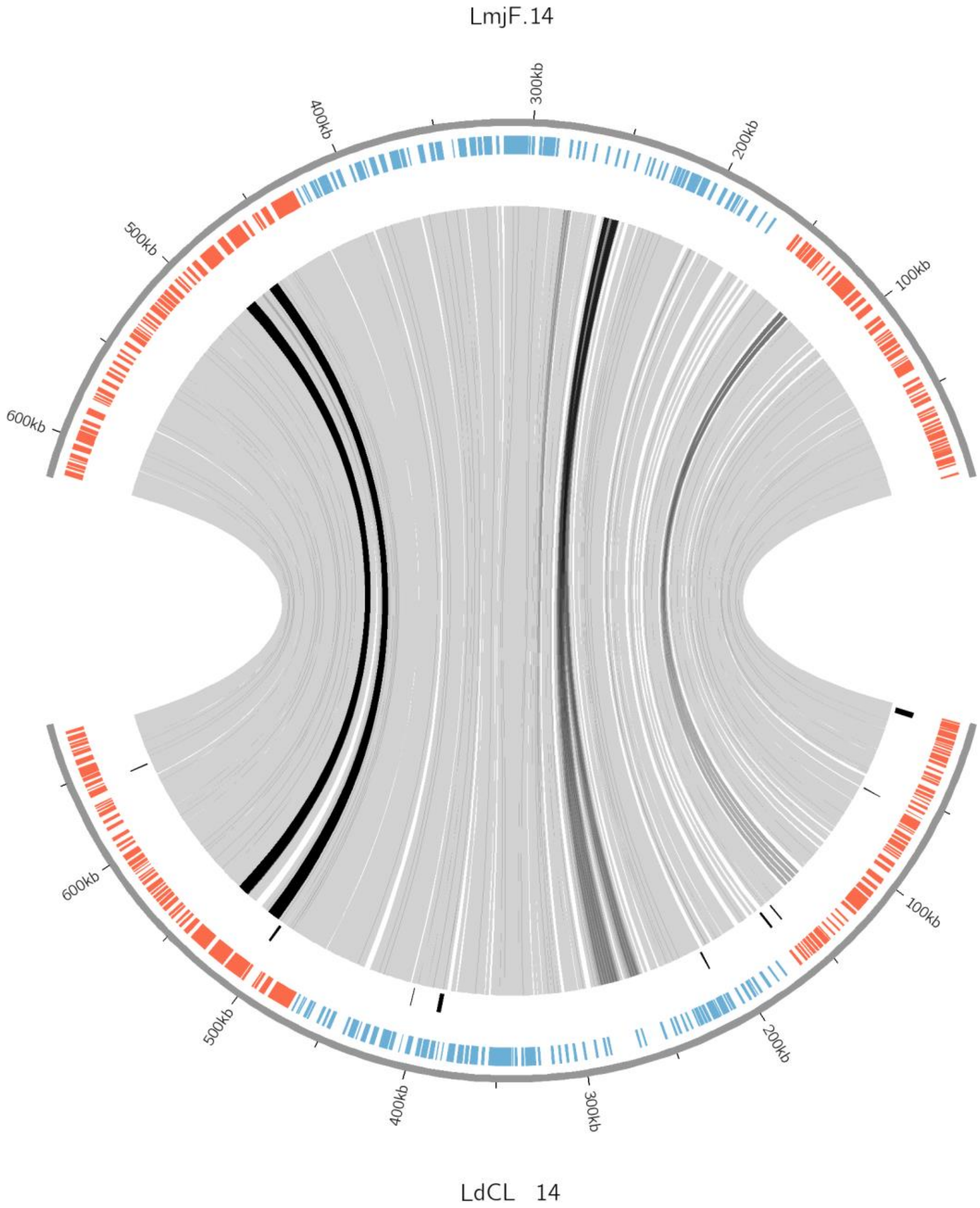
LmjF.14
100kb
200kb
300kb
400kb
500kb
600kb
100kb
200kb
300kb
400kb
500kb
600kb
LdCL 14

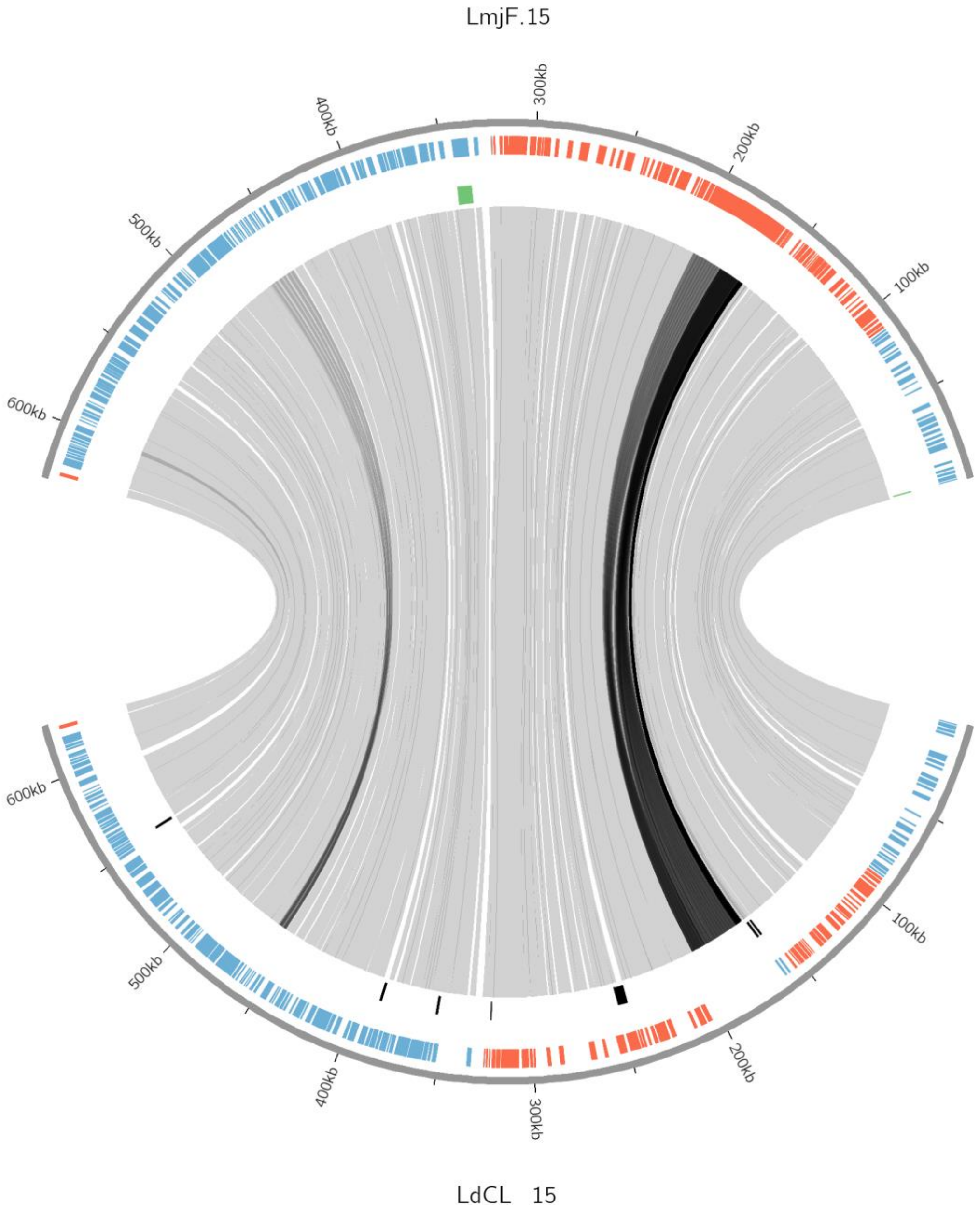
LmjF.15
300kb
200kb
100kb
400kb
500kb
600kb
600kb
500kb
400kb
300kb
200kb
100kb
LdCL 15

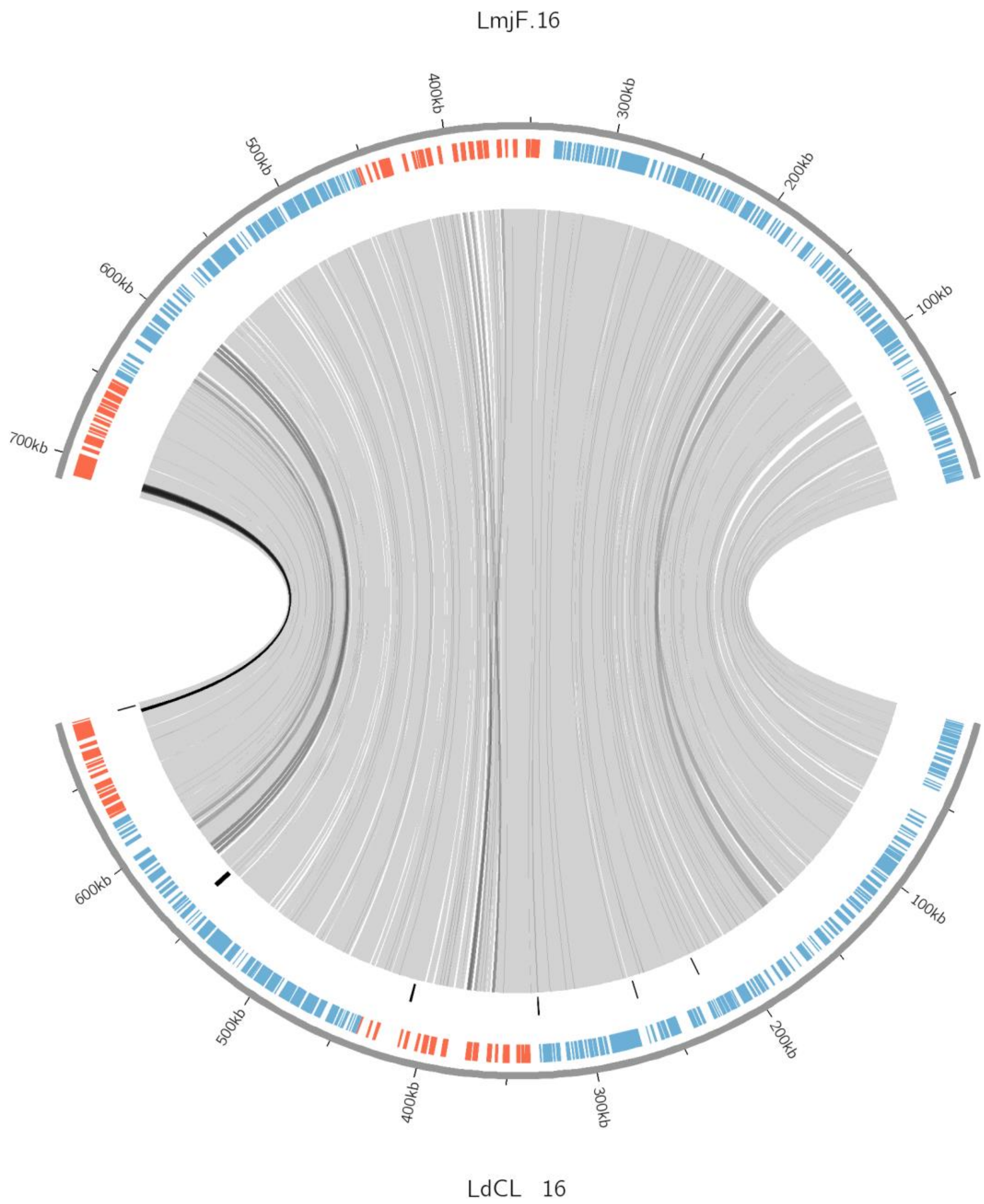
LmjF.16
400kb
300kb
500kb
200kb
600kb
100kb
700kb
600kb
100kb
500kb
200kb
400kb
300kb
LdCL 16

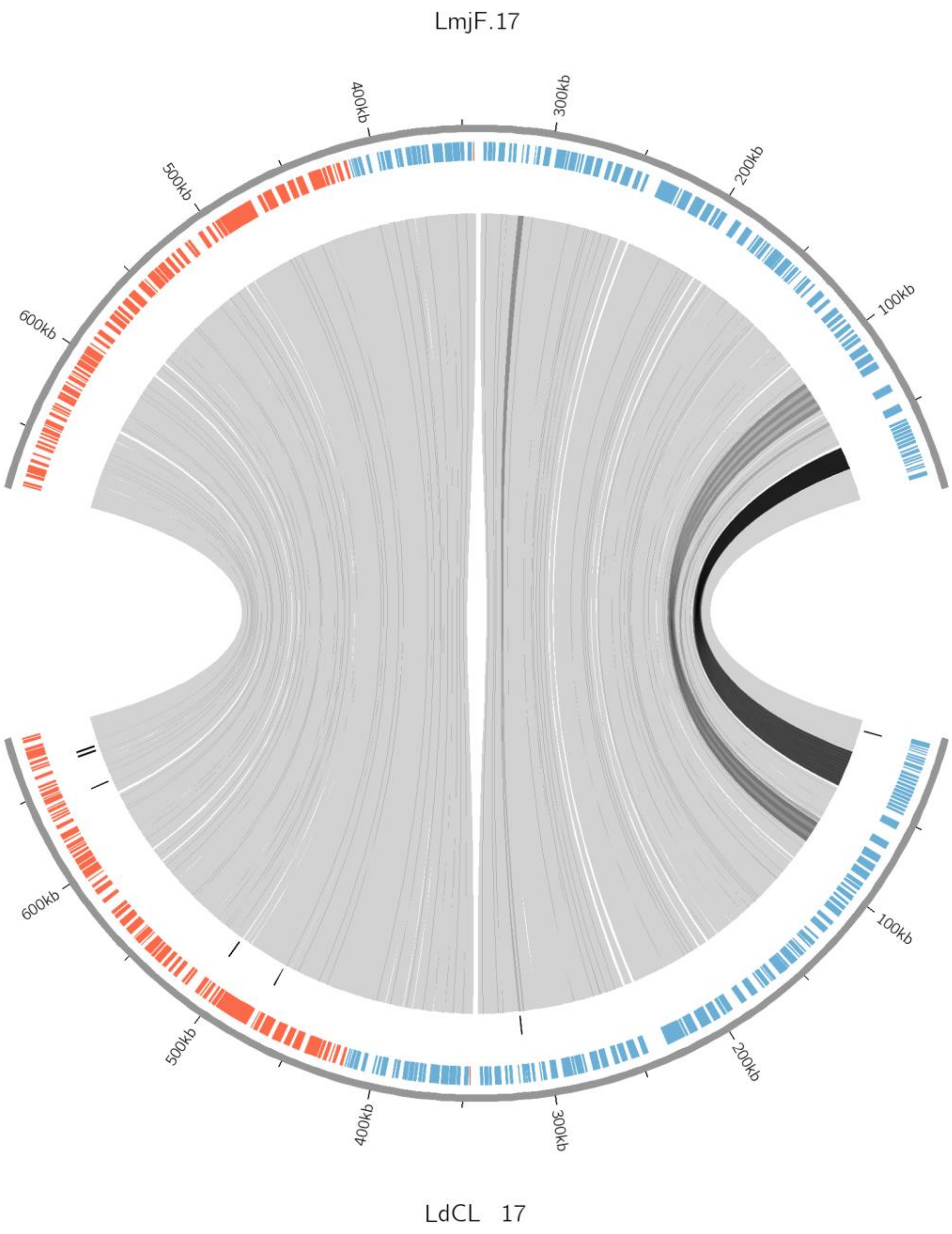
LmjF.17
400kb
300kb
200kb
100kb
500kb
600kb
600kb
500kb
400kb
300kb
200kb
100kb
LdCL 17

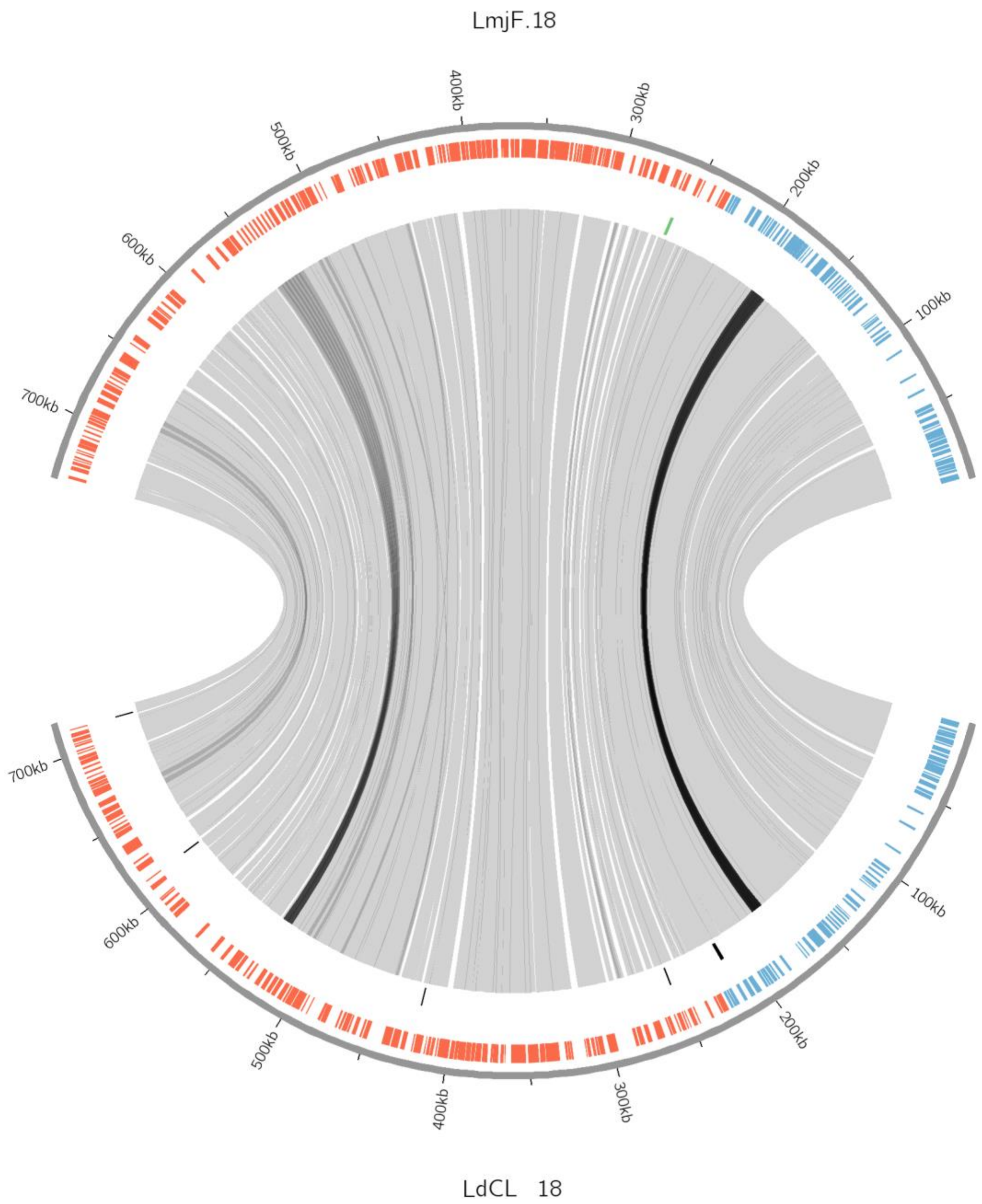

LmjF.18
100kb
200kb
300kb
400kb
500kb
600kb
700kb
100kb
200kb
300kb
400kb
500kb
600kb
700kb
LdCL 18

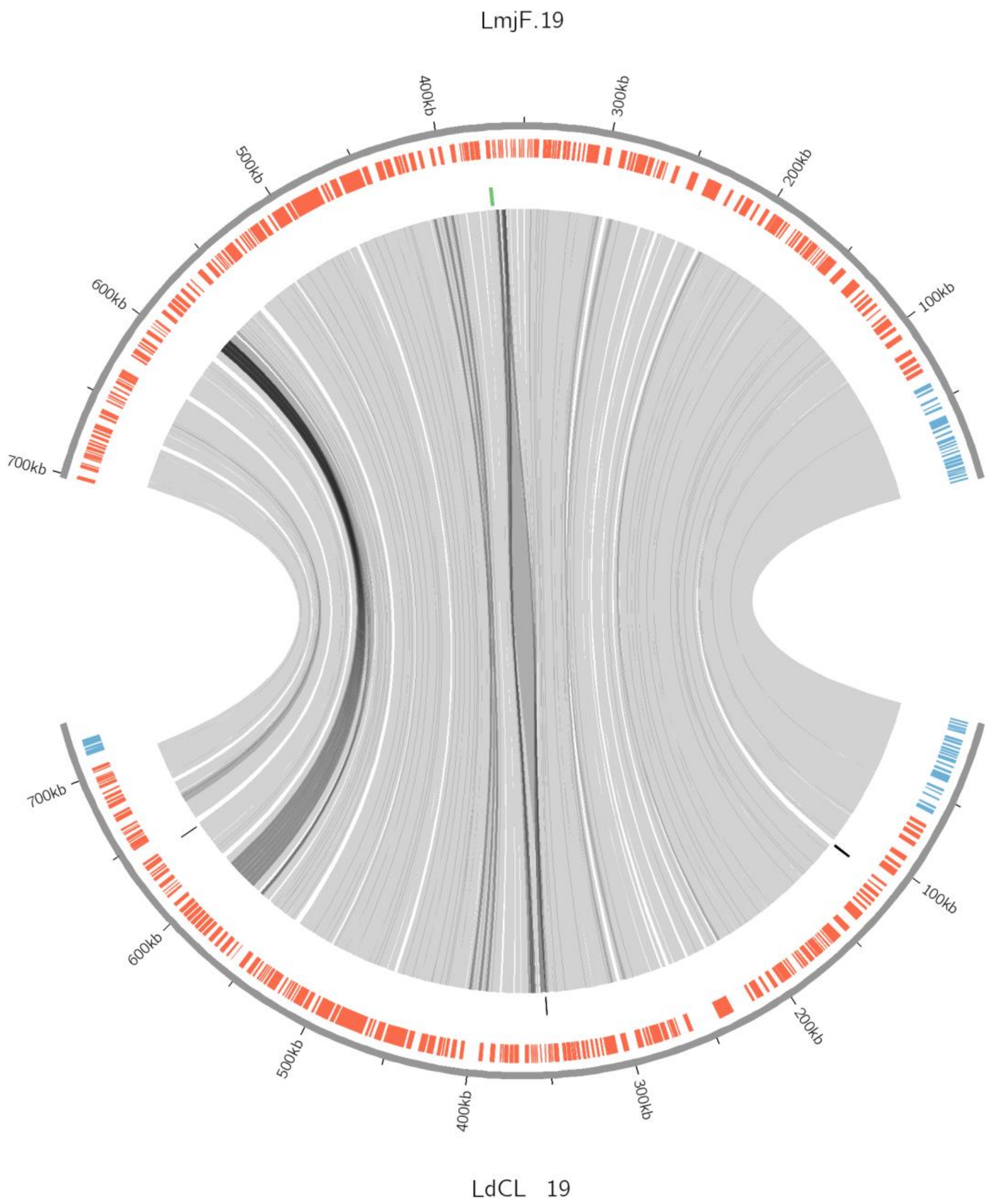
LmjF.19
100kb
200kb
300kb
400kb
500kb
600kb
700kb
LdCL 19

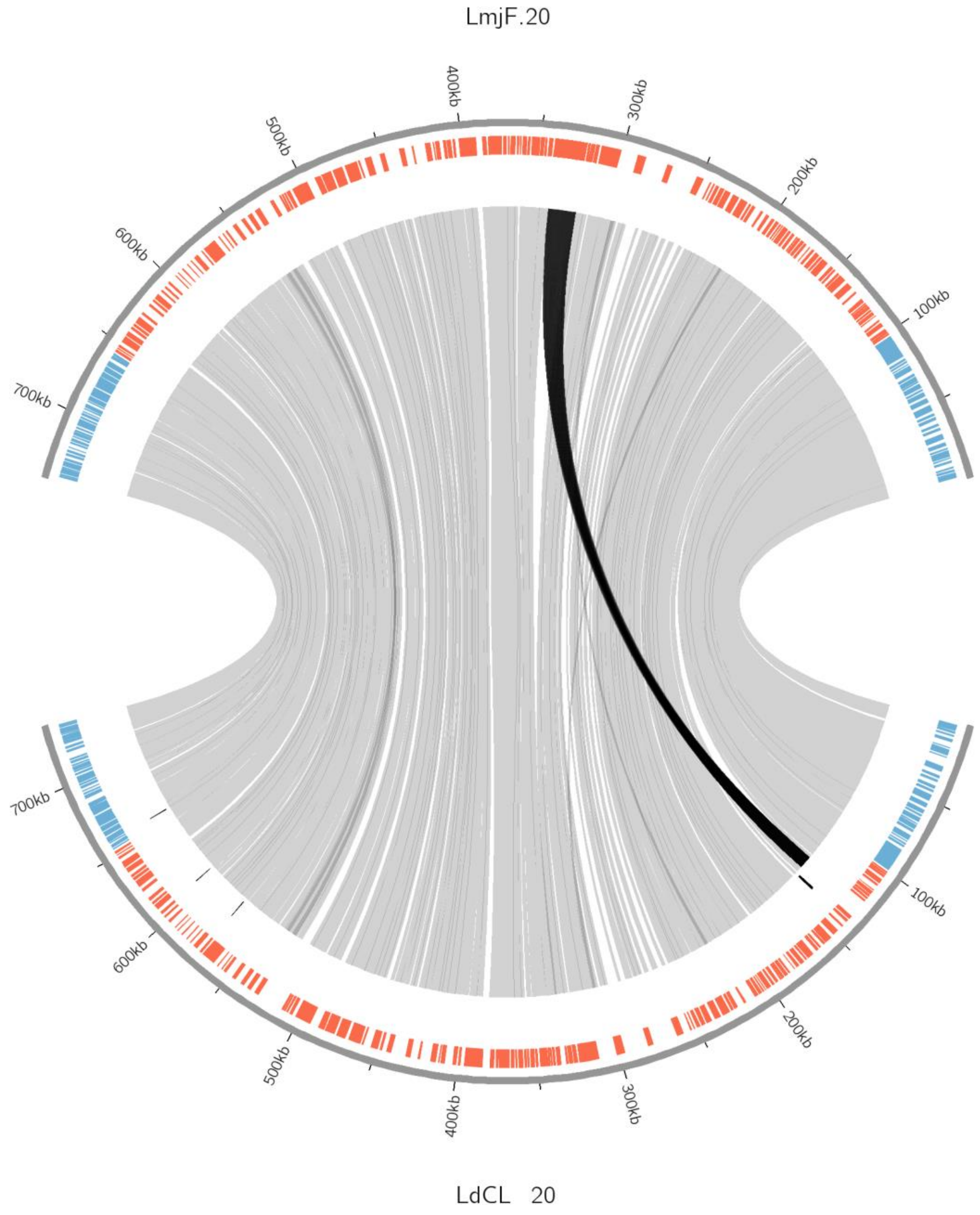

LmjF.20
400kb
300kb
500kb
200kb
600kb
100kb
700kb
700kb
100kb
600kb
200kb
500kb
300kb
400kb
LdCL 20

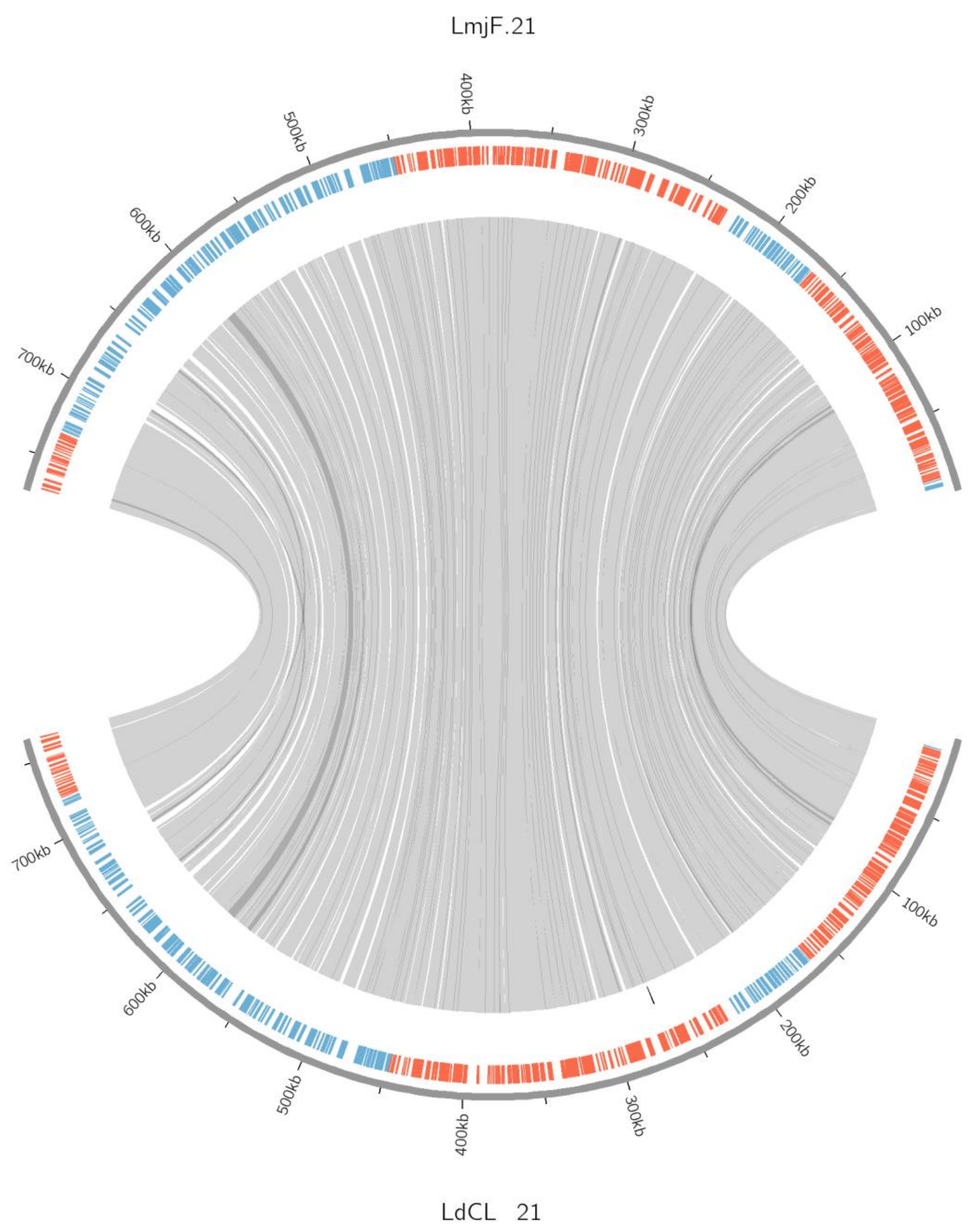

LmjF.21
400kb
300kb
200kb
100kb
500kb
600kb
700kb
LdCL 21

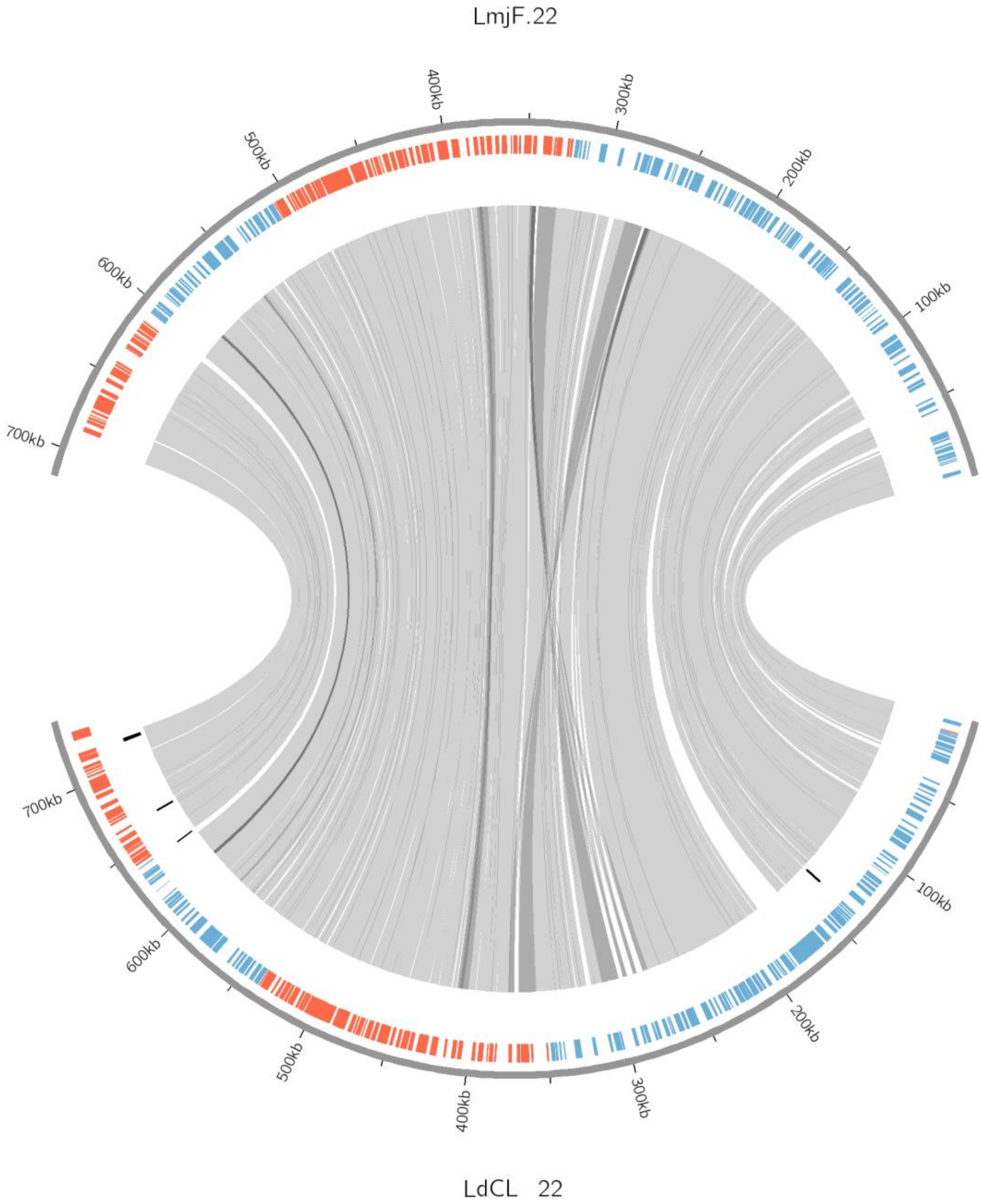

LmjF.22
400kb
300kb
500kb
200kb
600kb
100kb
700kb
700kb
100kb
600kb
200kb
500kb
400kb
300kb
LdCL 22

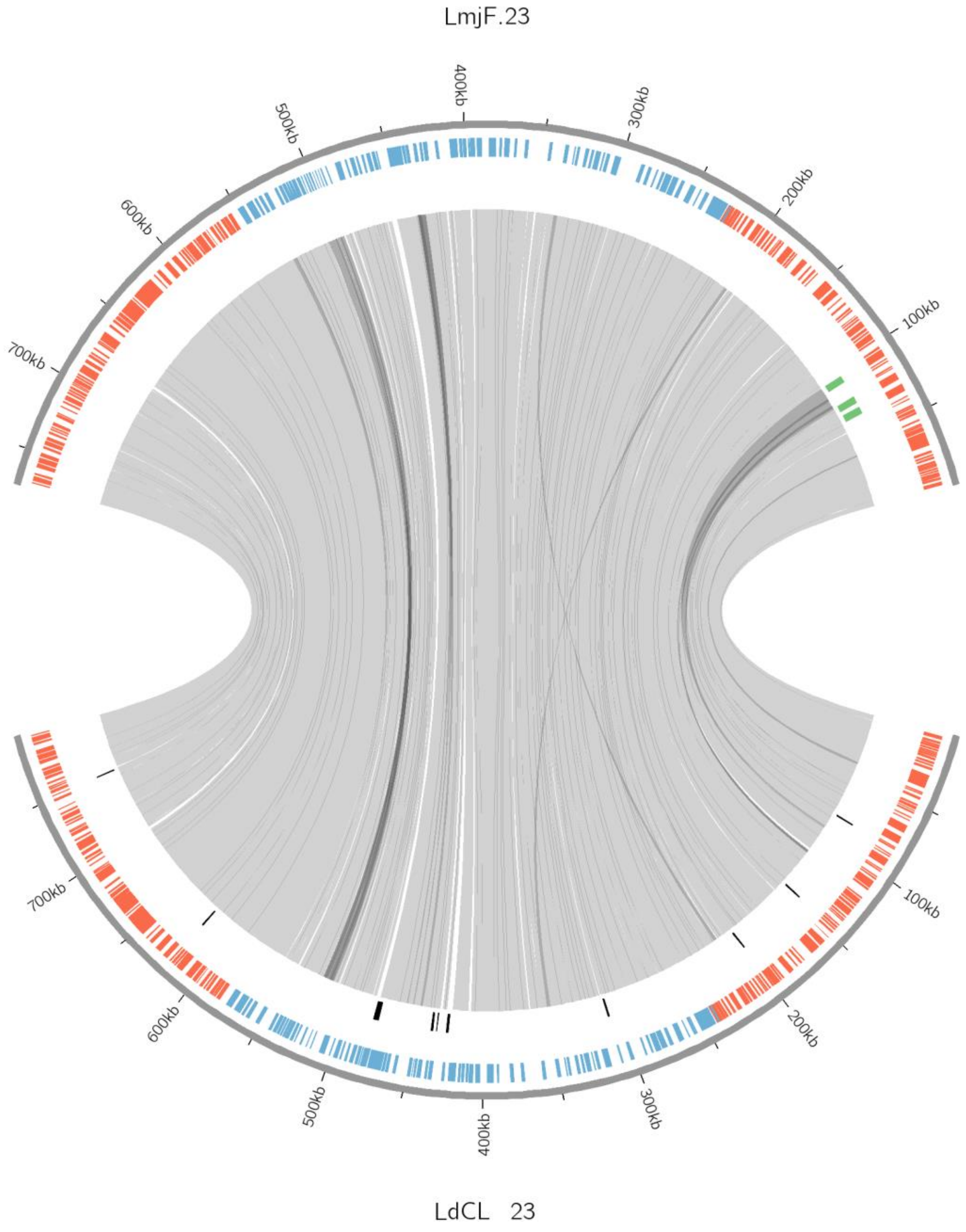
LmjF.23
100kb
200kb
300kb
400kb
500kb
600kb
700kb
100kb
200kb
300kb
400kb
500kb
600kb
700kb
LdCL 23

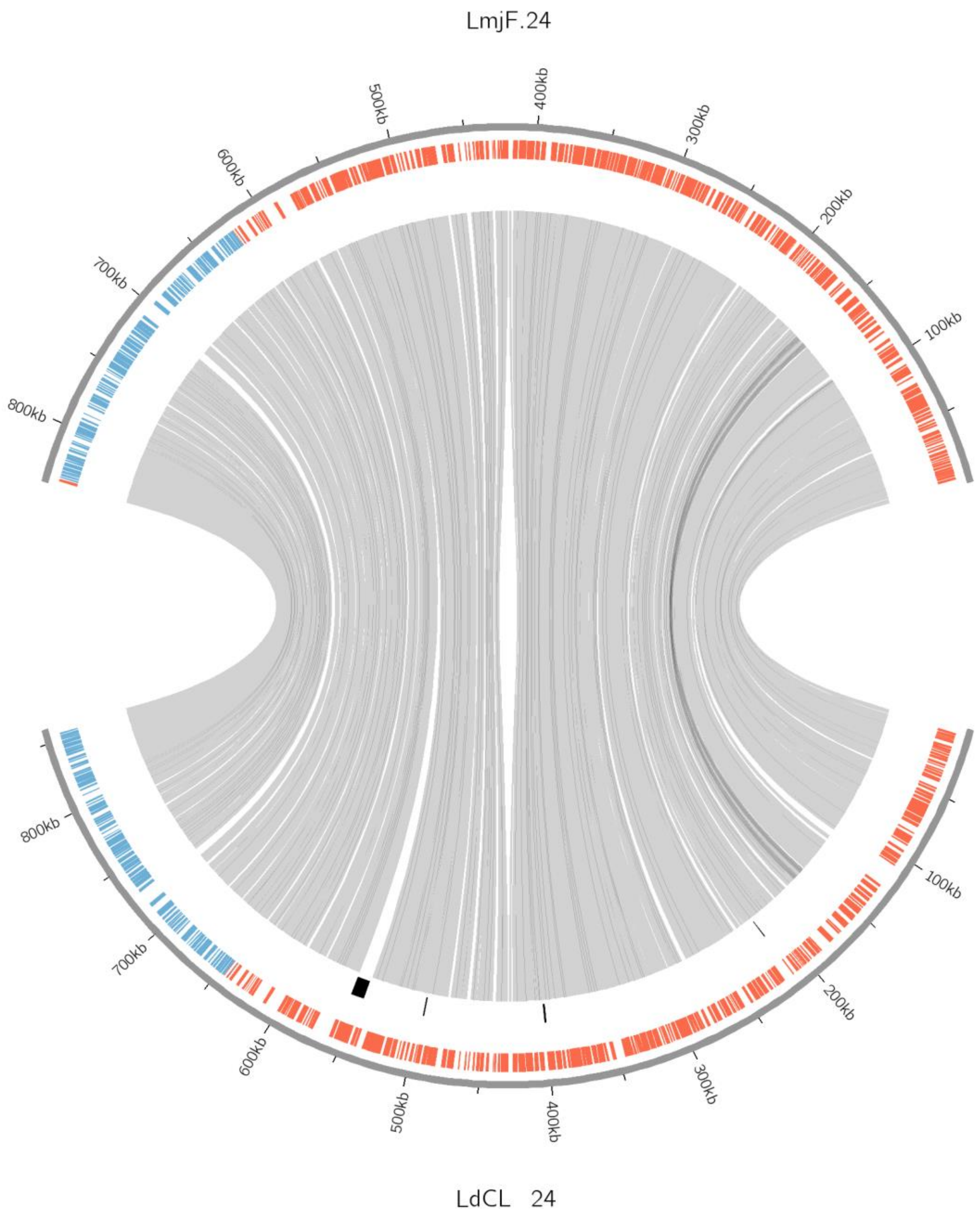

LmjF.24
100kb
200kb
300kb
400kb
500kb
600kb
700kb
800kb
100kb
200kb
300kb
400kb
500kb
600kb
700kb
800kb
LdCL 24

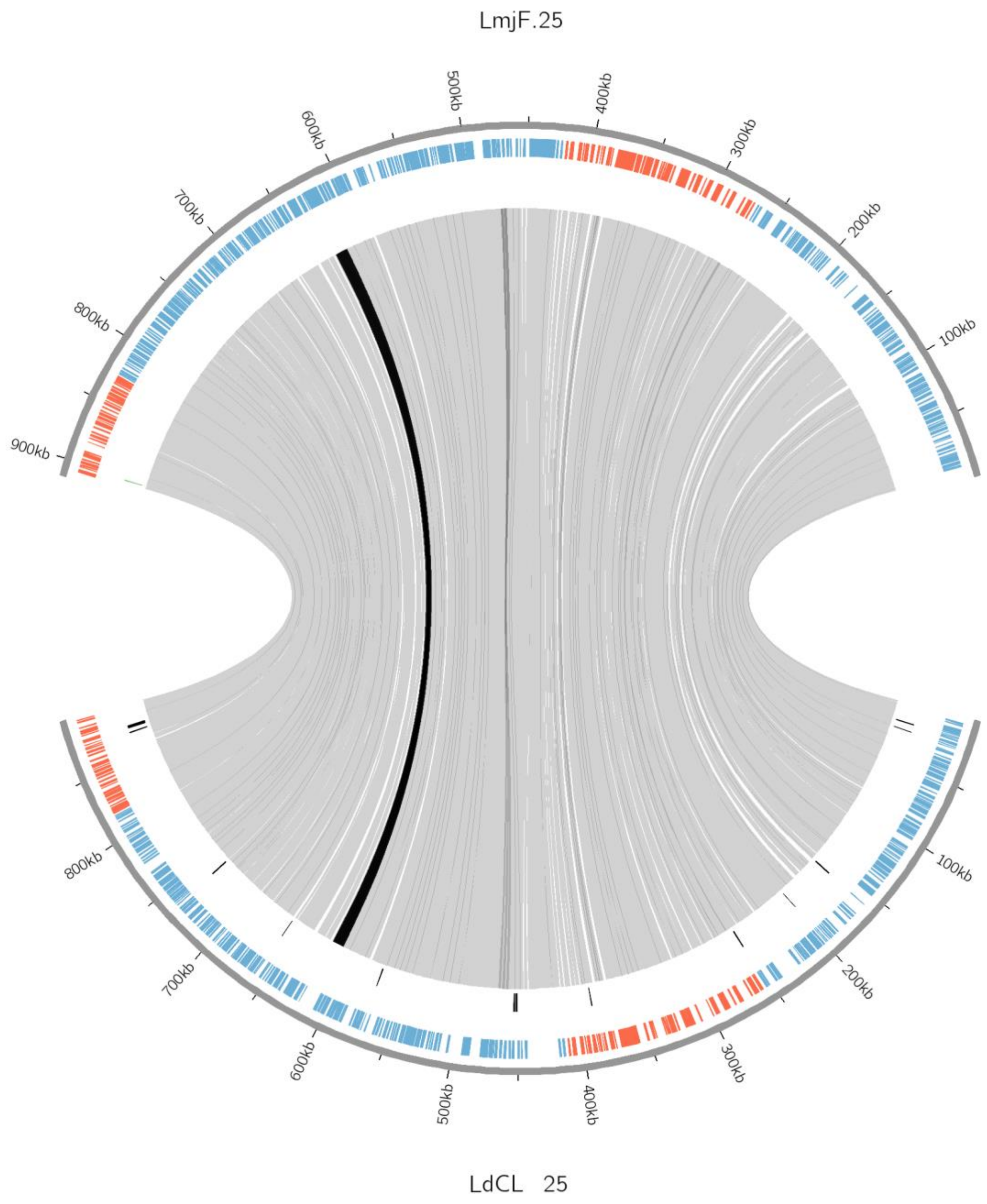
LmjF.25
100kb
200kb
300kb
400kb
500kb
600kb
700kb
800kb
900kb
LdCL 25
100kb
200kb
300kb
400kb
500kb
600kb
700kb
800kb

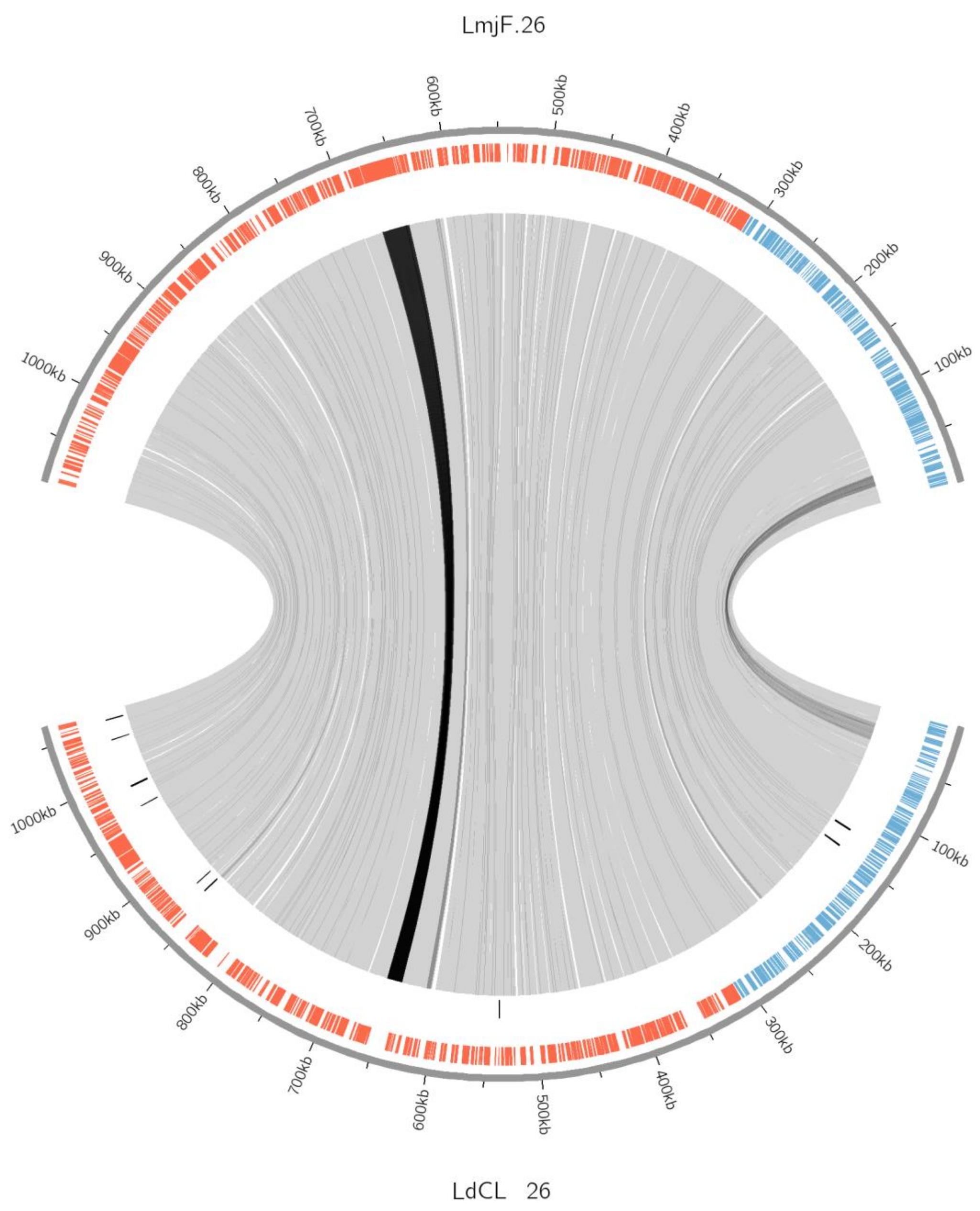
LmjF.26
100kb
200kb
300kb
400kb
500kb
600kb
700kb
800kb
900kb
1000kb
LdCL 26

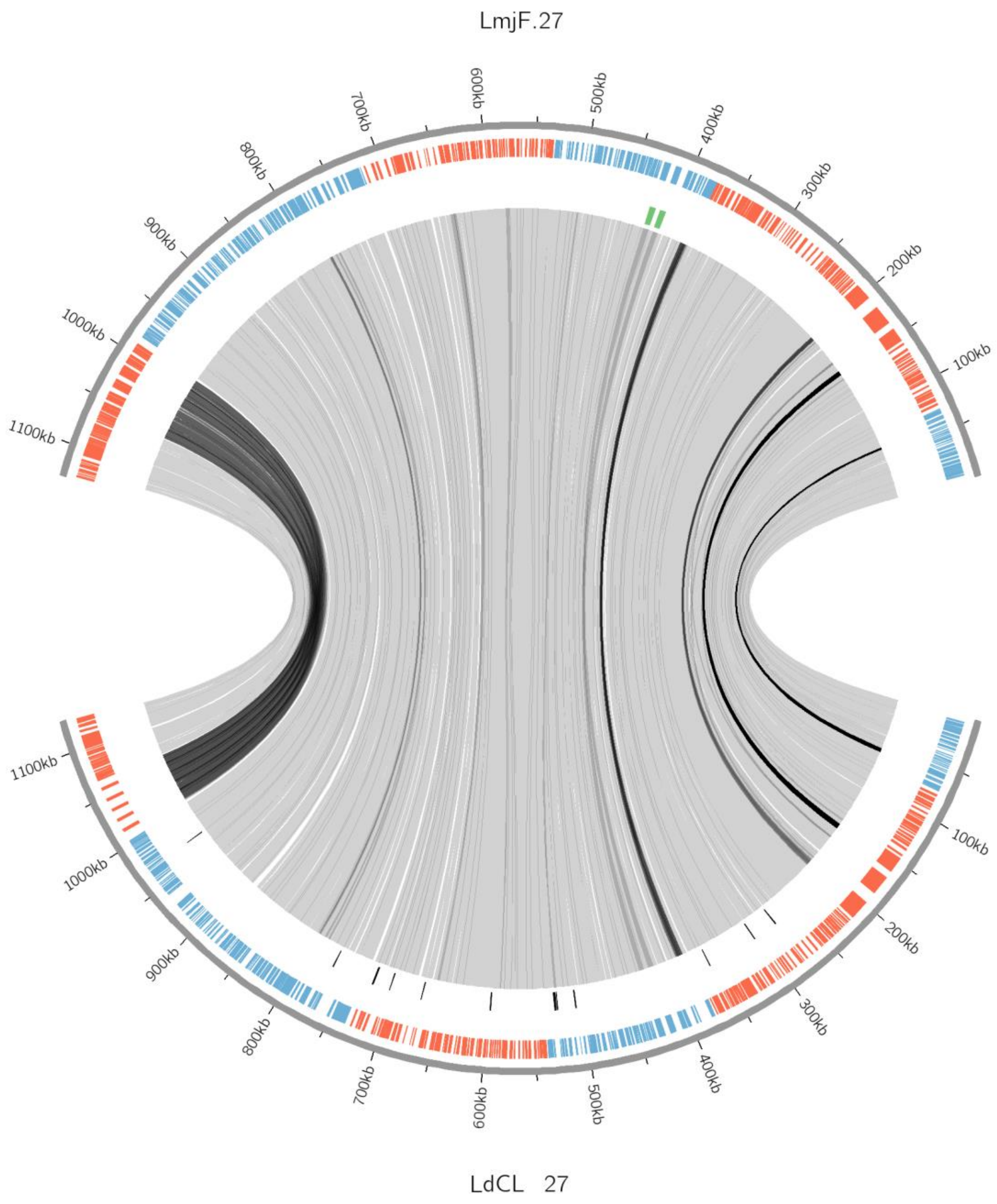
LmjF.27
100kb
200kb
300kb
400kb
500kb
600kb
700kb
800kb
900kb
1000kb
1100kb
LdCL 27

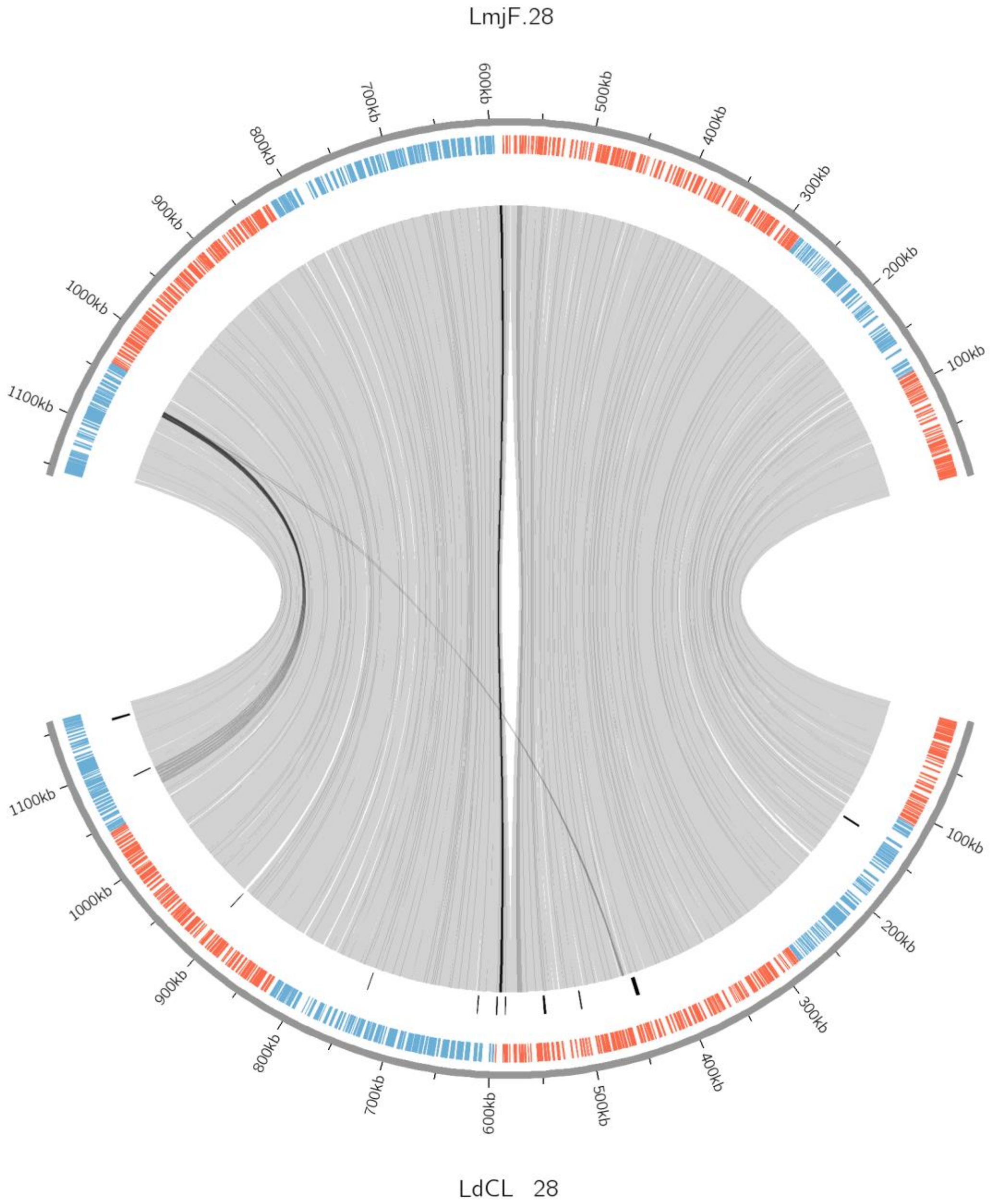
LmjF.28
600kb
500kb
400kb
300kb
200kb
100kb
700kb
800kb
900kb
1000kb
1100kb
LdCL 28

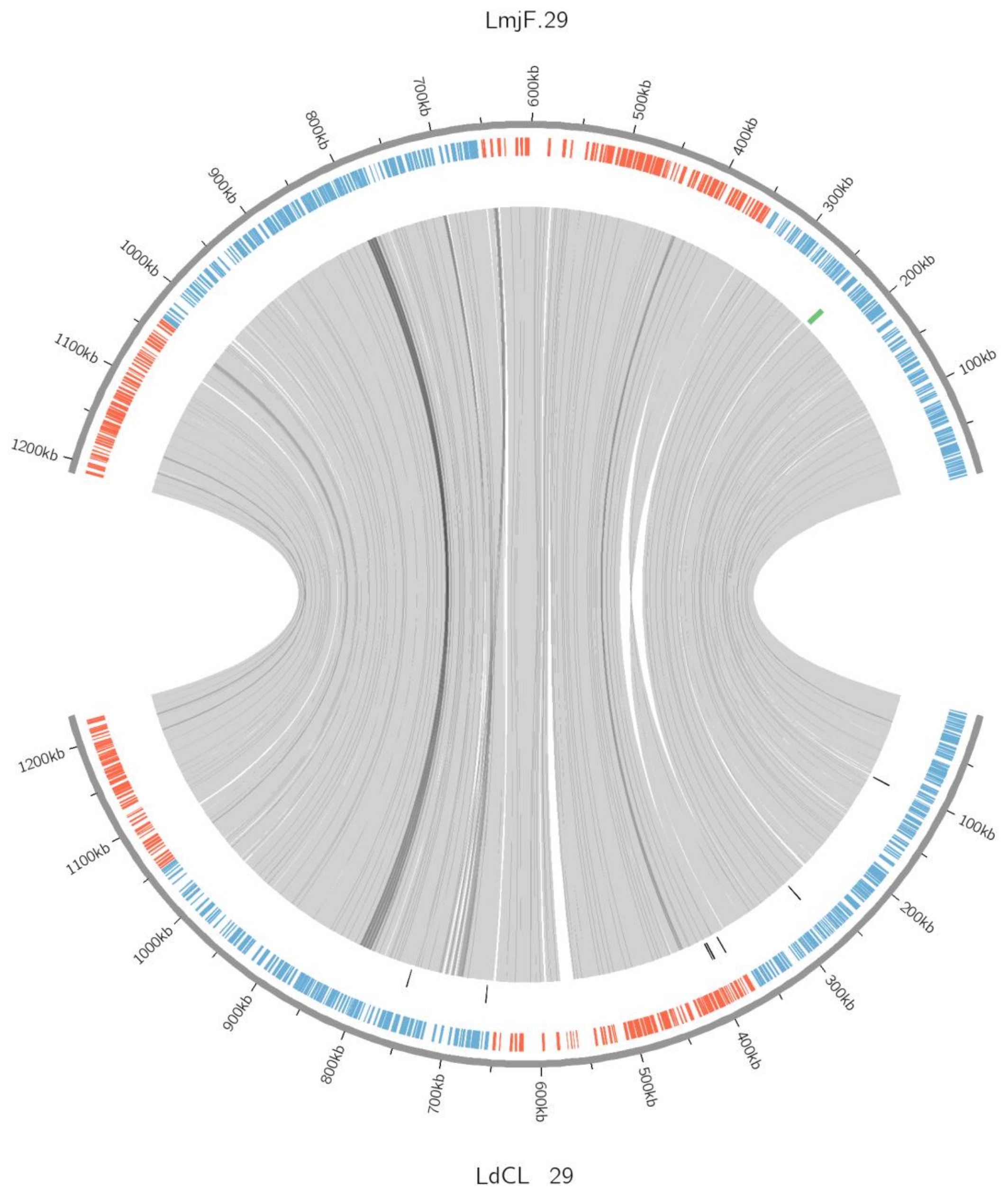
LmjF.29
600kb
700kb
800kb
900kb
1000kb
1100kb
1200kb
500kb
400kb
300kb
200kb
100kb
LdCL 29

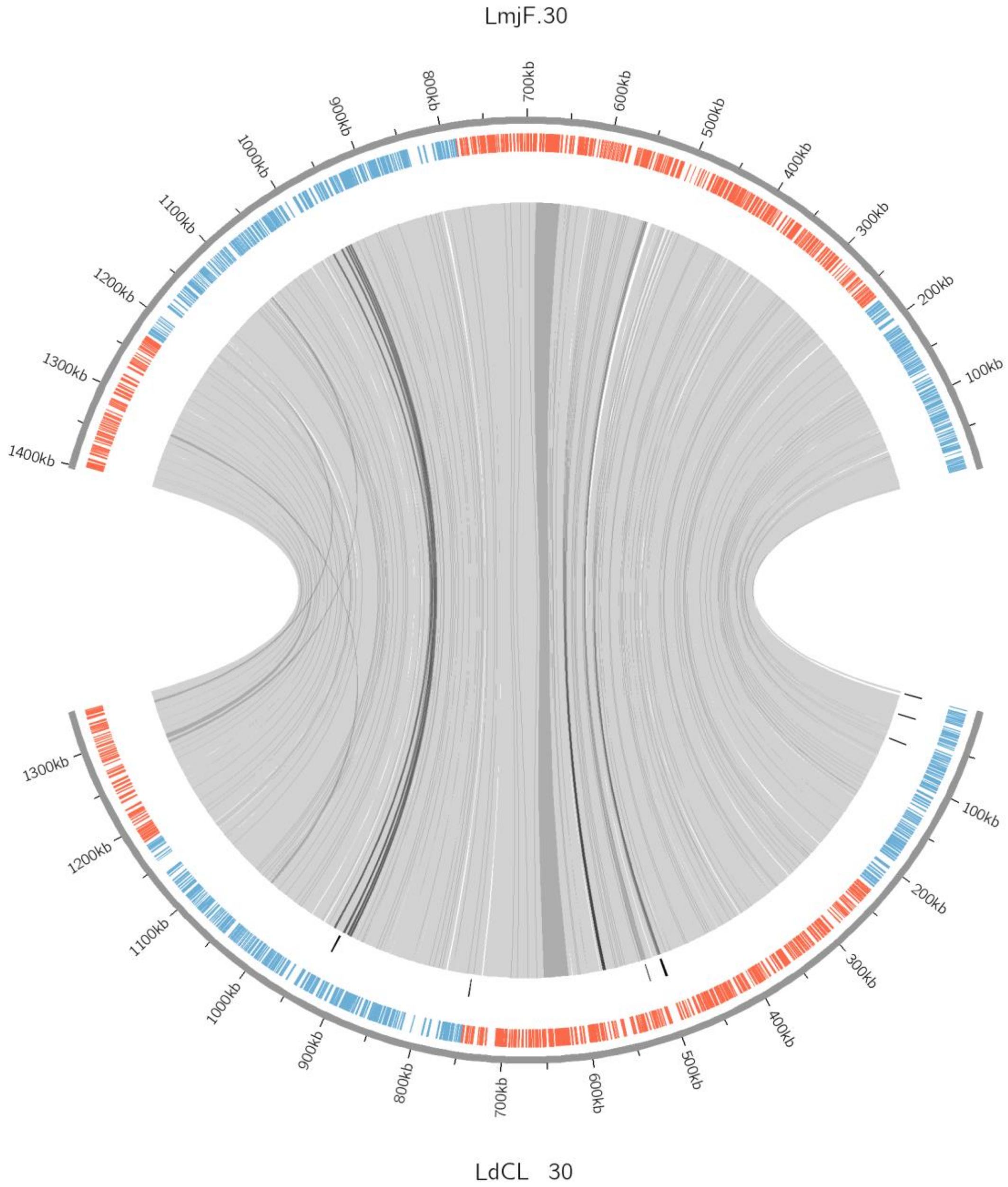

LmjF.30
100kb
200kb
300kb
400kb
500kb
600kb
700kb
800kb
900kb
1000kb
1100kb
1200kb
1300kb
1400kb
LdCL 30

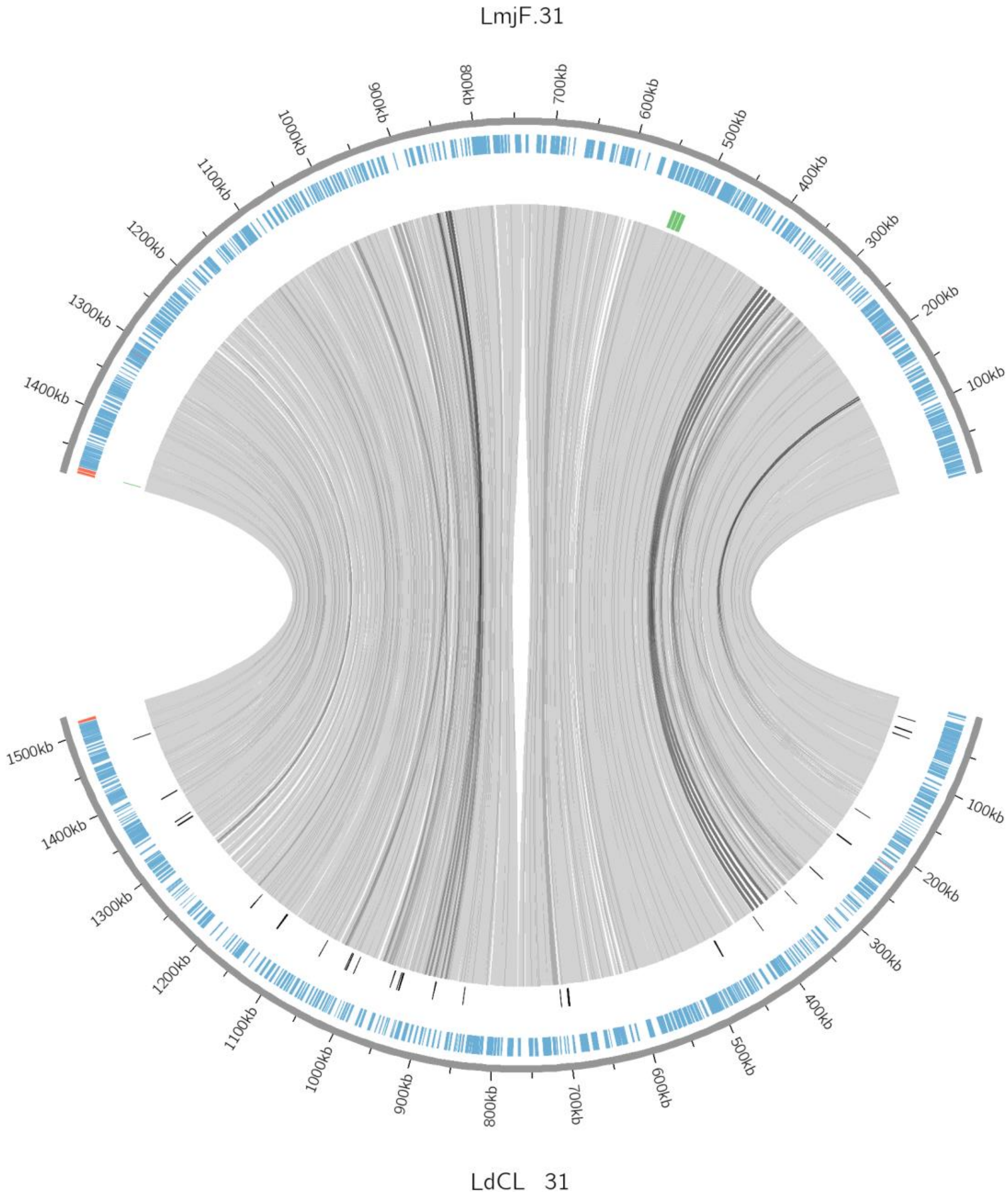
LmjF.31
100kb
200kb
300kb
400kb
500kb
600kb
700kb
800kb
900kb
1000kb
1100kb
1200kb
1300kb
1400kb
100kb
200kb
300kb
400kb
500kb
600kb
700kb
800kb
900kb
1000kb
1100kb
1200kb
1300kb
1400kb
1500kb
LdCL 31

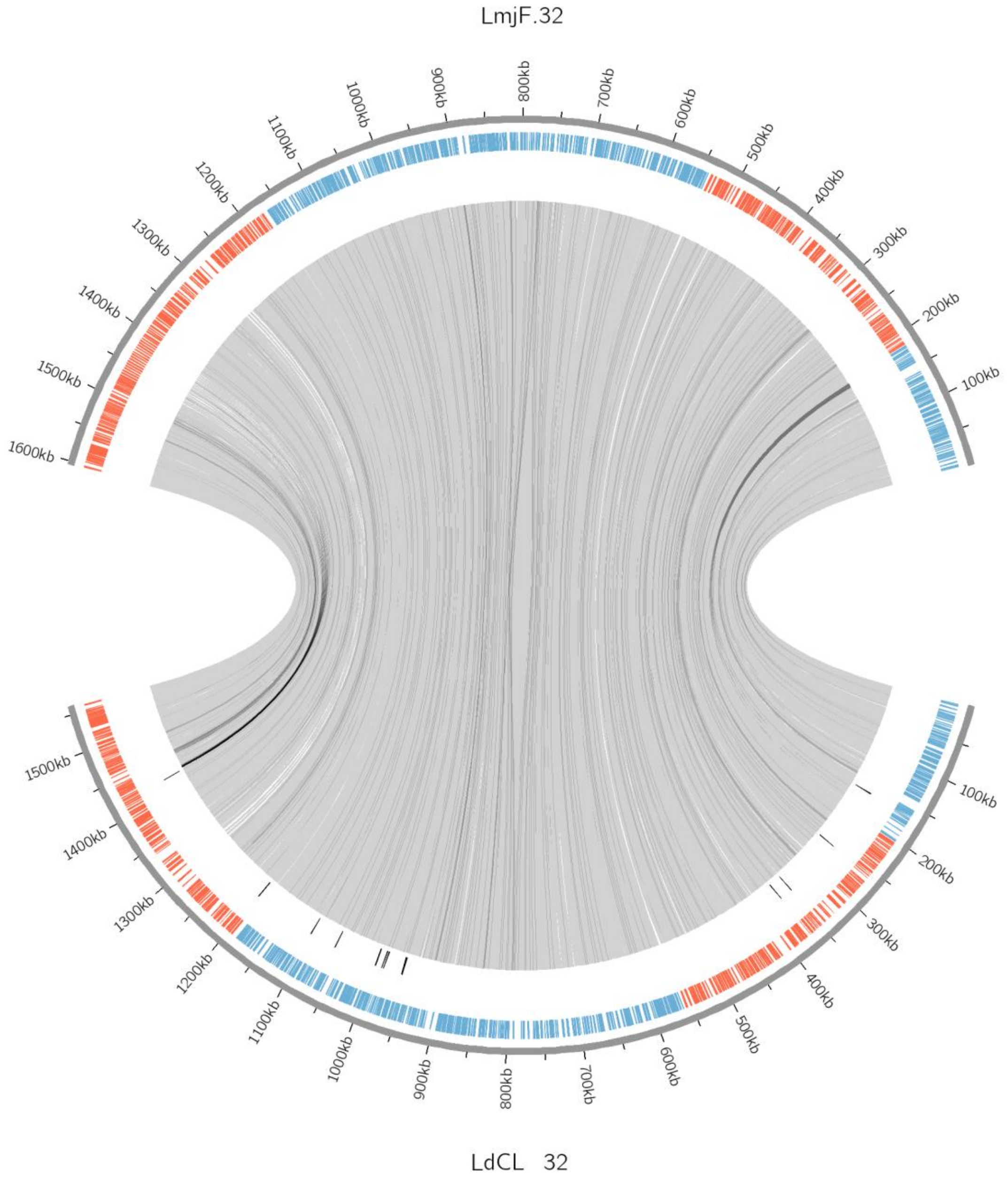
LmjF.32
100kb
200kb
300kb
400kb
500kb
600kb
700kb
800kb
900kb
1000kb
1100kb
1200kb
1300kb
1400kb
1500kb
1600kb
100kb
200kb
300kb
400kb
500kb
600kb
700kb
800kb
900kb
1000kb
1100kb
1200kb
1300kb
1400kb
1500kb
LdCL 32

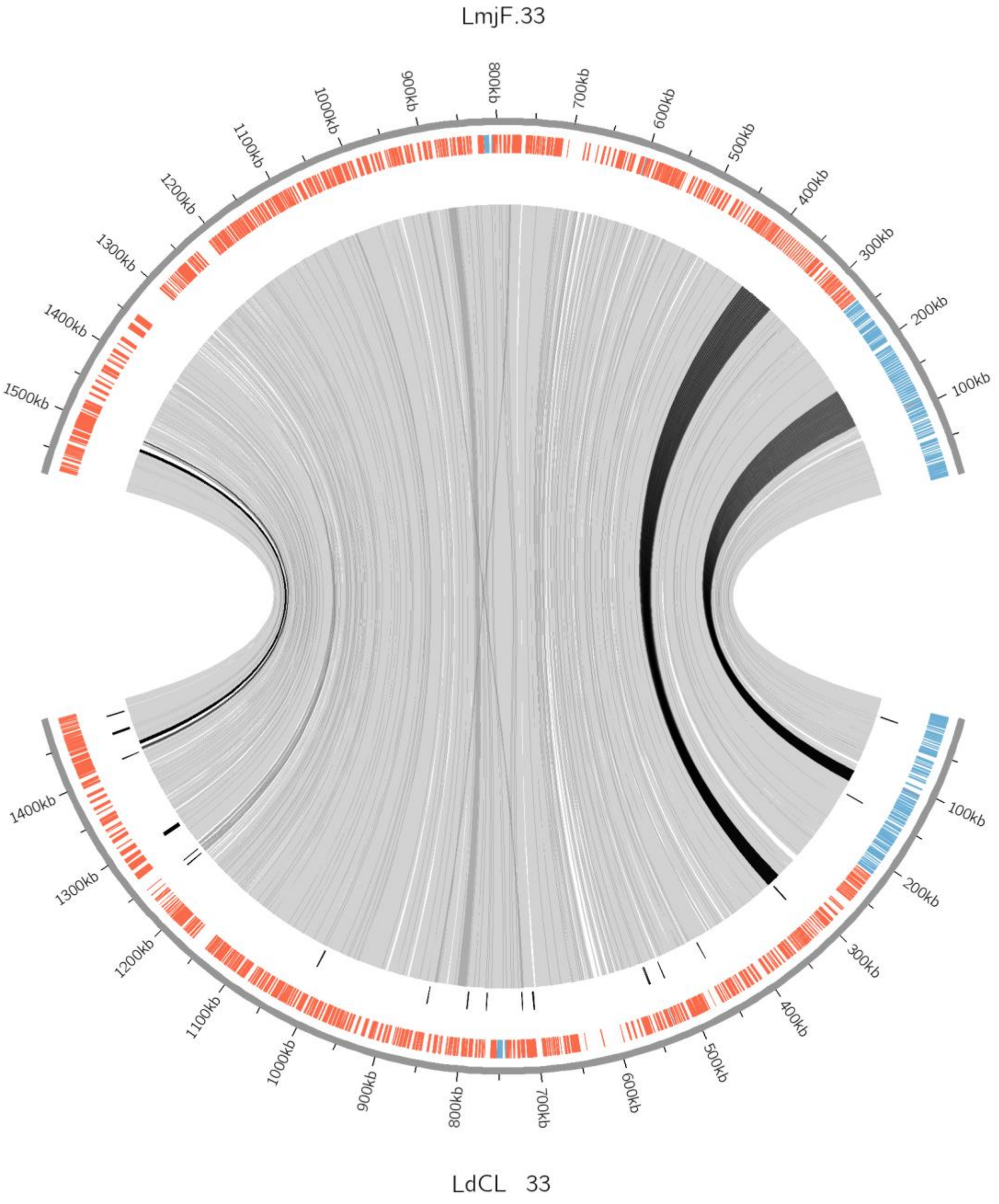

LmjF.33
100kb
200kb
300kb
400kb
500kb
600kb
700kb
800kb
900kb
1000kb
1100kb
1200kb
1300kb
1400kb
1500kb
LdCL 33
100kb
200kb
300kb
400kb
500kb
600kb
700kb
800kb
900kb
1000kb
1100kb
1200kb
1300kb
1400kb

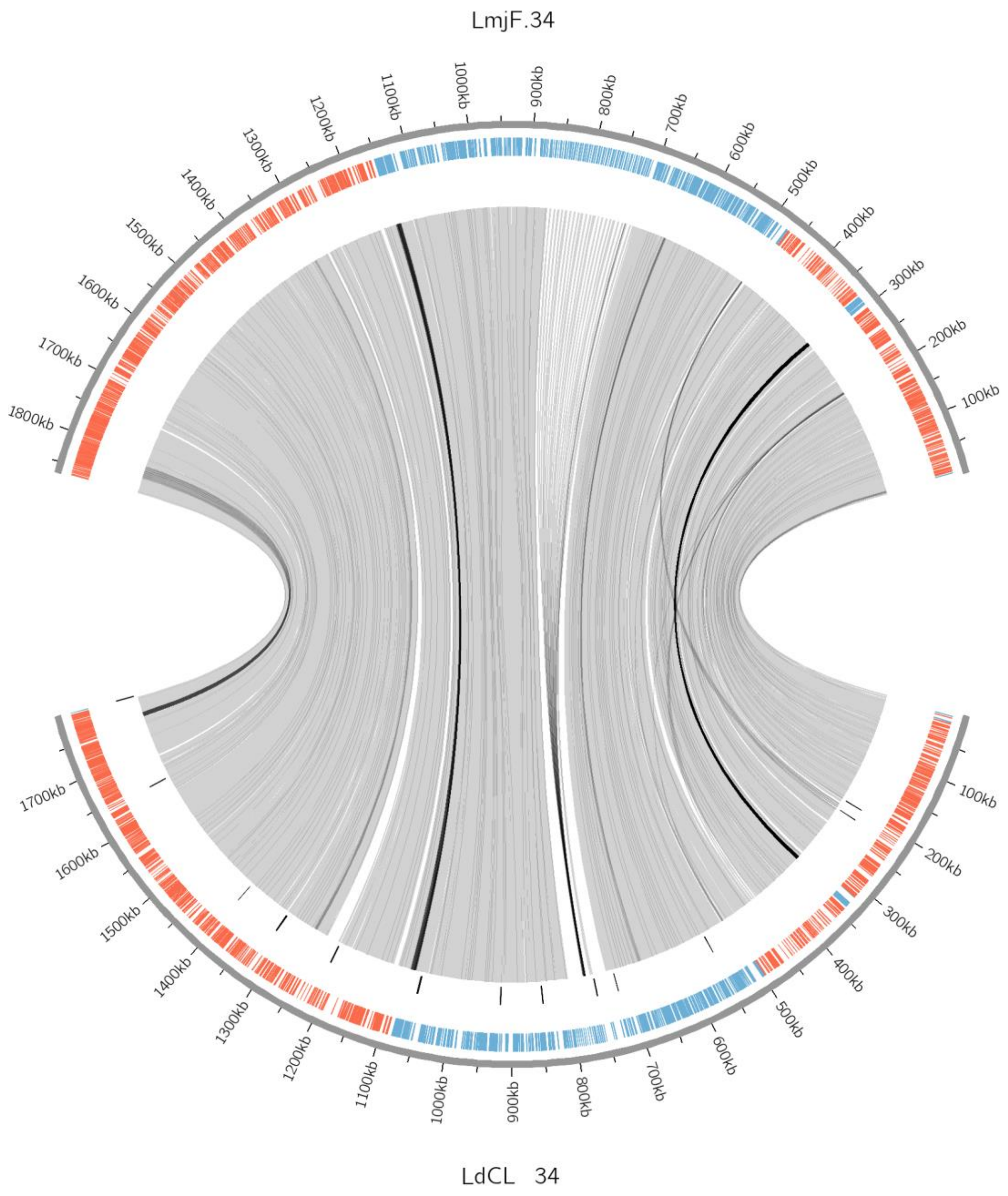
LmjF.34
100kb
200kb
300kb
400kb
500kb
600kb
700kb
800kb
900kb
1000kb
1100kb
1200kb
1300kb
1400kb
1500kb
1600kb
1700kb
1800kb
LdCL 34

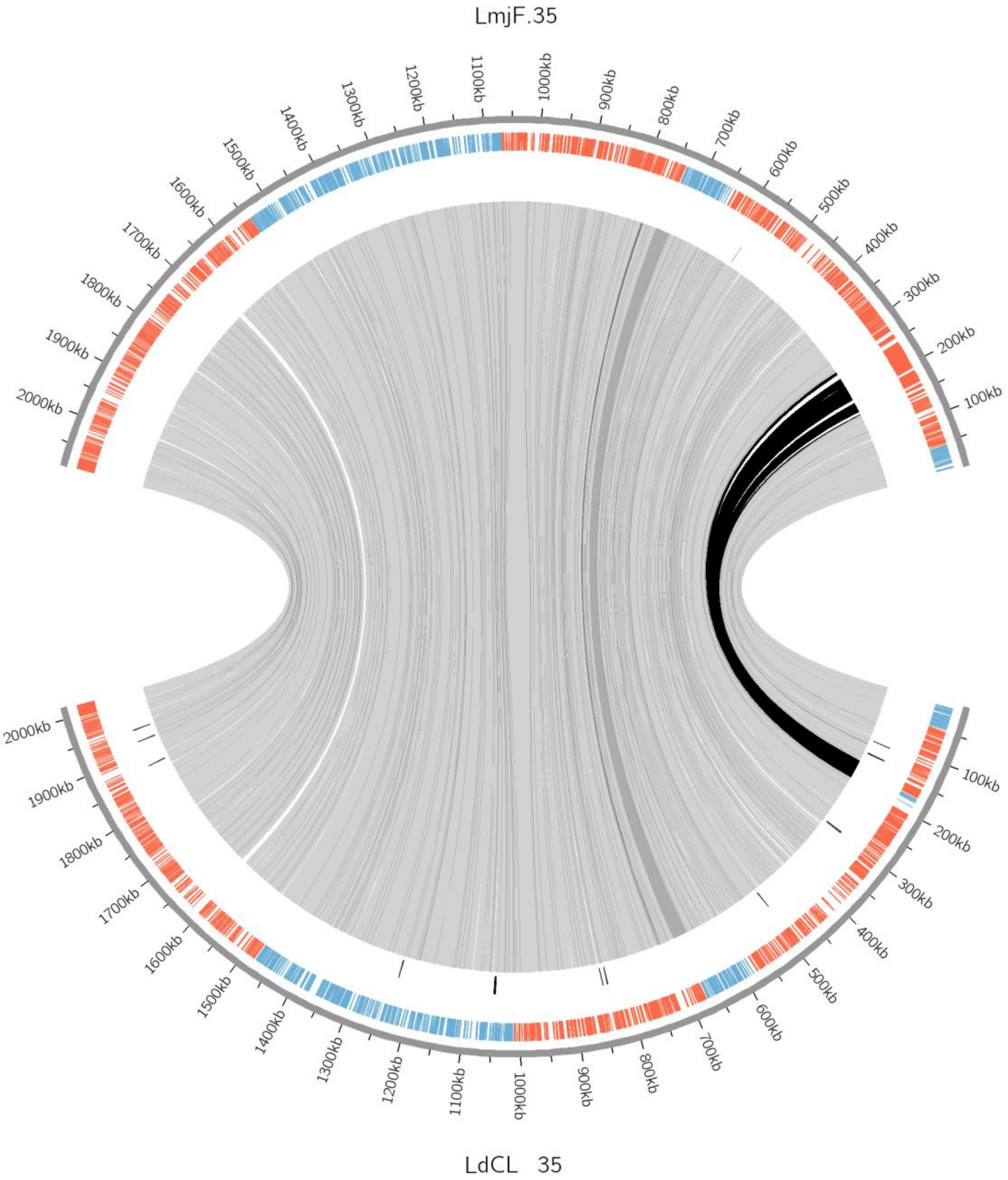

LmjF.35
100kb
200kb
300kb
400kb
500kb
600kb
700kb
800kb
900kb
1000kb
1100kb
1200kb
1300kb
1400kb
1500kb
1600kb
1700kb
1800kb
1900kb
2000kb
LdCL 35

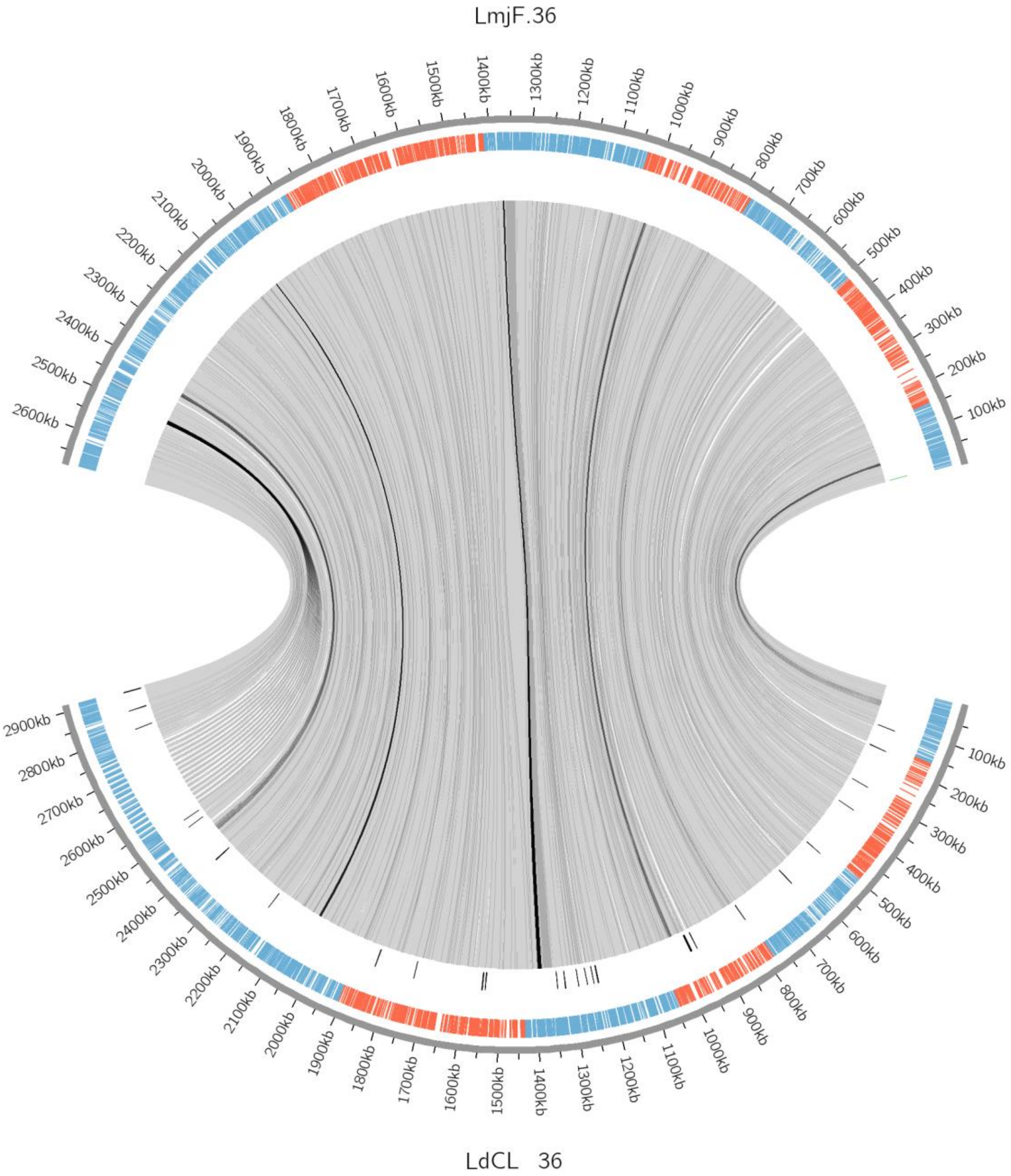

Annotations from both species were aligned to their respective genome and compared to each other. Each chromosome is represented as a half circle. ORFs are colored according to their coding strands. Black lines represent pairs of homologous genes between *L. major* (top) and attenuated cutaneous *L. donovani* (bottom).

Supplementary Figure S4. Confirmation of a 25kb deletion on chromosome 36

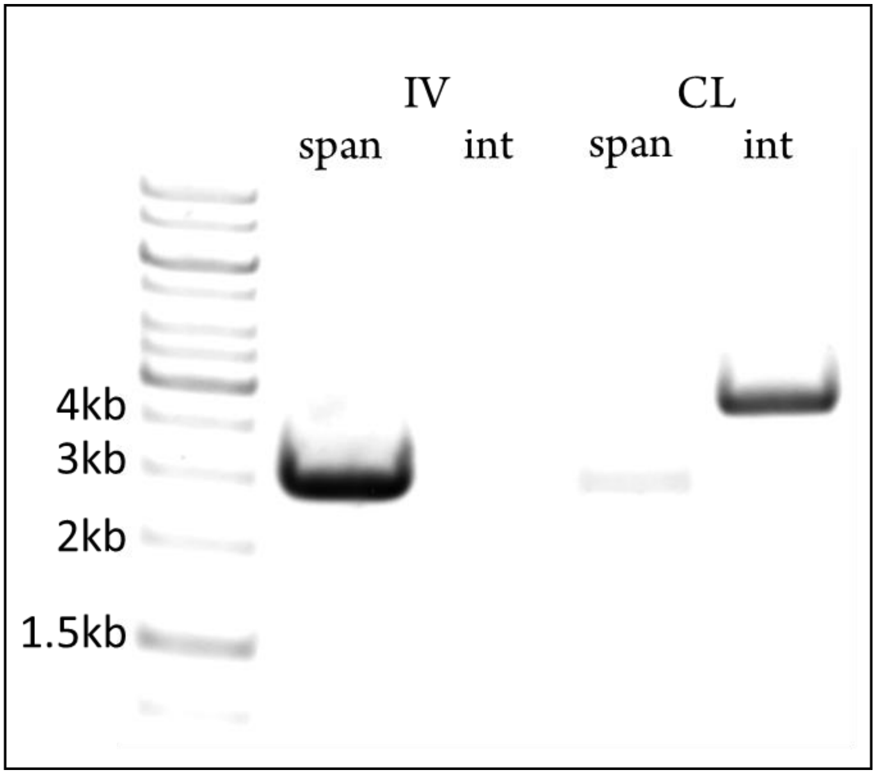

Primers were designed to amplify a fragment spanning across the deletion (span) and to amplify a region internal to the deletion (int). The IV strain only produced an amplicon from either end of the deletion indicating the 28kb region collapsed to 3kb but was unable to produce any amplicon internal to the deleted region, indicating the 25kb region was absent rather than translocated. The CL strain mainly produced the internal region as the entire 28kb fragment is too long to be efficiently resolved by PCR and a faint band also appeared in the span reaction indicating the presence of some cells within the population that also contained the deletion.

Supplementary methods S1

```
Relaxed.spec:
merSize=14
asmOvlErrorRate=0.10
asmUtgErrorRate=0.10
asmCnsErrorRate=0.10
asmCgwErrorRate=0.10
asmOBT=1
asmObtErrorRate=0.08
asmObtErrorLimit=4.5
utgGraphErrorRate=0.05
utgMergeErrorRate=0.05
ovlHashBits=26
ovlHashLoad=0.80
sensitive = 1
maxCoverage=60
gridOptionsOverlap = -pe threads 16 -l mem=2GB
gridOptionsConsensus = -pe threads 16
gridOptionsScript = -pe threads 5
merylThreads = 60
ovlThreads = 60
merylMemory = 12000
ovlStoreMemory = 12000
ovlConcurrency = 1
mbtThreads=60
merOverlapperThreads=60
frgCorrThreads=60
batThreads=60
```
